# Trait‐specific sensitive developmental windows: Wing growth best integrates weather conditions encountered throughout the development of nestling Alpine swifts

**DOI:** 10.1002/ece3.11491

**Published:** 2024-06-06

**Authors:** Giulia Masoero, Michela N. Dumas, Julien G. A. Martin, Pierre Bize

**Affiliations:** ^1^ Swiss Ornithological Institute Sempach Switzerland; ^2^ Department of Biology University of Ottawa Ottawa Ontario Canada

**Keywords:** Apodiformes, climate change, early‐life conditions, feathers, heat stress, meteorological conditions, multi‐trait, offspring development, *Tachymarptis* *(Apus)* *melba*

## Abstract

The size and growth patterns of nestling birds are key determinants of their survival up to fledging and long‐term fitness. However, because traits such as feathers, skeleton and body mass can follow different developmental trajectories, our understanding of the impact of adverse weather on development requires insights into trait‐specific sensitive developmental windows. We analysed data from nestling Alpine swifts in Switzerland measured throughout growth up to the age of 50 days (i.e. fledging between 50 and 70 days), for wing length and body mass (2693 nestlings in 25 years) and sternum length (2447 nestlings in 22 years). We show that the sensitive developmental windows for wing and sternum length corresponded to the periods of trait‐specific peak growth, which span almost the whole developmental period for wings and the first half for the sternum. Adverse weather conditions during these periods slowed down growth and reduced size. Although nestling body mass at 50 days showed the greatest inter‐individual variation, this was explained by weather in the two days before measurement rather than during peak growth. Interestingly, the relationship between temperature and body mass was not linear, and the initial sharp increase in body mass associated with the increase in temperature was followed by a moderate drop on hot days, likely linked to heat stress. Nestlings experiencing adverse weather conditions during wing growth had lower survival rates up to fledging and fledged at later ages, presumably to compensate for slower wing growth. Overall, our results suggest that measures of feather growth and, to some extent, skeletal growth best capture the consequences of adverse weather conditions throughout the whole development of offspring, while body mass better reflects the short, instantaneous effects of weather conditions on their body reserves (i.e. energy depletion vs. storage in unfavourable vs. favourable conditions).

## Graphical abstract

Using data from more than 2000 Alpine swift nestlings with measurements spanning more than 20 years, we show that adverse weather conditions during the sensitive developmental windows for wing and sternum length result in slower growth, reduced size at 50 days (i.e. close to fledging), delayed fledging and lowered fledging success. Variation in nestling body mass at 50 days was explained by the weather in the two days before measurement rather than during peak growth. Overall, our results suggest that measures of feather growth and, to some extent, skeletal growth best capture the consequences of adverse weather conditions throughout the whole development of offspring, while body mass better reflects the short, instantaneous effects of weather conditions on their body reserves.

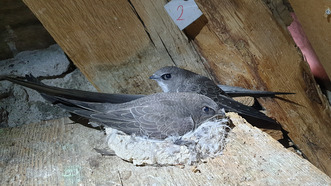

## 1 INTRODUCTION

In most animal species, adult size and mass are key life history traits, with larger and heavier individuals often having greater opportunities to monopolise resources (Basset & Angelis, 2007) and, in turn, greater survival and greater lifetime reproductive success (Baker et al., 2015; Brown et al., 1993; Speakman, 2005). Furthermore, in species with determinate growth, where growth ceases at independence and/or after sexual maturity, adult size and mass are primarily influenced by environmental conditions experienced during development. Therefore, environmental conditions experienced during early life can have long‐lasting consequences on the phenotypes displayed by those same individuals in adulthood and their fitness (Bateson, 1979; Cooper & Kruuk, 2018; English et al., 2016; Lindström, 1999; Metcalfe & Monaghan, 2001). With the acceleration of climatic changes, it has become paramount to understand the consequences of weather conditions during early development on phenotypes and whether all phenotypic traits are similarly affected (Noble et al., 2018; Sauve et al., 2021). Indeed, different phenotypic traits may contribute differently to fitness. We can therefore expect a hierarchy of protection and compensation between traits according to their contribution to fitness at a given stage (Bize et al., 2006; Metcalfe & Monaghan, 2001). That is, when weather conditions are unfavourable and resources become limited, resources may be preferentially allocated to traits that contribute most immediately to fitness (i.e. hierarchy of protection). Furthermore, should conditions improve, some traits can accelerate in growth to compensate for the initial setback, with the strongest allocation of resources and compensation seen again in traits with the most substantial and immediate contribution to fitness (i.e. hierarchy of compensation).

In birds, inclement weather conditions encountered early in life can strongly affect the growth and survival of offspring (e.g. Arnold et al., 2007; de Zwaan et al., 2019; Donelson et al., 2009; Hegyi & Török, 2007). Birds have a determined growth and start their life as ectothermic, with altricial species only beginning to express endothermic traits 1 to 3 weeks after hatching. So, in addition to the impact of weather conditions on food resources (Arbeiter et al., 2016; Grüebler et al., 2008; Price & Dzialowski, 2018), cold and rainy days pose strong thermoregulatory challenges for growing individuals. Indeed, the development of endothermy requires high energy investment, explained by the need to supply sufficient nutrients and oxygen to heat‐generating tissues. These tissues include thermogenic sites, such as skeletal muscles, and internal organs that supply the muscles with oxygen and nutrients, such as the heart, lungs and liver. Prioritising the investment in thermoregulation and the development of these highly metabolically active tissues, and thus overall mass increase can help cope with thermoregulatory challenges (Arendt, 1997; Price & Dzialowski, 2018). High energy investment in maintaining endothermy can, however, hinder the development of other traits, such as skeletal or feather growth (Olson, 1992; Węgrzyn, 2013). In line with this, nestlings from experimentally heated nests have been shown to grow faster, suggesting that optimal conditions can help lessen the energetic burden of thermoregulation (Dawson et al., 2005). Greater investment in body mass is also expected because, to survive prolonged periods of food shortage during adverse weather conditions, organisms must rely on lipids stored in adipose tissues and on proteins catabolised from internal organs such as pectoral muscle and gut, and all these tissues contribute to body mass. Investment in body mass (to sustain endothermy and ensure sufficient energy reserves) generally occurs at the expense of skeletal and feather growth, with skeletal growth often taking priority over feather growth. Body size, and thereof skeletal size, can have important immediate fitness consequences on the ability of nestlings to compete with siblings (e.g. Nilsson & Gårdmark, 2001). Feathers, on the other hand, can be replaced and repaired later in life through moulting, and flight feathers only lead to significant fitness benefits very late in the bird's development, when it is ready to fly. Finally, by slowing down the growth of nestlings, adverse weather conditions can prolong their development and delay the age at fledging, and, if adverse conditions persist, affect the chances of survival before fledging (e.g. Dawson et al., 2005; de Zwaan et al., 2022).

**FIGURE 1:**
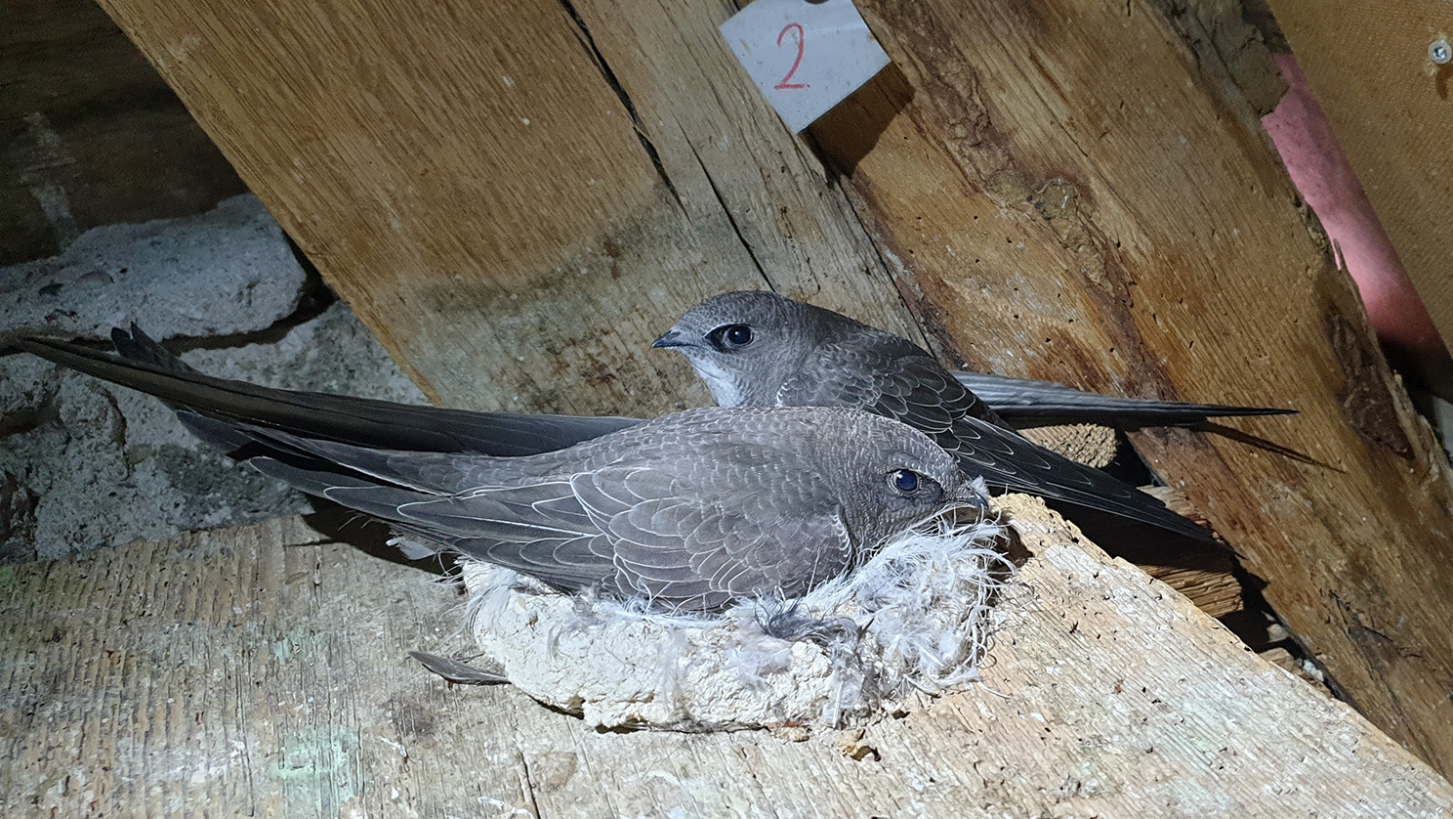
Two Alpine swift nestlings at ca. 50 days of age. Photo by G. Masoero.

Here, we use 25 years of data on body mass and wings and 22 years of data on sternum length of nestling Alpine swifts (*Tachymarptis melba*; Figure 1) to investigate how adverse weather conditions affected their growth and size before fledging, as well as age at fledging and survival up to fledging. The Alpine swift is an insectivorous bird that feeds exclusively on prey caught while flying. Thus, its ecology and reproductive success are strongly dependent on weather conditions (Arn‐Willi, 1960; Bize et al., 2007). In agreement with this, previous studies have shown that the body temperature, mass and pectoral muscle size of Alpine swift nestlings are lower in adverse weather conditions than in good weather (Bize et al., 2007) and that this species has a hierarchy of tissue preservation in response to undernutrition, from body mass to skeletal and wing growth (Bize et al., 2006). Hence, we expected that, in the Alpine swift, the size of 50‐day‐old nestlings, which is close to fledging (between 50 and 70 days of age), is significantly influenced by the weather conditions experienced during growth, with the strongest reduction in size in response to adverse weather seen on wing length, followed by sternum length and body mass. As recently documented in adult Alpine swifts (Dumas et al., 2024), we expected that variation in nestling body mass would reflect immediate (past days) variation in weather conditions and food availability. By contrast, we expected that variation in nestling wing and sternum lengths to be associated with weather conditions experienced during a more extended developmental period, with the exact developmental window differing between traits since the sternum stops growing earlier than the wings. Finally, we expected that adverse weather conditions could delay fledging, especially if wing development is slowed down in this highly aerial bird (Bize et al., 2003), and contribute to greater nestling mortality before fledging.

## 2 MATERIALS AND METHODS

### 2.1 Study system

Data were collected between 1999 and 2023 in a Swiss population of Alpine swift. It is a long‐distance migratory bird that breeds in colonies of a few to several hundred pairs in holes within cliffs or under the roofs of tall buildings. In Switzerland, Alpine swifts return to their breeding grounds from sub‐Saharan Africa at the beginning of April (Meier et al., 2020) and start laying eggs between early May and June, with significant adaptive variations in laying dates depending on weather conditions (de Villemereuil et al., 2020). Females lay one clutch a year, with one to four eggs per clutch (modal clutch size is three). Both parents incubate the eggs for about 18 days and then feed their nestlings until fledging, which occurs around 55 days after hatching (range 50–76 days; (Bize et al., 2004) and this manuscript). After breeding, Alpine swifts migrate back to Africa in September (Meier et al., 2020).

Fieldwork was carried out in two Alpine swift colonies located in clock towers in the Swiss cities of Biel (60–100 breeding pairs) and Solothurn (40–55 breeding pairs), ca. 20 km apart (map in Data S1). Each year, both colonies were regularly visited to monitor egg laying and clutch size, to capture and measure adults and to ring and measure nestlings. Nestlings were individually recognised by ringing them with numbered metal rings 10–15 days after hatching. Nestlings were measured regularly (usually every 5–10 days, on average five times) until fledging. At each measurement, we measured wing length with a ruler to the nearest 1 mm, sternum size with a calliper to the nearest 0.1 mm and body mass with a digital scale to the nearest 0.1 g. The measure of sternum length provides an estimate of skeletal growth and size. Tarsus length has been commonly used in passerines, but it is difficult to measure in a repeatable way in a species with short and bulky tarsi, such as swifts. As nestlings are not ringed at hatching, the age of the nestlings in a brood is based on the hatching date of the first nestling; the last nestling is usually born on the same day or 1 day later. Therefore, measurements for a brood of three nestlings, for example, are taken when the first‐hatched nestling reaches 50 days of age, the youngest one might be the same age or 1 day younger. Only nestlings that survived up to fledging were included in the statistical analyses. Sample sizes differ between traits, as wing length and body mass have been measured since 1999, while sternum length has been measured since 2003.

### 2.2 Weather data

To estimate the weather conditions during nestling development, we used meteorological data collected from five Swiss meteorological stations surrounding Biel and Solothurn (Bern‐Zollikofen, Cressier, Grenchen, Koppigen and Wynau; map in Data S1). Doing so allowed us to cover the whole foraging area of the swifts (parents forage within a 15 km radius around their breeding colony; Alexandra Brighten et al. unpublished results from GPS loggers; Arn‐Willi, 1960) and to account for microenvironmental variations (i.e. strong weather events captured by one station only). Daily weather data were averaged across the five stations to obtain three variables: mean daily temperature (average air temperature at 2 m above ground for the whole day), daily precipitation (total rainfall for that day) and wind speed (daily mean of the wind speed scalar in m/s). We also used a principal component analysis to calculate a daily first component (PC1) between temperature and precipitation for the meteorological data collected during the whole breeding season (May–August). PC1 explained 60% of the total variance in weather data, with factor loadings of 0.71 for the mean temperature and −0.71 for the mean precipitation. A high PC1 value, therefore, indicates warm and dry weather, whereas low values indicate cold and rainy weather.

### 2.3 General statistical methods

All analyses were performed in R version 4.3.3 (R Core Team, 2024). Linear mixed‐effect models (LMMs) and generalised linear mixed‐effect models (GLMMs) were run using the packages *lme4* v.1.1‐35.1 (Bates et al., 2015) and *lmerTest* v.3.1‐3 (Kuznetsova et al., 2016). Before fitting the models, the variable age of nestlings in days was centered to 50 (age ‐ 50), and the variable day of hatching was mean centred (*μ* = 0) and standardised to a standard deviation of 1 (*σ* ^2^ = 1).

### 2.4 Weather effects on nestling size

We investigated the relative importance of three meteorological factors describing the weather in the study area during the breeding season in explaining the variation in the size at 50 days of the three traits (wing, sternum and body mass). As meteorological variables, we tested: mean daily temperature, daily precipitation, wind speed and PC1. We used the R package *climwin* v.1.2.3 (van de Pol et al., 2016) to perform a sliding window analysis. This allowed comparing models with a meteorological signal with a baseline model for each trait (defined as the model without the weather signal). The baseline models were LMMs that controlled for the effect of variables that generally can influence nestlings' growth in birds: brood size at hatching, hatching day (using May 1 as day 1), age and colony (Solothurn or Biel) as fixed effects. Nestlings from large broods are known to grow slower and show smaller values for the traits (e.g. Bize et al., 2010; De Kogel, 1997; Nur, 1984) and late‐hatching birds might have lower growth rates due to reduced food availability later in the season (e.g. Van Noordwijk et al., 1995). As chicks were not always measured precisely at 50 days, we used measures taken between 45 and 55 days of age and added age in days as an explanatory variable in the models to control for this variation. As random effects, we included brood ID and year as factors to account for the non‐independence of nestlings belonging to the same brood and same cohorts respectively.

**FIGURE 2:**
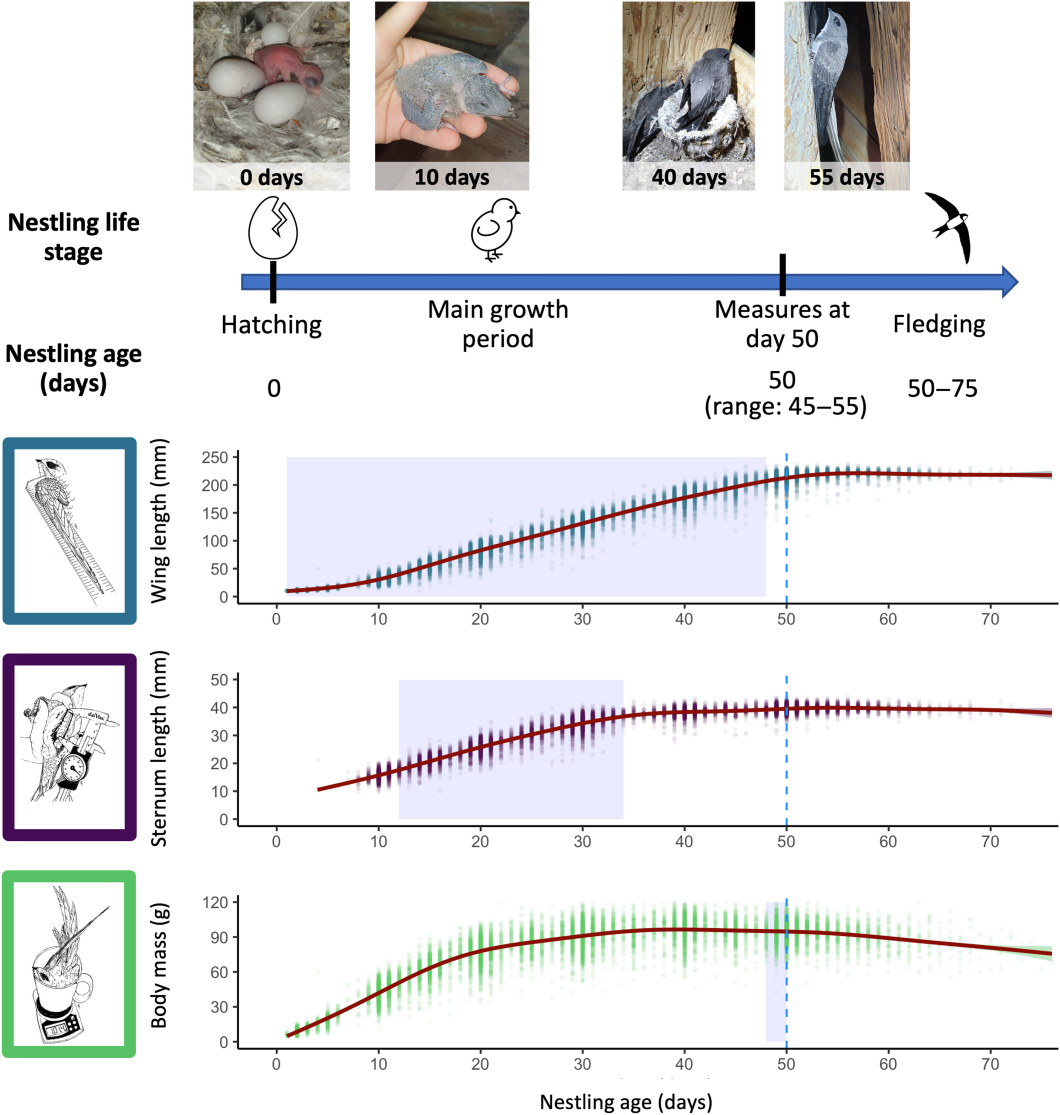
Developmental trajectories, from hatching to fledging, of nestling Alpine swifts. Dots represent individual measurements of wing and sternum length and body mass of nestlings measured between 1999 and 2023, and smooth lines represent the average growth pattern of nestlings. The sensitive developmental windows during which weather conditions most affected the phenotype at 50 days (see Section 3) are highlighted with a purple background. *N* = 15,866 measures from 3194 nestlings for wing length, 12,662 measures from 2887 nestlings for sternum length and 15,968 from 3188 nestlings for body mass.

The function *slidingwin* allows the variation in the start and duration of windows using daily increments and then compares the linear and quadratic relationships of a meteorological variable for a given time window. We looked for windows of all possible lengths, start and end dates, between the reference date (relative to each individual and corresponding to the date of phenotypic measurement at day 50 in the nestling's life) and 50 days before (corresponding to the date of hatching, see Figure 2 for an illustration of nestling life stages and growth).

Models were ranked using ΔAICc values, defined as the difference in terms of AICc (Akaike information criterion corrected for small sample size) between the baseline model and a model with a weather signal. Using ΔAICc values, we are then able to compare the fit of models with different weather signals (temperature, rain, wind and PC1) with the baseline model. Once the best‐fitting weather variable among the three was chosen, we estimated the best window for which the investigated variable best explains the variation in a measured trait. As possible windows are tested and ranked using ΔAICc values, we obtained the windows by averaging the start and end dates of the best models (ΔAICc <2). As spurious results can arise from multiple comparisons, we used the function *randwin* in the package *climwin* to run each model on 1000 randomised datasets and then compared the ΔAICc of the observed versus the randomised data (detailed explanation provided in Data S2 and van de Pol et al., 2016). The model was retained only if the probability of that observed signal was due to change was lower than .01.

Finally, to assess the impact of weather on changes in nestling size at 50 days, we fit a model for each trait (wing length, sternum length and mass), including the same variables as the baseline model and weather variables estimated within the critical best‐fit window associated with each trait. In case of a quadratic trend, the relationship between size at 50 days and weather was further tested to investigate the presence of a threshold by using the package *segmented* v.2.0‐3 (Muggeo, 2008). The segmented package works on the original LMM fitted using the *lme* function in the package *nlme* v.3.1‐165 (Pinheiro et al., 2023). As a starting value (psi) for the threshold has to be suggested, we run the analysis with a few different starting values to better evaluate the robustness of the estimate. Complete analyses and results for all three traits are shown in Data S1.

### 2.5 Effect of weather on nestling growth during the critical developmental windows

To investigate the effect of weather on nestling wing and sternum growth, we first extracted growth rates for the sensitive developmental windows of wings and sternum identified in the climwin analyses (Data S2). As growth trajectories were linear in these windows (Figure 2), individual growth rates were calculated as the slope of linear regression models of nestling wing or sternum length in relation to age in days. For wing growth, we restricted our analyses to individuals with at least three measurements during the developmental window of interest (i.e. day 1–48; see Section 3). For sternum growth, the developmental window of interest was narrower (i.e. day 12–34; see Section 3), and thus, we also included individuals with only two measurements. For each individual growth trajectory, we ensured the good fit of our linear approach by checking the *r* ^2^ of our regression line (mean ± SE *r* ^2^ of regressions lines for wing and sternum growth: .991 ± .012 and .997 ± .010; see also the Data S2). We did not test for an effect of weather conditions on body mass growth rate during the window identified because it included only the 2 days preceding the measurement (see Section 3), and we do not have growth measurements over such a short period. In addition, this would not represent growth per se but mainly daily fluctuation in mass due to environmental conditions.

Secondly, to assess the impact of weather on changes in nestling growth, we fit a model for the growth rates of wings and sternum, including the same weather variables estimated within the critical best‐fit window associated with each trait. Linear and non‐linear (quadratic) effects of weather variables were tested and ranked using AICc, and the most parsimonious model was chosen if ΔAICc <2. The variables brood size at hatching, hatching day and colony (Solothurn or Biel) were also included as fixed effects. As random effects, we included brood ID and year as factors to account for non‐independence among nestlings belonging to the same brood and among nestlings hatched in the same year respectively.

### 2.6 Consequences of early‐life weather conditions

We then tested the effect of weather during nestling growth on fledging success. We used two response variables: weighted proportion of nestlings that fledged and age at fledging (in days). The weighted proportion was constructed using the function *cbind* with the two variables: the number of nestlings of the brood that fledged and the number of nestlings that did not fledge, calculated for nests with at least one hatchling. The weighted proportion was modelled with a GLMM with a binomial family, and the age at fledging was modelled with an LMM. Overdispersion in the binomial model was checked using the function *check_overdispersion* in the package *performance* v.0.10.9 (Lüdecke et al., 2021) and fitted in the model with an observation‐level random effect (Elston et al., 2001). As a weather variable, we tested the three weather variables relative to the sensitive window of each morphological trait. Models were then ranked using AICc, and the best‐fitting model was used. If models had a similar fit (ΔAICc <2), the weather variable with the broadest window was chosen. All models included brood size at hatching, hatching day and colony (Solothurn or Biel) as fixed effects. As random effects, we included brood ID (except for the weighted proportion of fledged nestlings) and year as a factor to account for non‐independence among nestlings belonging to the same brood and nestlings hatched in the same year respectively. For the analysis of the proportion of fledged nestlings, we used the full dataset, whereas age at fledging was available only for the first 11 years of the study.

## 3 RESULTS

Sample sizes and variations in nestling wing and sternum length and body mass at 50 days after hatching are presented in Table 1. The coefficient of variation was highest for body mass (10.0%), followed by wing length (5.6%) and sternum length (3.6%). In contrast, the difference in average size between nestling and adult size was the highest for wing length (6.3%), followed by body mass (4.2%) and sternum length (2.2%).

**TABLE 1:** Means and standard deviations (SD), ranges (min and max), coefficients of variation (CV) and sample sizes in wing length, sternum length and body mass of 50‐days‐old nestling Alpine swifts measured between 1999 and 2023 in two Swiss colonies (Biel and Solothurn) and of adult birds measured in the same colonies and the same study period.

Trait	Nestlings at 50 days of age						Adults					
	Mean ± SD	Range (min; max)	CV (%)	Sample sizes			Mean ± SD	Range (min; max)	CV (%)	Sample sizes		
				*N* nestlings	*N* broods	*N* years				*N* measures	*N* adults	*N* years
Wing length (mm)	212.3 ± 11.8	151.0; 237.0	5.6	2693	1307	25	226.9 ± 4.5	210.5; 244	2.0	5130	1506	25
Sternum length (mm)	39.5 ± 1.4	31.5; 44.0	3.6	2447	1191	22	40.4 ± 1.2	36.0; 44.0	3.1	4203	1363	22
Body mass (g)	94.9 ± 9.5	56.3; 127.3	10.0	2693	1307	25	98.4 ± 7.6	63.0; 126.1	7.9	6795	1507	25

### 3.1 Effect of weather on nestling phenotype at day 50

**FIGURE 3:**
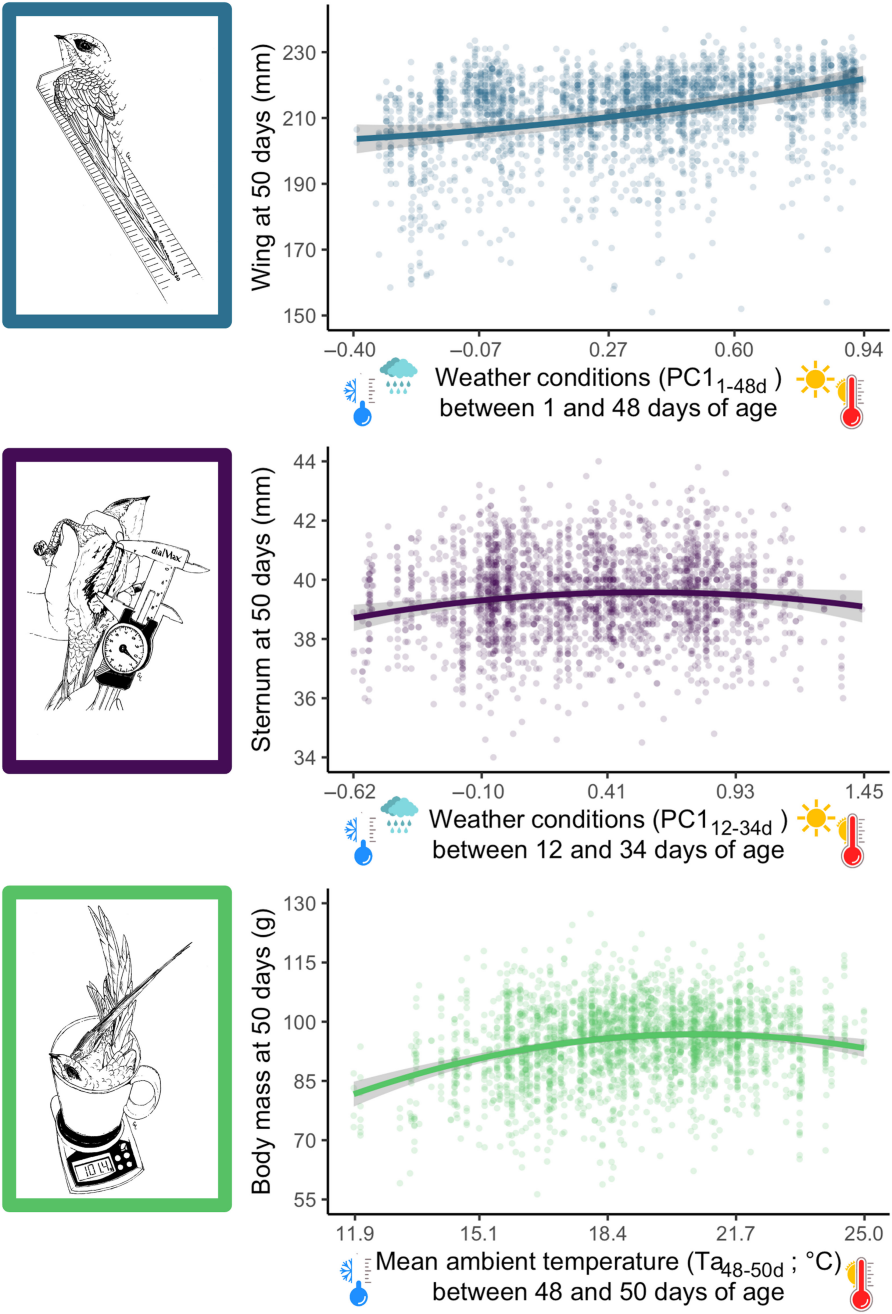
Variation in wing length, sternum length and body mass of 50‐day‐old nestling Alpine swifts in relation to weather conditions encountered earlier in their development. Weather conditions resulted from a principal component analysis (PC1) between daily average temperature and daily rain, and traits were affected by weather conditions over different time windows, that is, PC1_1‐48d_ between 1 and 48 days for wing length, PC1_12‐34d_ between 12 and 34 days for sternum length and mean ambient temperature between 48 and 50 days for body mass (Ta_48‐50_). Low PC1 values indicate cold and rainy days and high values indicate sunny and warm days. Solid lines (and 95% confidence intervals) are predictions from the models presented in Table 2. Climatic windows are reported using the nestling's age as a reference.

**FIGURE 4:**
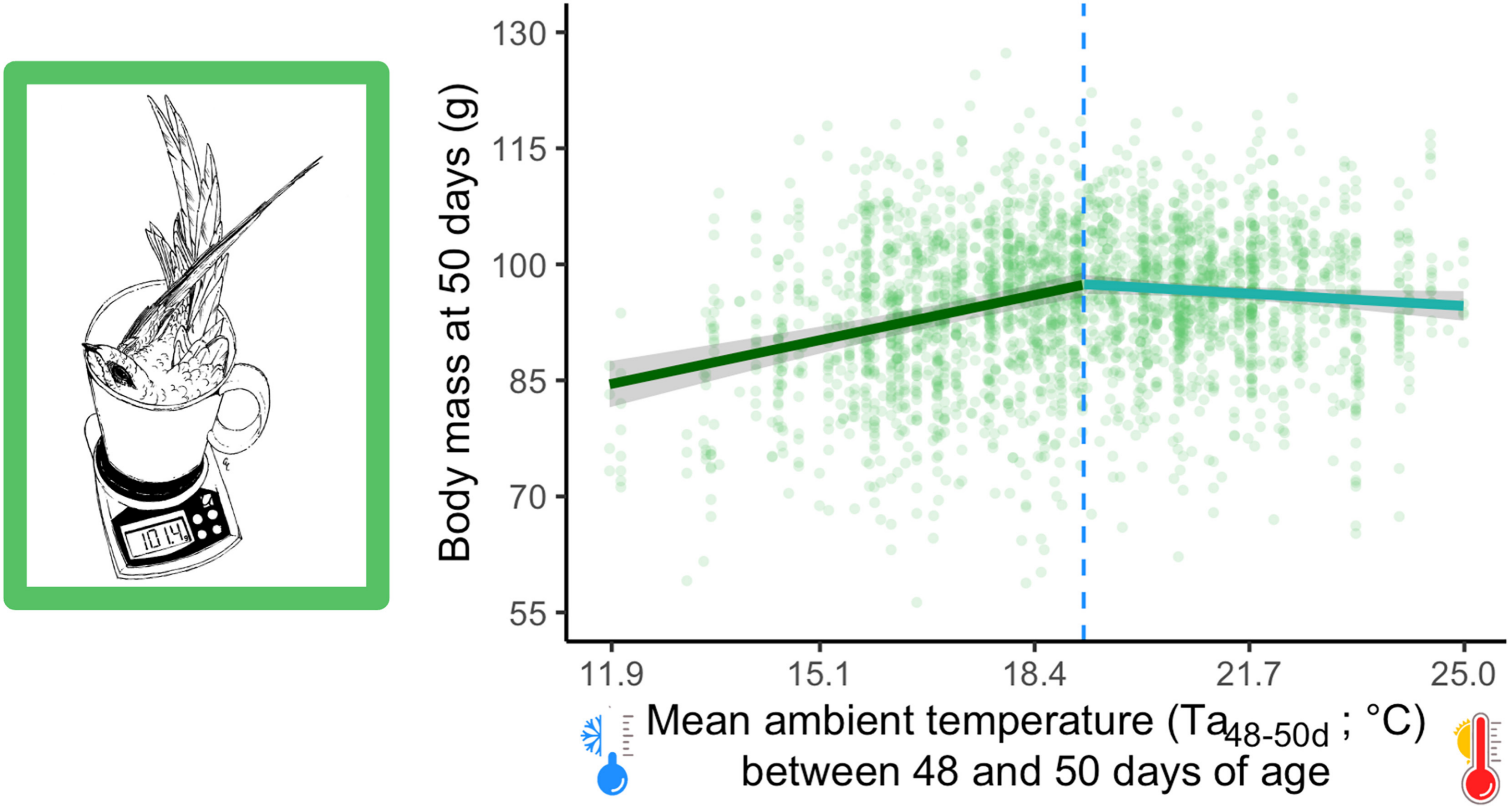
Variation in body mass of 50‐day‐old nestling Alpine swifts in relation to daily mean ambient temperature in the 2 days before the measurement (Ta_48‐50d_). The analysis using the segmented package identified a threshold at 19.2°C. Solid lines (and 95% confidence intervals) are predictions from the models presented in Data S1. Climatic windows are reported using the nestling's age as a reference.

Analyses using the R package *climwin* (van de Pol et al., 2016) show that the first principal component (PC1) on rain and temperature data was the weather variable best explaining the variation in nestling phenotype at day 50 for wings and sternum (Data S1), whereas for body mass it was the mean ambient temperature. The effect on nestling size of variables not in the best model for a specific trait (e.g. wind speed for all traits) is not discussed in the article (Data S1). However, the time windows during which weather conditions affected wing and sternum size and body mass at 50 days differed greatly between traits. Wing length was affected by weather conditions between 1 and 48 days of age, sternum length by weather conditions between 12 and 34 days of age and body mass by the mean ambient temperature between 48 and 50 days of age. Higher PC1 values (indicating warmer and drier weather conditions) in the relevant time windows for a given trait were associated with longer wings and sternum (Table 2; Figure 3). Higher temperature in the relevant window was associated with higher body mass. The effects of weather conditions on nestling sternum length and body mass were, however, non‐linear (significant effects of *weather condition* in Table 2). Follow‐up analyses using the R package *segmented* (Muggeo, 2008) to estimate a threshold did not allow the finding of a breaking point for the sternum length, whereas this was possible for the body mass. The analysis identified a breaking point at 19.2°C (Figure 4), before which the mass showed a significant increase (estimate ± SE: 1.81 ± 0.25, *χ* ^2^ = 52.15, *p* < .001) and after which it showed a significant decrease (estimate ± SE: −0.47 ± 0.23, *χ* ^2^ = 4.20, *p* = .040). Complete analyses and results for both traits are shown in Data S1.

**TABLE 2:** Variation in wing length, sternum length and body mass in 50‐day‐old nestling Alpine swifts in relation to weather conditions encountered earlier in their development.

Predictors	Wing 50			Sternum 50			Mass 50		
	Estimate	SE	*p*	Estimate	SE	*p*	Estimate	SE	*p*
Intercept	216.99	1.53	**<.001**	40.23	0.16	**<.001**	18.90	10.92	.083
Weather condition (PC1 or temperature)	10.71	3.07	**<.001**	0.70	0.21	**.001**	8.00	1.15	**<.001**
Weather condition^2^ (PC1^2^ or temperature^2^)	5.48	4.15	.186	−0.62	0.22	**.006**	−0.19	0.03	**<.001**
Brood size at hatching	−4.04	0.34	**<.001**	−0.35	0.05	**<.001**	−2.49	0.32	**<.001**
Colony [Solothurn]	1.38	0.49	**.004**	0.16	0.07	**.022**	2.68	0.46	**<.001**
Hatching day	−3.51	0.33	**<.001**	−0.21	0.04	**<.001**	−1.57	0.28	**<.001**
Age (days)	2.42	0.14	**<.001**	0.09	0.02	**<.001**	0.21	0.13	.124
Variance components									
Residual	63.59			1.52			45.94		
Brood ID	25.18			0.35			30.27		
Year	28.06			0.07			2.97		
Sample sizes									
*N* years	25			22			25		
*N* broods	1307			1191			1307		
*N* nestlings	2693			2447			2693		

*Note*: Weather conditions resulted from a principal component analysis (PC1) between daily average temperature and daily rain, and traits were affected by weather conditions over different time windows, that is, PC1_1‐48d_ between 1 and 48 days for wing length, PC1_12‐34d_ between 12 and 34 days for sternum length and mean ambient temperature between 48 and 50 days for body mass (Ta_48‐50_). Low PC1 values indicate cold and rainy days and high values indicate sunny and warm days. As the weather variables were tested for quadratic trends, these were indicated using superscript 2. In these models, we controlled for brood size at hatching, colony (two‐level factor: Biel vs. Solothurn), hatching day and the exact age of the nestlings at the time of measurement (range: 45–55 days). Statistically significant estimates (*P* < .05) were bolded.

As expected, the size at 50 days for each of the three traits decreased with increasing brood size at hatching and later in the season. Nestlings reared in Solothurn were larger than nestlings from Biel.

### 3.2 Effect of weather on nestling growth

**FIGURE 5:**
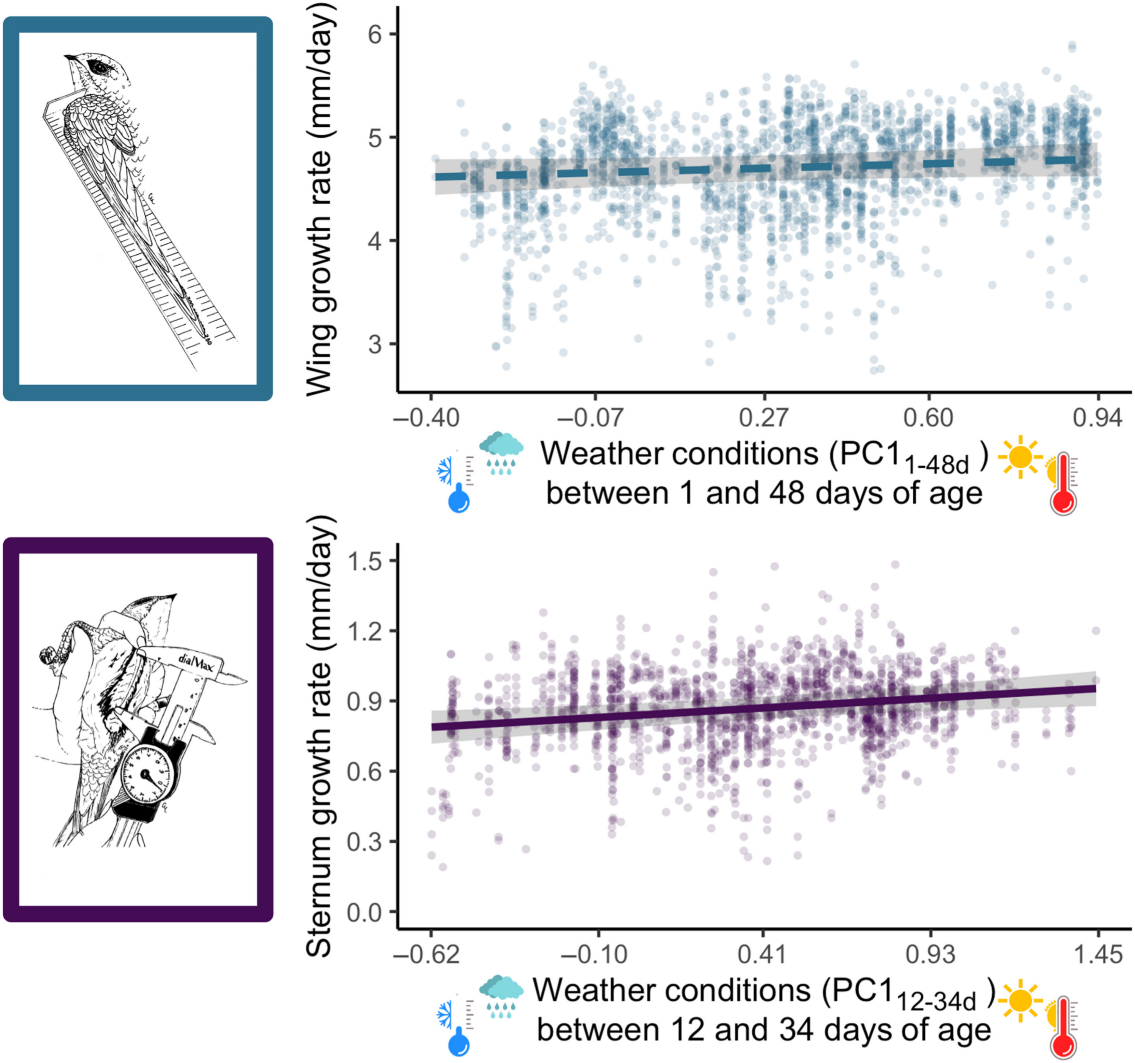
Variation in wing and sternum growth rates of nestling Alpine swifts in relation to weather conditions encountered during development. Weather conditions resulted from a principal component analysis (PC1) between daily average temperature and daily rain, and traits were affected by weather conditions over different time windows. PC1 has low values with cold and rainy days and high values with sunny and warm days. Dashed lines indicate non‐significant relationships. Lines (and 95% confidence intervals) are predictions from the models presented in Table 3. Climatic windows are reported using the nestling's age as a reference.

Weather conditions between 12 and 34 days (i.e. PC1_12‐34d_) had a significant positive effect on the sternum growth rate, with nestlings growing faster on sunny and warm days than on rainy and cold days (Table 3 and Figure 5). Weather conditions (PC1_1‐48d_) had a positive, albeit not statistically significant, effect on wing growth (Table 3). As expected, the growth rates of wings and the sternum decreased with increasing brood size and for nestlings reared later in the breeding season. Nestlings reared in Solothurn grew their wings faster than those in Biel. Sizes at 50 days for both wings and sternum were positively correlated with their respective growth rates (*p* < .001, full results in Data S3).

**TABLE 3:** Variation in wing length, sternum length and body mass in 50‐day‐old nestlings in relation to weather conditions encountered earlier in their development.

Predictors	Wing growth rate			Sternum growth rate		
	Estimate	SE	*p*	Estimate	SE	*p*
Intercept	4.97	0.08	**<.001**	0.91	0.03	**<.001**
Weather condition (PC1)	0.13	0.09	.137	0.08	0.03	**.003**
Brood size at hatching	−0.13	0.01	**<.001**	−0.03	0.01	**<.001**
Colony [Solothurn]	0.08	0.02	**<.001**	−0.00	0.01	.869
Hatching day	−0.14	0.01	**<.001**	−0.02	0.01	**.003**
Variance components						
Residual	0.07			0.01		
Brood ID	0.06			0.01		
Year	0.10			0.01		
Sample sizes						
*N* years	25			21		
*N* broods	1141			893		
*N* nestlings	2344			1824		

*Note*: Weather conditions resulted from a principal component analysis (PC1) between daily average temperature and daily rain, and traits were affected by weather conditions over different time windows, that is, PC1_1‐48d_ between 1 and 48 days for wing length and PC1_12‐34d_ between 12 and 34 days for sternum length. Low PC1 values indicate cold and rainy days and high values indicate sunny and warm days. In these models, we controlled for brood size at hatching, colony (two‐level factor: Biel vs. Solothurn) and hatching day. Statistically significant estimates (*P* < .05) were bolded.

### 3.3 Effect of weather on fledging success

**FIGURE 6:**
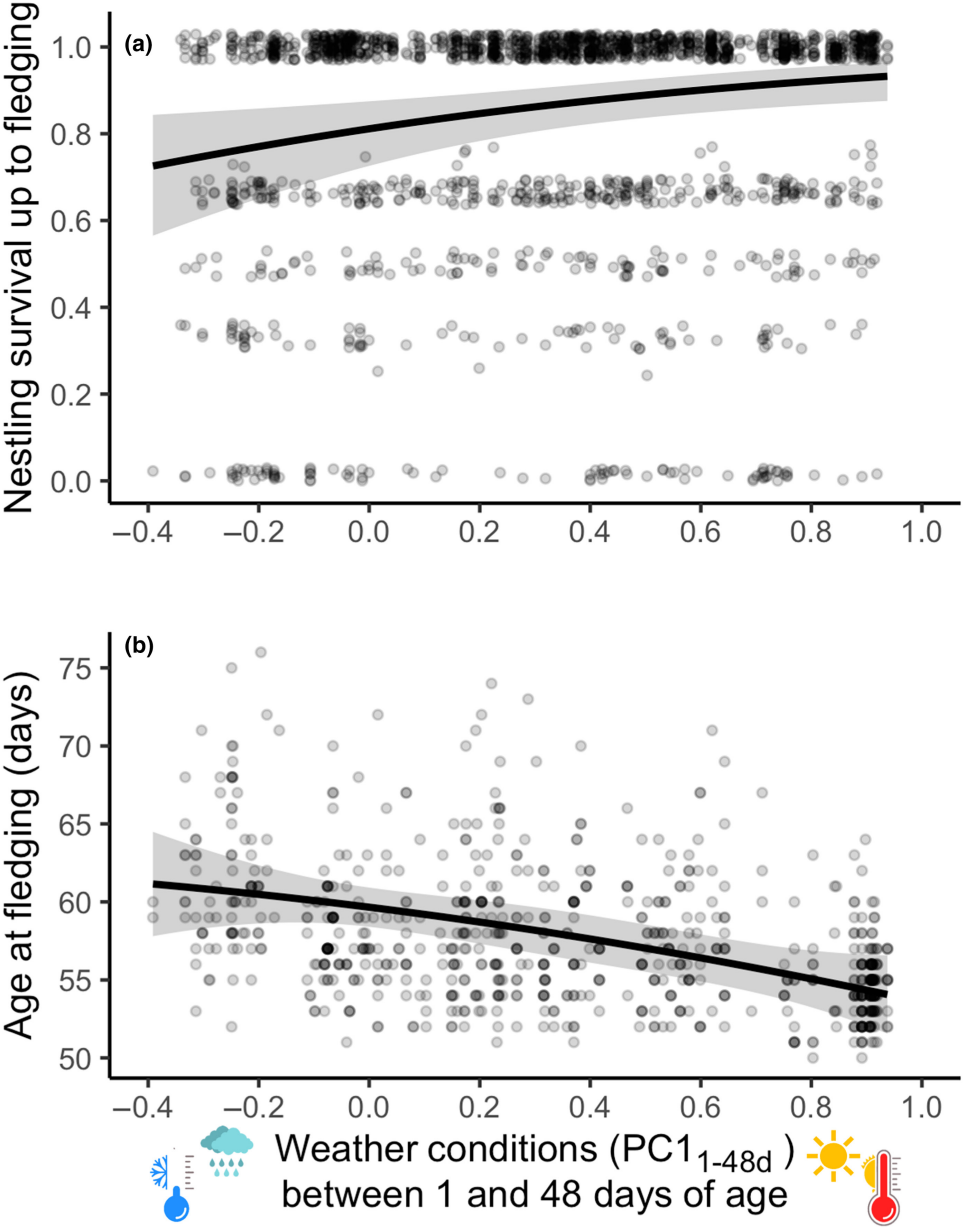
Variation in (a) the nestling survival (calculated as the weighted proportion of nestlings that fledged from a nest with at least one hatchling) and (b) age at fledging (in days) in relation to weather conditions encountered during development in nestling Alpine swifts. Weather conditions are the result of a principal component analysis between daily average temperature and daily rain in the window that best explains the variation in wing length at 50 days. Predicted lines are obtained using average values of the other continuous variables. Climatic windows are reported using the nestling's age as a reference.

Cold and rainy conditions between days 1 and 48 after hatching (i.e. wing‐sensitive developmental period; PC1_1‐48d_) led to a lower proportion of nestlings that survived until fledging (Table 4; Figure 6a) and to delayed fledging of the ones that fledged (Table 4; Figure 6b). The models with weather conditions in the sensitive developmental windows for the sternum (PC1_12‐34d_) and body mass (Ta_48‐50d_) showed higher AICc values compared to the model for the wing‐sensitive developmental window (PC1_1‐48d_; Data S4). Because these developmental windows (PC1_12‐34d_ for sternum and Ta_48‐50d_ for body mass) were shorter than the wing‐sensitive developmental window (PC1_1‐48d_), they were not retained in our final analyses (model selection process in Data S4). Quadratic effects of the weather conditions (PC1_1‐48d_) were also tested but did not significantly improve the models in terms of AICc. Nestlings' survival probability and age at fledgling increased with brood size and later in the season.

**TABLE 4:** Nestling survival up to fledging and age at fledging (in days) in relation to weather conditions between day 1 and 48 after hatching (PC1_1‐48d_), brood size at hatching, colony (Biel and Solothurn) and hatching day (day of the year).

Predictors	Nestling survival up to fledging			Age at fledging		
	Estimate	SE	*p*	Estimate	SE	*p*
Intercept	10.48	3.65	**<.001**	55.80	0.86	**<.001**
Weather condition (PC1)	3.46	1.47	**.003**	−5.53	1.22	**<.001**
Brood size at hatching	0.63	0.06	**<.001**	1.58	0.23	**<.001**
Colony [Solothurn]	1.49	0.17	**<.001**	−0.31	0.50	.538
Hatching day	0.64	0.07	**<.001**	0.99	0.29	**.001**
Variance components						
Residual	4.46			7.51		
Brood ID				6.44		
Obs	1.17					
Year	0.97			2.26		
Sample sizes						
*N* years	25			11		
*N* broods	1787			373		
*N* nestlings				728		

*Note*: Weather conditions result from a principal component analysis between daily average temperature and daily rain during the developmental window that best explained variation in nestling wing length at 50 days. Nestling survival up to fledging is calculated as the weighted proportion of nestlings that fledged from a nest with at least one hatchling. As random effects, we included brood ID (except for the weighted proportion of fledged nestlings) and year as a factor to account for non‐independence among nestlings belonging to the same brood and nestlings hatched in the same year respectively. For the weighted proportion of fledged nestlings, an observation‐level random effect (obs) was included to deal with overdispersion. Statistically significant estimates (*P* < .05) were bolded.

## 4 DISCUSSION

We found that nestling Alpine swifts that had experienced cold and rainy conditions during their growth had shorter wings, a smaller sternum and lower body mass at 50 days after hatching than those that had experienced milder weather. The initial increase in body mass with temperature was followed by a decrease in hotter days. Cold and rainy conditions also lead to higher nestling mortality and delayed fledging. Our results are in agreement with previous studies in birds showing that nestlings often present a reduced size and mass when reared in cold temperatures (e.g. Dawson et al., 2005; de Zwaan et al., 2020; Shipley et al., 2022; but see Andrew et al., 2017) and rainy weather (e.g. Morganti et al., 2017; Pipoly et al., 2013; Siikamäki, 1996), as well as higher mortality (e.g. Shipley et al., 2022). Previous results on common swifts (*Apus apus*) show similar negative effects of cold and rainy days documented in northern European populations (Finch et al., 2023; Rajchard et al., 2006; Thomson et al., 1996), whereas, in a southern European population, light rain and colder days had positive effects (Sicurella et al., 2015). These contrasting results indicate that the effects of meteorological conditions, such as daily rainfall and temperature, on a given bird species can vary from one climatic region to another, for example, with cold, rainy days having negative effects in temperate regions and positive effects in Mediterranean regions where it rarely rains or cools down during the breeding season.

Effects of weather conditions on nestling growth and survival can be explained by their consequences on their thermal budget and food availability (Sauve et al., 2021). In temperate regions, nestlings need to increase their investment in heat production on cold days, which rainfall can exacerbate if their plumage is wet, increasing heat loss (Nye, 1964). Cold and rainy weather can also greatly reduce food supply, especially for insectivorous species such as swifts, which feed exclusively on prey caught in flight. Previous studies have observed a significant reduction in aerial insect activity and availability when weather conditions are wetter and/or colder (Garrett et al., 2022; Grüebler et al., 2008; Taylor, 1963; Williams & Buxton, 1951; Winkler et al., 2013). Under these conditions, aerial insectivores may be subject first to locally reduced aerial insect availability and then, if conditions persist, to a lower overall aerial insect abundance. Adverse weather conditions on a localised scale (temporal and geographic) can potentially drastically reduce short‐term food availability, even if overall food abundance is unaffected (Cox et al., 2019). In response to these localised adverse weather events, parents may travel further away from the breeding area to forage and minimise the effects on the growth and survival of their nestlings, at an additional energetic cost to them. However, if these unfavourable weather conditions persist throughout the breeding season, this can lead to a reduction in the abundance of flying insects over large temporal and geographical scales, and ultimately to reduced growth and increased mortality of their nestlings. Our study population of Alpine swifts is in Switzerland, where the climate is temperate, and the colonies are located under the roof of buildings where nestlings are sheltered from rainfall. Therefore, the detrimental effects of temperature and rain on nestling survival and phenotype at day 50 are likely explained by both a combination of effects of weather on food provisioning by their parents and on their thermal budget, with this latter effect potentially being even stronger in other breeding sites and populations where nestlings might be more exposed to rainfall (e.g. cliffs) and wet their down feathers.

The sensitive developmental windows during which the wings, sternum and body mass of Alpine swift nestlings were affected by weather conditions were different for these three traits. Variation in wing length at 50 days was best explained by daily rainfall and temperature experienced between 1 and 48 days of age, sternum by daily rainfall and temperature between 12 and 34 days of age and body mass by daily temperature during the 2 days prior to weighing the nestlings. The sensitive developmental windows for the wings and sternum, but not for the body mass, corresponded to their periods of their linear growth (Figure 2). As changes in the size of wings and sternum in 50‐day‐old nestlings integrate long developmental periods, they are likely caused by a reduction in overall insect abundance over a wide spatial and temporal area (i.e. years with low food availability), whereas body mass was affected on short‐time scale, likely caused by short‐term variation in insect availability. Hence, it suggests that measures of feather growth and, to some extent, skeletal growth best capture the consequences of adverse weather conditions on food availability throughout the whole development of offspring, while body mass better reflects the short, instantaneous effects of weather conditions on their body reserves. Similar immediate effects of weather on body mass in adulthood have been recently reported in this Alpine swift population (Dumas et al., 2024). A higher hierarchy of protection and compensation for body mass over wing and sternum in response to short periods of low food accessibility has already been reported in Bize et al. (2006, see also Bize et al., 2003 for effects of another stressor, ectoparasite load, on wing length rather than body mass in nestling Alpine swifts). The need to compensate for a slower wing growth is likely the main factor explaining the delayed fledging in rainy and cold conditions (this study), or in response to ectoparasite load (Bize et al., 2003). Indeed, delayed fledging in response to poor environmental conditions can allow nestlings to leave their nest at optimal size and mass, which is a common finding in birds (Aldredge, 2016). Delayed fledging can, however, lead to an increase in the probability of nest predation (de Zwaan et al., 2022; Remeŝ & Martin, 2002). The hierarchy of protection and compensation of traits may, therefore, differ between stressors, as the ability to fledge early should be prioritised in response to predation. Consistent with this, species at high risk of nest predation have evolved developmental strategies prioritising skeletal and wing growth over mass (Callan et al., 2019; Cheng & Martin, 2012; Merrill & Grindstaff, 2018). Because Alpine swifts breed in an environment where nest predation is rare, slower growth and delayed fledging is probably the optimal strategy for adapting to adverse weather conditions.

Interestingly, the effect of ambient temperature on nestling body mass was not linear, with a sharp drop in body mass on cold days and a drop, albeit moderate, on hot days. Our population breeds under the roofs of tall buildings, which often lack good thermal insulation. Temperatures under the roof near the nests are, therefore, often much higher than the ambient outdoor temperatures used in our analyses. Swifts, like many other cavity‐nesting species (e.g. Corregidor‐Castro et al., 2023), are likely to experience fitness costs due to heat stress. Our result regarding the decrease in body mass at higher temperatures suggests that there might be an upper limit to the temperature nestling Alpine swifts can withstand under the roofs of our colonies before showing signs of thermal stress. Because cavity‐nesting species face increasing pressure from heat stress in response to global warming, this may favour faster nestling growth and early departure from the nest. Consistent with this, nestling swifts are well known to jump out of their nests on very hot days, even before they are ready to fly, often ending in rescue centres (Pers. Obs.).

Understanding variation in the growth trajectories of different body traits in response to weather conditions and their consequences for survival up to fledging can help us better understand individual responses to climate change and the consequences on population dynamics. The long‐term effects of early‐life weather conditions remain, however, understudied, although we can expect adverse early‐life weather conditions to potentially influence the thermal tolerance of the same individuals in adulthood (Nord & Giroud, 2020), as well as reproductive success and longevity (Briga et al., 2017; Tschirren et al., 2009). With climate change, temperatures are set to continue rising around the world. Research is therefore needed to better understand the effects of climate change throughout the lifetime of an individual, from the early‐life growing conditions to future fitness.

## AUTHOR CONTRIBUTIONS

**Giulia Masoero:** Conceptualization (equal); data curation (supporting); formal analysis (lead); funding acquisition (equal); investigation (equal); methodology (equal); project administration (supporting); visualization (lead); writing – original draft (lead); writing – review and editing (equal). **Michela N. Dumas:** Conceptualization (supporting); formal analysis (supporting); funding acquisition (equal); investigation (supporting); methodology (supporting); writing – original draft (supporting); writing – review and editing (equal). **Julien G. A. Martin:** Investigation (supporting); writing – review and editing (supporting). **Pierre Bize:** Conceptualization (lead); data curation (lead); formal analysis (lead); funding acquisition (lead); investigation (lead); methodology (supporting); project administration (lead); resources (lead); supervision (lead); validation (equal); visualization (supporting); writing – review and editing (supporting).

## FUNDING INFORMATION

This project has received funding from the European Union's Horizon 2020 research and innovation programme under grant agreement No 101025938 to GM. MD was supported by an Ontario Graduate Scholarship. JGAM was supported by the Natural Sciences and Engineering Research Council of Canada discovery grant (DGECR‐2019‐00289, RGPIN‐2019‐05000) and a University of Ottawa research grant. PB was supported over the years by grants from the Swiss National Science Foundation (PA00A‐109009, 31003A_124988), as well as private funds to support this monitoring.

### OPEN RESEARCH BADGES

This article has earned Open Data and Open Materials badges. Data and materials are available at [[insert provided URL(s) on the Open Research Disclosure Form]].

## Supporting information


Data S1–S4.

## Data Availability

All data and code used for statistical analysis and plots were shared with the editor and reviewers at the first submission and are provided via the Open Science Framework at https://osf.io/2ndmk/ (DOI: https://doi.org/10.17605/OSF.IO/2NDMK).
