# Supplementary material for: Trait‐specific sensitive developmental windows: Wing growth best integrates weather conditions encountered throughout the development of nestling Alpine swifts
Source: Ecol Evol. 2024 Jun 6;14(6):e11491. doi: 10.1002/ece3.11491 (PMC11156578; doi:10.1002/ece3.11491)
Supplement: Supplementary file 1 — Data S1–S4. [file ECE3-14-e11491-s001.pdf]

Trait-specific sensitive developmental windows: wing growth best integrates weather conditions encountered throughout the development of nestling Alpine swifts

Supporting Information and codes for the manuscript

Last compiled on 20 May 2024

Contents

Authors' list and affiliations **2**

ORCIDs . . . 2

Abstract and keywords **2**

Libraries and datasets **3**

Libraries . . . 3

Reproducible environment . . . 3

The datasets . . . 4

Growth curves **7**

Supporting Information 01 - Climwin analyses **11**

Weather data . . . 11

PCA results . . . 12

The climwin approach . . . 13

Base model . . . 13

Finding the best window . . . 14

Investigating the models . . . 15

Wing length at 50 days . . . 15

Sternum length at 50 days . . . 23

Body mass at 50 days . . . 31

Adding the variables to the dataset . . . 41

Figure . . . 42

Testing for segments . . . 43

Sternum length . . . 45

Body mass . . . 49

Supporting Information 02: Growth rates **59**
Checking the resulting growth rates . . . 61
Growth rate models . . . 62
Wing length . . . 62
Sternum length . . . 63
Figure . . . 66

Supporting Information 03: Correlation between the size and growth **66**
Figure . . . 66
Pearson's correlation tests . . . 68

Supporting Information 04: Short-term consequences **69**
Probability of fledging . . . 69
Age at fledging . . . 72
Figure . . . 75
Session information . . . 76

Authors' list and affiliations

Giulia Masoero[1,2] *, Michela Natalina Dumas[2], Julien G.A. Martin[2], Pierre Bize[1] *

[1] Swiss Ornithological Institute, Seerose 1, 6204 Sempach, Switzerland

[2] Department of Biology, University of Ottawa, Canada

* **Corresponding authors**: giulia.masoero@gmail.com, pierre.bize@vogelwarte.ch

ORCIDs

GM: 0000-0003-4429-7726

MND: 0000-0003-2948-8751

JGAM: 0000-0001-7726-6809

PB: 0000-0002-6759-4371

Abstract and keywords

Abstract: The size and growth patterns of nestling birds are key determinants of their survival up to fledging and long-term fitness. However, because traits such as feathers, skeleton and body mass can follow different developmental trajectories, our understanding of the impact of adverse weather on development requires insights on trait-specific sensitive developmental windows. We analysed data from nestling Alpine swifts in Switzerland measured throughout growth up to the age of 50 days (i.e. fledging between 50 and 70 days), for wing length and body mass (2693 nestlings in 25 years) and sternum length (2447 nestlings in 22 years). We show that the sensitive developmental windows for wing and sternum length corresponded to the periods of trait-specific peak growth, which span almost the whole developmental period for wings and

the first half for the sternum. Adverse weather conditions during these periods slowed down growth and reduced size. Although nestling body mass at 50 days showed the greatest inter-individual variation, this was explained by weather in the two days before measurement rather than during peak growth. Interestingly, the relationship between temperature and body mass was not linear, and the initial sharp increase in body mass associated with the increase in temperature was followed by a moderate drop on hot days, likely linked to heat stress. Nestlings experiencing adverse weather conditions during wing growth had lower survival rates up to fledging and fledged at later ages, presumably to compensate for slower wing growth. Overall, our results suggest that measures of feather growth and, to some extent, skeletal growth best capture the consequences of adverse weather conditions throughout the whole development of offspring, while body mass better reflects the short, instantaneous effects of weather conditions on their body reserves (i.e. energy depletion vs storage in unfavourable vs favourable conditions).

Keywords: Apodiformes, feathers, meteorological conditions, climate change, multi-trait, heat stress, offspring development, early-life conditions, *Tachymarptis (Apus) melba*

Journal: Ecology and Evolution

Full script, data and Supporting Information can also be found on the OSF page of the project: https://osf.io/2ndmk/

Libraries and datasets

Libraries

```
# data manipulation
library(dplyr)
library(lubridate)
# models
library(lme4)
library(lmerTest)
library(climwin)
library(segmented)
library(MuMIn)
# graphics
library(ggplot2)
library(ggtext)
library(scales)
# tables
library(sjPlot)
# others
library(performance)
# renv::update(prompt = FALSE) # use to update the packages within the project
# renv::snapshot(prompt = FALSE) # to update the metadata in the renv.lock about the used packages

knitr::opts_chunk$set(
  fig.path = "cache/",
  cache.path = "cache/",
  cache.extra = getRversion()
)
```

Reproducible environment

This project has been initialised using the package *renv*.

```r
citation("renv")
```

```
## To cite package 'renv' in publications use:
##
##   Ushey K, Wickham H (2024). _renv: Project Environments_. R package version 1.0.7,
##   <https://CRAN.R-project.org/package=renv>.
##
## A BibTeX entry for LaTeX users is
##
##   @Manual{,
##     title = {renv: Project Environments},
##     author = {Kevin Ushey and Hadley Wickham},
##     year = {2024},
##     note = {R package version 1.0.7},
##     url = {https://CRAN.R-project.org/package=renv},
##   }
```

This package allows to upload the exact same version of each of the packages used in this project. This is done using the files in the compressed folder "renv.zip". The file has to be decompressed to obtain "renv.lock" file, the ".Rprofile" file and the folder "renv". The packages should then be obtained by running the function renv::restore() in the R console.

For more information https://rstudio.github.io/renv/articles/renv.html

The datasets

Study system

Data were collected between 1999 and 2023 in a Swiss population of Alpine swift. It is a long-distance migratory bird that breeds in colonies of a few to several hundred pairs in holes within cliffs or under the roofs of tall buildings. In Switzerland, Alpine swifts return to their breeding grounds from sub-Saharan Africa at the beginning of April (Meier et al. 2020) and start laying eggs between early May and June, with significant adaptive variations in laying dates depending on weather conditions (de Villemereuil et al. 2020). Females lay one clutch a year, with one to four eggs per clutch (modal clutch size is three). Both parents incubate the eggs for about 18 days and then feed their nestlings until fledging, which occurs around 55 days after hatching (range 50-76 days; (Bize et al. 2004) and this manuscript). After breeding, Alpine swifts migrate back to Africa in September (Meier et al. 2020).

Fieldwork was carried out in two Alpine swift colonies located in clock towers in the Swiss cities of Biel (60-100 breeding pairs) and Solothurn (40-55 breeding pairs), ca. 20km apart. Each year, both colonies were regularly visited to monitor egg laying and clutch size, to capture and measure adults, and to ring and measure nestlings. Nestlings were individually recognised by ringing them with numbered metal rings 10 to 15 days after hatching. Nestlings were measured regularly (usually every 5 to 10 days, on average five times in total) until fledging. At each measurement, we measured wing length with a ruler to the nearest 1 mm, sternum size with a calliper to the nearest 0.1 mm, and body mass with a digital scale to the nearest 0.1 g. The measure of sternum length provides an estimate of skeletal growth and size. Tarsus length has been commonly used in passerines, but it is difficult to measure in a repeatable way in a species with short and bulky tarsi, such as swifts. As nestlings are not ringed at hatching, the age of the nestlings in a brood is based on the hatching date of the first nestling; the last nestling is usually born on the same day or 1 day later. Therefore, measurements for a brood of three nestlings, for example, are taken when the first-hatched nestling reaches 50 days of age, the youngest one might be the same age or 1 day younger. Only nestlings that survived up to fledging were included in the statistical analyses. Sample sizes differ between traits, as wing length and body mass have been measured since 1999, while sternum length has been measured since 2003.

```r
# parameters for colors
par_col_wing_50 <- "#2D708EFF"
par_col_wing_growth <- "#2D708EFF"
par_col_sternum_50 <- "#440154FF"
par_col_sternum_growth <- "#440154FF"
par_col_mass_50 <- "#56C667FF"

# functions from another script
source("script_functions.R")

# loading the datasets
data_clim <- read.csv("data/data_clim.csv",
  stringsAsFactors = TRUE, na = c("", "NA")
)
data_am_nestlings <- read.csv("data/data_nestlings.csv",
  stringsAsFactors = TRUE, na = c("", "NA")
)
data_am_nestlings_all <- read.csv("data/data_nestlings-all-meas.csv",
  stringsAsFactors = TRUE, na = c("", "NA")
)
data_am_nests <- read.csv("data/data_nests.csv",
  stringsAsFactors = TRUE, na = c("", "NA")
)
```

```r
# creating the metadata
data_clim_meta <- data.frame(
  column_name = colnames(data_clim),
  description = c(
    "year of weather recording",
    "date of weather recording",
    "daily mean ambient temperatures (°C) averaged using data from five stations obtained from Swiss met
    "daily total rainfal (degrees Celsius) averaged using data from five stations obtained from Swiss me
    "daily mean wind (degrees Celsius) averaged using data from five stations obtained from Swiss meteo
  )
)
write.csv(data_clim_meta,
  file = "data/data_clim_metadata.csv",
  row.names = FALSE
)

data_am_nestlings_meta <- data.frame(
  column_name = colnames(data_am_nestlings),
  description = c(
    "year of measurement recording",
    "colony where the nest was located",
    "code of the nest, equivalent to broodID",
    "individual ID of the nestling",
    "date of hatching of the first nestling in the brood",
    "date of measurement of the nestling",
    "age in days of the nestling at the time of measurement",
    "hour of measurement of the nestling",
    "mass at day 50",
    "wing length at day 50",
```

```r
    "sternum length at day 50",
    "age at fledging in days since hatching",
    "number of days from the 1st of May of hatching of the first nestling in the brood",
    "brood size (number of hatchlings) of the nest of the focal nestling"
  )
)
write.csv(data_am_nestlings_meta,
  file = "data/data_nestlings_metadata.csv",
  row.names = FALSE
)

data_am_nestlings_all_meta <- data.frame(
  column_name = colnames(data_am_nestlings_all),
  description = c(
    "individual ID of the nestling",
    "wing length at the time of measurement",
    "sternum length  at the time of measurement",
    "mass at the time of measurement",
    "age in days of the nestling at the time of measurement"
  )
)
write.csv(data_am_nestlings_all_meta,
  file = "data/data_nestlings_all-meas_metadata.csv",
  row.names = FALSE
)

data_am_nests_meta <- data.frame(
  column_name = colnames(data_am_nests),
  description = c(
    "colony where the nest was located",
    "year of measurement recording",
    "date of hatching of the first nestling in the brood",
    "number of nestlings in the brood that did not fledge",
    "number of nestlings in the brood that fledged",
    "brood size of the nest (number of hatchlings)"
  )
)

write.csv(data_am_nests_meta,
  file = "data/data_am_nests_metadata.csv",
  row.names = FALSE
)
```

```r
# handling of date variables
data_am_nestlings$date_meas <- as.Date(data_am_nestlings$date_meas)
data_am_nestlings$date_hatch <- as.Date(data_am_nestlings$date_hatch)
data_am_nestlings$date_50days <- data_am_nestlings$date_hatch + 50
data_am_nestlings$hatch_doy <- yday(data_am_nestlings$date_hatch)
data_clim$date <- as.Date(data_clim$dateYMD, "%d/%m/%y")

# scaling variables for the analyses
data_am_nestlings$day_hatch_1may_sc <-
  fun_scale(data_am_nestlings$day_hatch_1may)
```

```r
data_am_nestlings$hatch_doy_sc <- fun_scale(data_am_nestlings$hatch_doy)
# age in days scaled with 50
data_am_nestlings$age_days_sc50 <- data_am_nestlings$age_days - 50

# year of measurement as factor
data_am_nestlings$year_f <- as.factor(data_am_nestlings$year)
data_am_nestlings$ring <- as.factor(data_am_nestlings$ring)
```

Creating the subsets needed for the analyses

```r
data_am_nestlings_wing_50 <- subset(data_am_nestlings, !is.na(wing_50))
data_am_nestlings_sternum_50 <- subset(data_am_nestlings, !is.na(sternum_50))
data_am_nestlings_mass_50 <- subset(
  data_am_nestlings,
  !is.na(mass_50) & !is.na(day_hatch_1may)
)
# To include only the reproductive season: subset clim to 1st of May -> 10th of September
data_clim_sub <- subset(
  data_clim,
  as.numeric(strftime(data_clim$date, "%m")) %in% 5:8 |
    as.numeric(strftime(data_clim$date, "%m")) == 9 &
      as.numeric(strftime(data_clim$date, "%d")) <= 10
)

nestlings <- subset(data_am_nestlings_all, ring %in% data_am_nestlings$ring)

nestlings_n <- nestlings %>%
  group_by(ring) %>%
  mutate(n_measures = sum(!is.na(unique(mass)))) %>%
  slice(which.max(age_days))
```

Growth curves

Developmental trajectories, from hatching to fledging, of nestling Alpine swifts. Dots represent individual measurements of wing and sternum length and body mass of nestlings measured between 1999 and 2023, and smooth lines represent the average growth pattern of nestlings. The sensitive developmental windows during which weather conditions most affected the phenotype at 50 days (see results below) are highlighted with a purple background.

```r
cowplot::plot_grid(
  fun_plot_growth(data_am_nestlings_all, "wing"),
  fun_plot_growth(data_am_nestlings_all, "sternum"),
  fun_plot_growth(data_am_nestlings_all, "mass") +
    xlab("Age (days)"),
  nrow = 3, align = "hv"
)
```

```r
# Save the plot to the figures directory
ggsave("figures/Fig. 1 - Growth curves.png", width = 7, height = 5, dpi = 300)
```

Sample sizes for this figure:

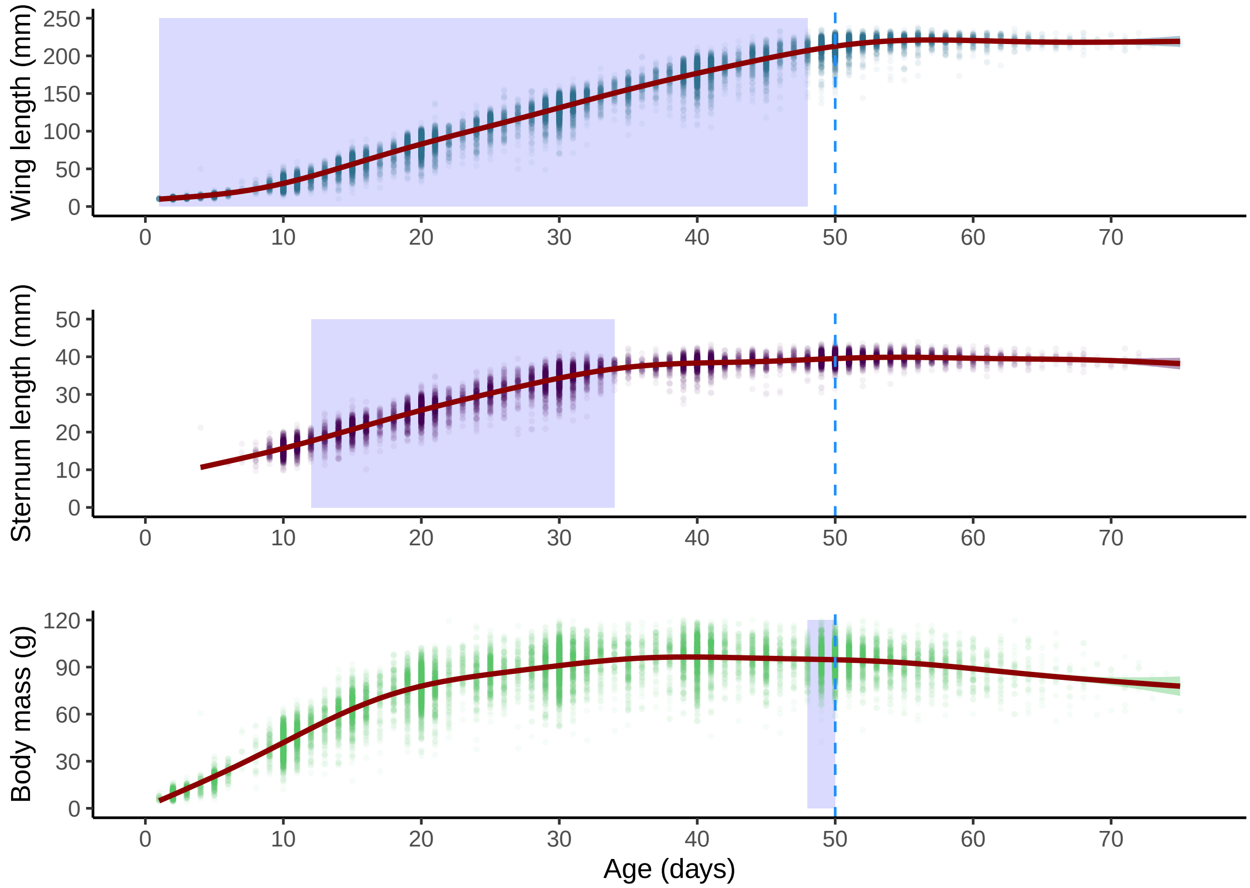

Figure 1: Fig. 1 - Growth curves for the wing, sternum and body mass in Alpine swifts nestlings. The rectangle in blue highlights the time interval used to calculate the growth rates used in the models. The vertical dashed line marks day 50 of chicks growth.

```
table_means <- data.frame(
  Trait = c("Wing", "Sternum", "Body mass"),
  No_measures = c(
    sum(
      !is.na(data_am_nestlings_all$wing)
    ),
    sum(
      !is.na(data_am_nestlings_all$sternum)
    ),
    sum(
      !is.na(data_am_nestlings_all$mass)
    )
  ),
  No_individuals = c(
    sum(
      !is.na(unique(subset(data_am_nestlings_all, !is.na(wing))$wing))
    ),
    sum(
      !is.na(unique(subset(data_am_nestlings_all, !is.na(sternum))$sternum))
    ),
    sum(
      !is.na(unique(subset(data_am_nestlings_all, !is.na(mass))$mass))
    )
  )
)

knitr::kable(table_means,
  caption =
    "Number of measures and of individuals measured for the measures wing length, sternum length and bo
    ",
  digits = 2
)
```

Table 1: Number of measures and of individuals measured for the measures wing length, sternum length and body mass for nestling Alpine swifts.

Trait	No_measures	No_individuals
Wing	15865	557
Sternum	12662	335
Body mass	15967	1153

```
table_means <- data.frame(
  Trait = c("Wing", "Sternum", "Body mass"),
  MeanSD = c(
    paste0(
      round(mean(data_am_nestlings_wing_50$wing_50), 2), " + ",
      round(sd(data_am_nestlings_wing_50$wing_50), 2)
    ),
    paste0(
      round(mean(data_am_nestlings_sternum_50$sternum_50), 2), " + ",
      round(sd(data_am_nestlings_sternum_50$sternum_50), 2)
```

```
    ),
    paste0(
      round(mean(data_am_nestlings_mass_50$mass_50), 2), " + ",
      round(sd(data_am_nestlings_mass_50$mass_50), 2)
    )
  ),
  Range = c(
    paste0(
      min(data_am_nestlings_wing_50$wing_50), " - ",
      max(data_am_nestlings_wing_50$wing_50)
    ),
    paste0(
      min(data_am_nestlings_sternum_50$sternum_50), " - ",
      max(data_am_nestlings_sternum_50$sternum_50)
    ),
    paste0(
      min(data_am_nestlings_mass_50$mass_50), " - ",
      max(data_am_nestlings_mass_50$mass_50)
    )
  ),
  C.V. = c(
    fun_cv(data_am_nestlings_wing_50$wing_50),
    fun_cv(data_am_nestlings_sternum_50$sternum_50),
    fun_cv(data_am_nestlings_mass_50$mass_50)
  ),
  No = c(
    sum(
      !is.na(data_am_nestlings_wing_50$wing_50)
    ),
    sum(
      !is.na(data_am_nestlings_sternum_50$sternum_50)
    ),
    sum(
      !is.na(data_am_nestlings_mass_50$mass_50)
    )
  )
)
```

```
knitr::kable(table_means,
  caption =
    "Mean and standard deviation (SD), range (min and max), coefficient of variation (C.V.) and sample s
    ",
  digits = 2
)
```

Table 2: Mean and standard deviation (SD), range (min and max), coefficient of variation (C.V.) and sample size in wing length, sternum length and body mass for nestling Alpine swifts.

Trait	MeanSD	Range	C.V.	No
Wing	212.46 + 11.85	151 – 237	5.58	2693
Sternum	39.48 + 1.42	30.5 – 44	3.60	2447

Trait	MeanSD	Range	C.V.	No
Body mass	94.81 + 9.51	56.3 – 127.3	10.03	2693

Supporting Information 01 - Climwin analyses

Weather data

To estimate the weather conditions during nestling development, we used meteorological data collected from five Swiss meteorological stations surrounding Biel and Solothurn (Bern-Zollikofen, Cressier, Grenchen, Koppigen, Wynau). Doing so allowed us to cover the whole foraging area of the swifts (up to 30 km in a single foraging trip) (Arn-Willi 1960) and to account for microenvironmental variations (i.e., strong weather events captured by one station only). Daily weather data were averaged across the five stations to obtain two variables: mean daily temperature (average air temperature at 2 m above ground for the whole day), daily precipitation (total rainfall for that day) and wind speed (daily mean of the wind speed scalar in m/s.). We also used a principal component analysis to calculate a daily first component (PC1) between temperature and precipitation for the meteorological data collected during the whole breeding season (May-August). PC1 explained 60% of the total variance in weather data, with factor loadings of 0.71 for the mean temperature and -0.71 for the mean precipitation. A high PC1 value, therefore, indicates warm and dry weather, whereas low values indicate cold and rainy weather.

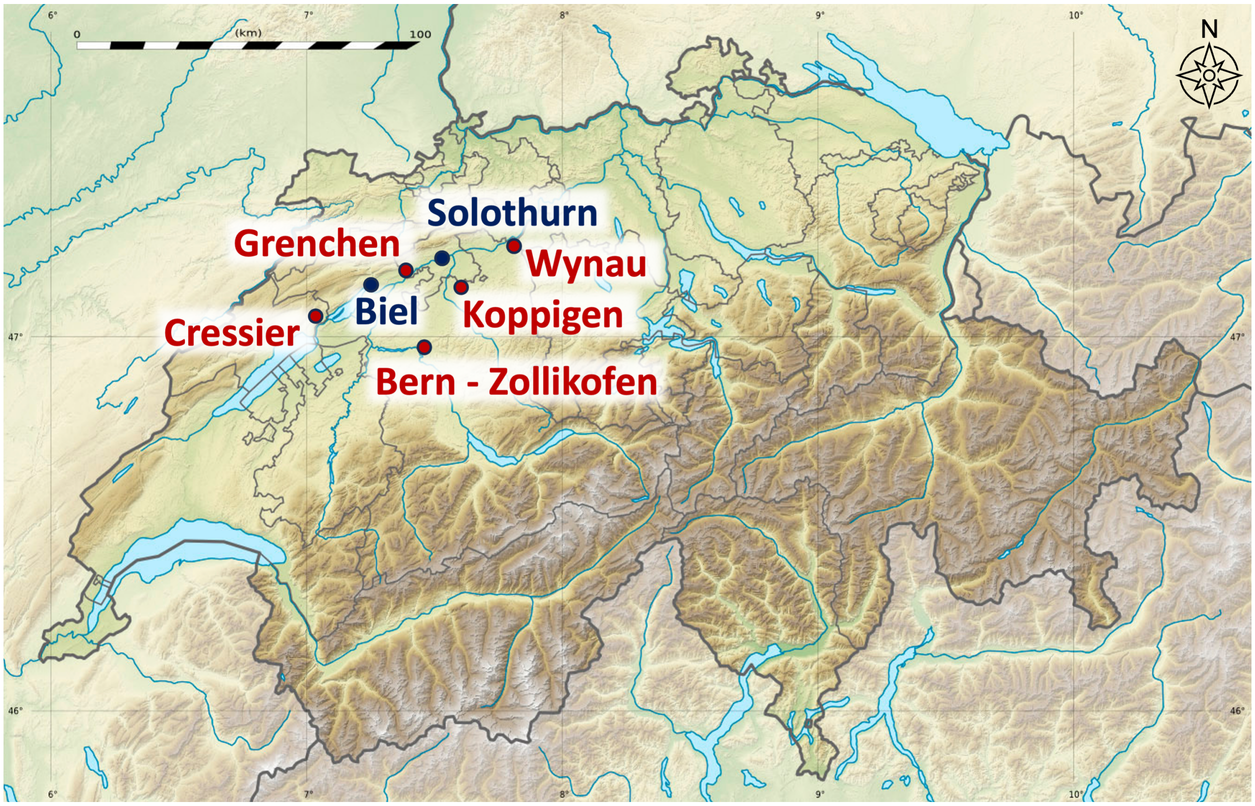

Figure 2: Map of Switzerland representing the location of the two Alpine swift colonies in Biel and Solothurn and the five meteorological stations Bern-Zollikofen, Cressier, Grenchen, Koppigen, Wynau.

[Base map] (https://commons.wikimedia.org/wiki/File:Switzerland_relief_location_map.jpg) credits to Eric Gaba [Wikimedia Commons user: Sting] (https://commons.wikimedia.org/wiki/User:Sting) and

Wikimedia [Commons user: NordNordWest] (https://commons.wikimedia.org/wiki/User:NordNordWest). This file is licensed under the Creative Commons Attribution-Share Alike 3.0 Unported license.

PCA results

A Principal Component Analysis was run between average daily temperature and daily rain.

```
### PCA

# Principal component analyses to create a variable
# with temperature and rain together.
clim_pca1 <- princomp(data_clim_sub[, c("T.daily.mean", "Rain.daily")],
  cor = TRUE, scores = TRUE
)

summary(clim_pca1)
```

```
## Importance of components:
##                           Comp.1    Comp.2
## Standard deviation     1.0879599 0.9035171
## Proportion of Variance 0.5918284 0.4081716
## Cumulative Proportion  0.5918284 1.0000000
```

```
# tr_comp_1 explains 59% of the variance
clim_pca1$loadings
```

```
##
## Loadings:
##              Comp.1 Comp.2
## T.daily.mean  0.707  0.707
## Rain.daily   -0.707  0.707
##
##                Comp.1 Comp.2
## SS loadings       1.0    1.0
## Proportion Var    0.5    0.5
## Cumulative Var    0.5    1.0
```

```
# tr_comp_1 = from cold and rainy to warm and dry day
# adding the new variable to the climate dataset
data_pca1 <- as.data.frame(clim_pca1$scores)
colnames(data_pca1) <- c("tr_comp_1", "tr_comp_2")
data_clim_sub <- cbind(data_clim_sub, data_pca1)
```

The loadings result shows that the Component 1 has positive values with warm days and dry days, and negative values cold and rainy days.

The importance of the components results show that the component 1 explains 59.2% of the variation in the climatic data.

The climwin approach

The overall approach for the climwin analysis is to compare the support by the data for competing hypotheses and to formalize them into regression models (van de Pol et al., 2016).

Competing models are based upon a baseline model (called also null model, a model without weather effects) and ranked using the deltaAICc, or the difference in terms of the Akaike Information Criterion values calculated for a small sample size between the candidate model and baseline model.

Climwin presents the models using the deltaAICc value relative to the baseline model (AICc of the candidate model - AICc of the baseline model). Therefore, a model that is more supported than the baseline model will have a negative deltaAICc value. On the same hand the model with the best support from the data, usually with lowest AICc, will be shown as the model with lowest deltaAICc in the climwin output.

The baseline model was a linear mixed model with the trait (wing length, sternum length or body mass) of the nestlings at 50 days of age in relation to brood size, colony of hatching, hatching day, and exact age of measurement (range 45-55 days of age). The function slidingwin creates a candidate set of competing models testing windows of different lengths for the weather variable of interest, in this study the mean daily ambient temperature (°C).

Non-linear effects of temperature on the traits were taken into account by checking for both linear and quadratic trends. This is mentioned in the climwin output as func = lin (only linear term) func = quad (linear and quadratic terms).

For the analyses we used an individual specific time window, based on the date when the nestlings had 50 days. We then looked for windows between this reference date and 50 days before.

Base model

According to (van de Pol et al. 2016), we built a base model that includes variables that can affect the traits. As random effects, we included brood ID and year as a factor to account for non-independence among nestlings belonging to the same brood and nestlings hatched in the same year, respectively.

Baseline models

```
# double check base models are the same as in script 01

wing_50_basemod <- lme4::lmer(
  wing_50 ~
    brood_size + colony +
    hatch_doy_sc + age_days_sc50 +
    (1 | year_f) + (1 | nestcode_rearing),
  control = lmerControl(optimizer = "bobyqa", optCtrl = list(maxfun = 1e+06)),
  data = data_am_nestlings_wing_50
)

sternum_50_basemod <- lme4::lmer(
  sternum_50 ~
    brood_size + colony +
    age_days_sc50 + hatch_doy_sc +
    (1 | year_f) + (1 | nestcode_rearing),
  control = lmerControl(optimizer = "bobyqa", optCtrl = list(maxfun = 1e+06)),
  data = data_am_nestlings_sternum_50
)

mass_50_basemod <- lme4::lmer(
```

```r
  mass_50 ~
    brood_size + colony +
    hatch_doy_sc + age_days_sc50 +
    (1 | year_f) + (1 | nestcode_rearing),
  control = lmerControl(optimizer = "bobyqa", optCtrl = list(maxfun = 1e+06)),
  data = data_am_nestlings_mass_50
)
```

Finding the best window

Using the function slidingwin allows to search for the best climatic window

```r
# ----------------------
# -------- WING --------

wing_50_sw <- slidingwin(
  baseline = wing_50_basemod,
  xvar = list(
    tr_pc1 = data_clim_sub$tr_comp_1,
    temp_mean = data_clim_sub$T.daily.mean,
    rain_mean = data_clim_sub$Rain.daily,
    wind_mean = data_clim_sub$Wind.daily.mean
  ),
  stat = c("mean"),
  func = c("lin", "quad"),
  type = "relative", # relative to the individual
  range = c(50, 0),
  cinterval = "day",
  cdate = data_clim_sub$date,
  bdate = data_am_nestlings_wing_50$date_50days
)
save(wing_50_sw, file = "output/wing_50_sw.rda")


# -------------------------
# -------- STERNUM --------

sternum_50_sw <- slidingwin(
  baseline = sternum_50_basemod,
  xvar = list(
    tr_pc1 = data_clim_sub$tr_comp_1,
    temp_mean = data_clim_sub$T.daily.mean,
    rain_mean = data_clim_sub$Rain.daily,
    wind_mean = data_clim_sub$Wind.daily.mean
  ),
  stat = c("mean"),
  func = c("lin", "quad"),
  type = "relative",
  range = c(50, 0),
  cinterval = "day",
  cdate = data_clim_sub$date,
  bdate = data_am_nestlings_sternum_50$date_50days
)
```

```r
save(sternum_50_sw, file = "output/sternum_50_sw.rda")

# ----------------------
# -------- MASS --------

mass_50_sw <- slidingwin(
  baseline = mass_50_basemod,
  xvar = list(
    tr_pc1 = data_clim_sub$tr_comp_1,
    temp_mean = data_clim_sub$T.daily.mean,
    rain_mean = data_clim_sub$Rain.daily,
    wind_mean = data_clim_sub$Wind.daily.mean
  ),
  stat = c("mean"),
  func = c("lin", "quad"),
  type = "relative",
  range = c(50, 0),
  cinterval = "day",
  cdate = data_clim_sub$date,
  bdate = data_am_nestlings_mass_50$date_50days
)
save(mass_50_sw, file = "output/mass_50_sw.rda")
```

Investigating the models

Loading the models

```r
model_load <- list.files("output/", pattern = "*.rda", full.names = FALSE)
for (i in seq_len(length(model_load))) {
  assign(model_load[i], load(paste0("output/", model_load[i])))
}
```

Wing length at 50 days The linear+quadratic term for the PC1 better explains the variation in the data (deltaAICc has the lowest value), sorted by deltaAICc such that the best supported model is on top.

```r
wing_50_mod_combos <- (wing_50_sw$combos)

wing_50_mod_combos[order(wing_50_mod_combos$DeltaAICc), ]
```

```
##   response   climate     type stat func DeltaAICc WindowOpen WindowClose
## 5  wing_50    tr_pc1 relative mean quad    -54.89         50           4
## 1  wing_50    tr_pc1 relative mean  lin    -51.78         48           2
## 2  wing_50 temp_mean relative mean  lin    -44.85         44           0
## 6  wing_50 temp_mean relative mean quad    -44.06         46           1
## 3  wing_50 rain_mean relative mean  lin    -35.67         50           4
## 7  wing_50 rain_mean relative mean quad    -33.13         50           4
## 8  wing_50 wind_mean relative mean quad    -30.09         43          16
## 4  wing_50 wind_mean relative mean  lin    -20.50         50          16
```

```r
# to save the row name of the model with the lowest DeltaAICc
n_bestmod_wing_50 <- as.numeric(rownames(
  wing_50_mod_combos[order(wing_50_mod_combos$DeltaAICc), ]
))[1]
```

The best quadratic models The 30 best windows for the linear+quadratic models sorted by deltaAICc. To investigate any other tested hypothesis we can simply replace the number on the left or in the double square brackets with the corresponding list number.

```r
head(wing_50_sw[[n_bestmod_wing_50]]$Dataset, 30)
```

```
##      deltaAICc WindowOpen WindowClose ModelBeta Std.Error ModelBetaQ Std.ErrorQ ModelBetaC
## 1322 -54.89092         50           4  6.551631 0.3361476 11.0387223  0.4863652         NA
## 1223 -54.84205         48           2 12.455726 0.3366066  2.7489047  0.4859876         NA
## 1323 -54.76311         50           3  7.868213 0.3362721  9.3874591  0.4861168         NA
## 1325 -54.45219         50           1  9.825671 0.3363533  7.5729701  0.4851552         NA
## 1324 -54.41977         50           2  9.981766 0.3363880  6.7217760  0.4856579         NA
## 1224 -54.32639         48           1 12.650438 0.3366280  2.9862848  0.4856233         NA
## 1272 -54.08562         49           3  8.544611 0.3364165  8.2142865  0.4861785         NA
## 1222 -54.03660         48           3 10.263727 0.3365971  5.4752835  0.4866291         NA
## 1273 -53.96047         49           2 10.706399 0.3365262  5.4821138  0.4856960         NA
## 1271 -53.87250         49           4  7.190108 0.3363290  9.8540777  0.4864658         NA
## 1274 -53.68233         49           1 10.674798 0.3365185  6.0797486  0.4852664         NA
## 1174 -52.90919         47           2 12.437476 0.3367720  2.1534341  0.4856307         NA
## 1225 -52.84705         48           0 14.456870 0.3368176  0.3027216  0.4858772         NA
## 1221 -52.84061         48           4  8.940424 0.3366223  6.9043294  0.4871025         NA
## 1175 -52.64052         47           1 12.588455 0.3367644  2.5504995  0.4852247         NA
## 1326 -52.30213         50           0 11.662259 0.3366616  4.8728030  0.4856115         NA
## 1126 -51.69699         46           2 12.483527 0.3369035  1.6598150  0.4853873         NA
## 1127 -51.66755         46           1 12.552901 0.3368703  2.2735931  0.4849586         NA
## 1173 -51.55800         47           3 10.411740 0.3368546  4.4021966  0.4863902         NA
## 1275 -51.55052         49           0 12.551106 0.3367897  3.2700689  0.4856945         NA
## 1176 -51.26785         47           0 14.255534 0.3369472  0.1701031  0.4854594         NA
## 1079 -50.76999         45           2 14.147982 0.3371526 -1.1918020  0.4852511         NA
## 1080 -50.35263         45           1 14.108448 0.3371538 -0.4976083  0.4849790         NA
## 1128 -50.35040         46           0 14.079579 0.3370639  0.1735787  0.4851918         NA
## 1125 -50.23049         46           3 10.591392 0.3370209  3.6320887  0.4861570         NA
## 1172 -50.15347         47           4  9.122739 0.3369127  5.6884318  0.4869242         NA
## 1081 -49.36892         45           0 15.294356 0.3372935 -2.0929514  0.4851141         NA
## 1124 -49.10548         46           4  9.264878 0.3370408  5.0356618  0.4866230         NA
## 1078 -48.93918         45           3 12.283747 0.3373236  0.8309290  0.4859783         NA
## 1321 -47.56793         50           5  5.641579 0.3366908 10.7798666  0.4869438         NA
##      ModelInt Function Furthest Closest Statistics     Type K   ModWeight sample.size Randomised
## 1322 217.0848     quad       50       0       mean relative 0 0.084572833          25         no
## 1223 216.9736     quad       50       0       mean relative 0 0.082531069          25         no
## 1323 217.0928     quad       50       0       mean relative 0 0.079337301          25         no
## 1325 216.8889     quad       50       0       mean relative 0 0.067914088          25         no
## 1324 217.0447     quad       50       0       mean relative 0 0.066822304          25         no
## 1224 216.8182     quad       50       0       mean relative 0 0.063774173          25         no
## 1272 217.0634     quad       50       0       mean relative 0 0.056540774          25         no
## 1222 217.0684     quad       50       0       mean relative 0 0.055171766          25         no
## 1273 216.9947     quad       50       0       mean relative 0 0.053111227          25         no
```

```
## 1271 217.0835     quad       50       0       mean relative 0 0.050825589          25          no
## 1274 216.8472     quad       50       0       mean relative 0 0.046215462          25          no
## 1174 217.0212     quad       50       0       mean relative 0 0.031398094          25          no
## 1225 216.8362     quad       50       0       mean relative 0 0.030437574          25          no
## 1221 217.1366     quad       50       0       mean relative 0 0.030339628          25          no
## 1175 216.8444     quad       50       0       mean relative 0 0.027451227          25          no
## 1326 216.9188     quad       50       0       mean relative 0 0.023178328          25          no
## 1126 217.0579     quad       50       0       mean relative 0 0.017126836          25          no
## 1127 216.8582     quad       50       0       mean relative 0 0.016876543          25          no
## 1173 217.1755     quad       50       0       mean relative 0 0.015977034          25          no
## 1275 216.8741     quad       50       0       mean relative 0 0.015917366          25          no
## 1176 216.8405     quad       50       0       mean relative 0 0.013819467          25          no
## 1079 217.0713     quad       50       0       mean relative 0 0.010774101          25          no
## 1080 216.8755     quad       50       0       mean relative 0 0.008744850          25          no
## 1128 216.8407     quad       50       0       mean relative 0 0.008735114          25          no
## 1125 217.2351     quad       50       0       mean relative 0 0.008226783          25          no
## 1172 217.2726     quad       50       0       mean relative 0 0.007915981          25          no
## 1081 216.8539     quad       50       0       mean relative 0 0.005347413          25          no
## 1124 217.3224     quad       50       0       mean relative 0 0.004687464          25          no
## 1078 217.2487     quad       50       0       mean relative 0 0.004313467          25          no
## 1321 217.3428     quad       50       0       mean relative 0 0.002173012          25          no
```

The best windows (deltaAICc difference from the best model < 2) for the linear+quadratic models sorted by deltaAICc. All models with the lowest AICc present very comparable windows:

```
wing_50_bestmod <- subset(
  wing_50_sw[[n_bestmod_wing_50]]$Dataset,
  deltaAICc < wing_50_sw[[n_bestmod_wing_50]]$Dataset$deltaAICc[1] + 2
)

wing_50_bestmod
```

```
##      deltaAICc WindowOpen WindowClose ModelBeta Std.Error ModelBetaQ Std.ErrorQ ModelBetaC
## 1322 -54.89092         50           4  6.551631 0.3361476  11.038722  0.4863652         NA
## 1223 -54.84205         48           2 12.455726 0.3366066   2.748905  0.4859876         NA
## 1323 -54.76311         50           3  7.868213 0.3362721   9.387459  0.4861168         NA
## 1325 -54.45219         50           1  9.825671 0.3363533   7.572970  0.4851552         NA
## 1324 -54.41977         50           2  9.981766 0.3363880   6.721776  0.4856579         NA
## 1224 -54.32639         48           1 12.650438 0.3366280   2.986285  0.4856233         NA
## 1272 -54.08562         49           3  8.544611 0.3364165   8.214286  0.4861785         NA
## 1222 -54.03660         48           3 10.263727 0.3365971   5.475283  0.4866291         NA
## 1273 -53.96047         49           2 10.706399 0.3365262   5.482114  0.4856960         NA
## 1271 -53.87250         49           4  7.190108 0.3363290   9.854078  0.4864658         NA
## 1274 -53.68233         49           1 10.674798 0.3365185   6.079749  0.4852664         NA
## 1174 -52.90919         47           2 12.437476 0.3367720   2.153434  0.4856307         NA
##      ModelInt Function Furthest Closest Statistics     Type K  ModWeight sample.size Randomised
## 1322 217.0848     quad       50       0       mean relative 0 0.08457283          25          no
## 1223 216.9736     quad       50       0       mean relative 0 0.08253107          25          no
## 1323 217.0928     quad       50       0       mean relative 0 0.07933730          25          no
## 1325 216.8889     quad       50       0       mean relative 0 0.06791409          25          no
## 1324 217.0447     quad       50       0       mean relative 0 0.06682230          25          no
## 1224 216.8182     quad       50       0       mean relative 0 0.06377417          25          no
## 1272 217.0634     quad       50       0       mean relative 0 0.05654077          25          no
```

```
## 1222 217.0684     quad       50        0       mean relative 0 0.05517177          25          no
## 1273 216.9947     quad       50        0       mean relative 0 0.05311123          25          no
## 1271 217.0835     quad       50        0       mean relative 0 0.05082559          25          no
## 1274 216.8472     quad       50        0       mean relative 0 0.04621546          25          no
## 1174 217.0212     quad       50        0       mean relative 0 0.03139809          25          no
```

Windows plot It's possible to extract the time windows of all the best supported models (i.e. multi-model inference). This panel shows the opening and closing points of the time windows that were best supported by the data, here those models that made up 95% model confidence set.

```
plotwin(wing_50_sw[[n_bestmod_wing_50]]$Dataset)
```

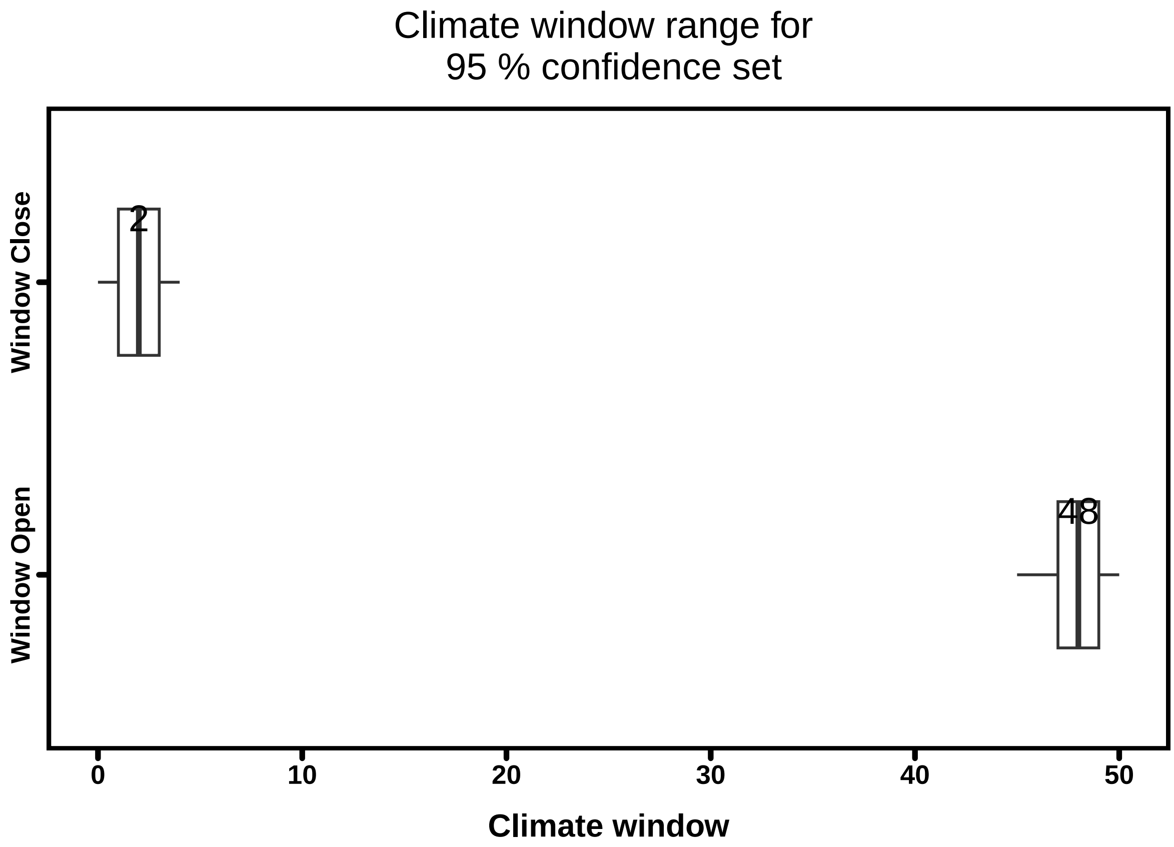

Delta plot The variation in deltaAICc between time windows can be better investigated using the following plot:

```
plotdelta(
  dataset =
    wing_50_sw[[n_bestmod_wing_50]]$Dataset, arrow = TRUE
)
```

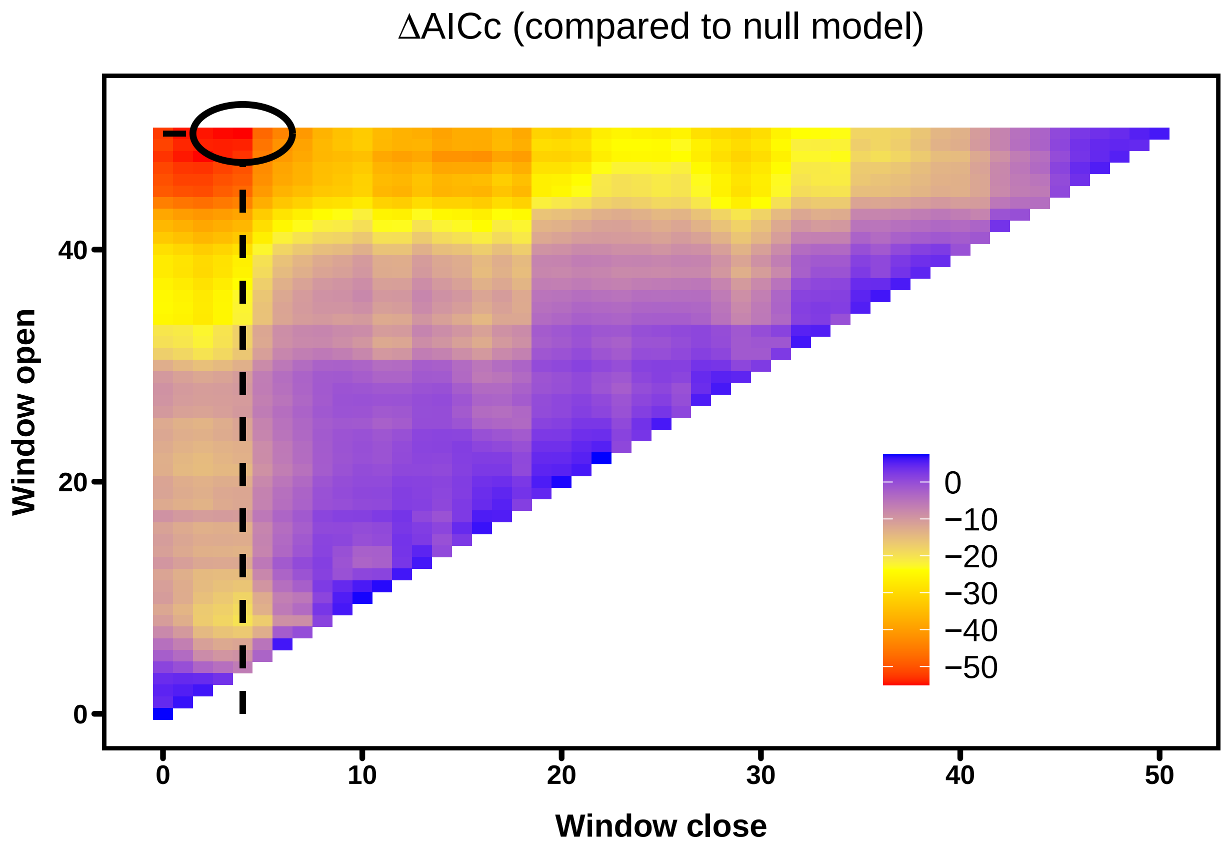

Warmer areas shows values with the lowest deltaAICc (i.e. "best models"). As explained by van de Pol et al., 2016, these deltaAICc landscapes of the different time windows shows multiple peaks (red areas) instead of a clear single peak. This can indicate the presence of multiple (e.g. possibly both long- and short-lag) weather signals within the same weather variable, but it can also occur due to collinearity or chance.

The evidence for multiple signals can be therefore investigated by adding the best supported of the weather windows to the baseline model, and re-fitting all the different time windows again: this tests whether there is still strong model support for the second best (e.g. short-lag) weather window once the other best supported (e.g. long-lag) weather window has been accounted for in the baseline model (here in the Step 2).

Beta plot This panel shows the model support (deltaAICc) for all fitted time windows tried, shown for each combination of Window open (y-axis) and Window close (x-axis). Models with the lowest deltaAICc (red) are the best supported (colours show the deltaAICc levels compared to the null model, see legend). Strongly supported windows will often be grouped together.

```
plotbetas(wing_50_sw[[n_bestmod_wing_50]]$Dataset, arrow = TRUE)
```

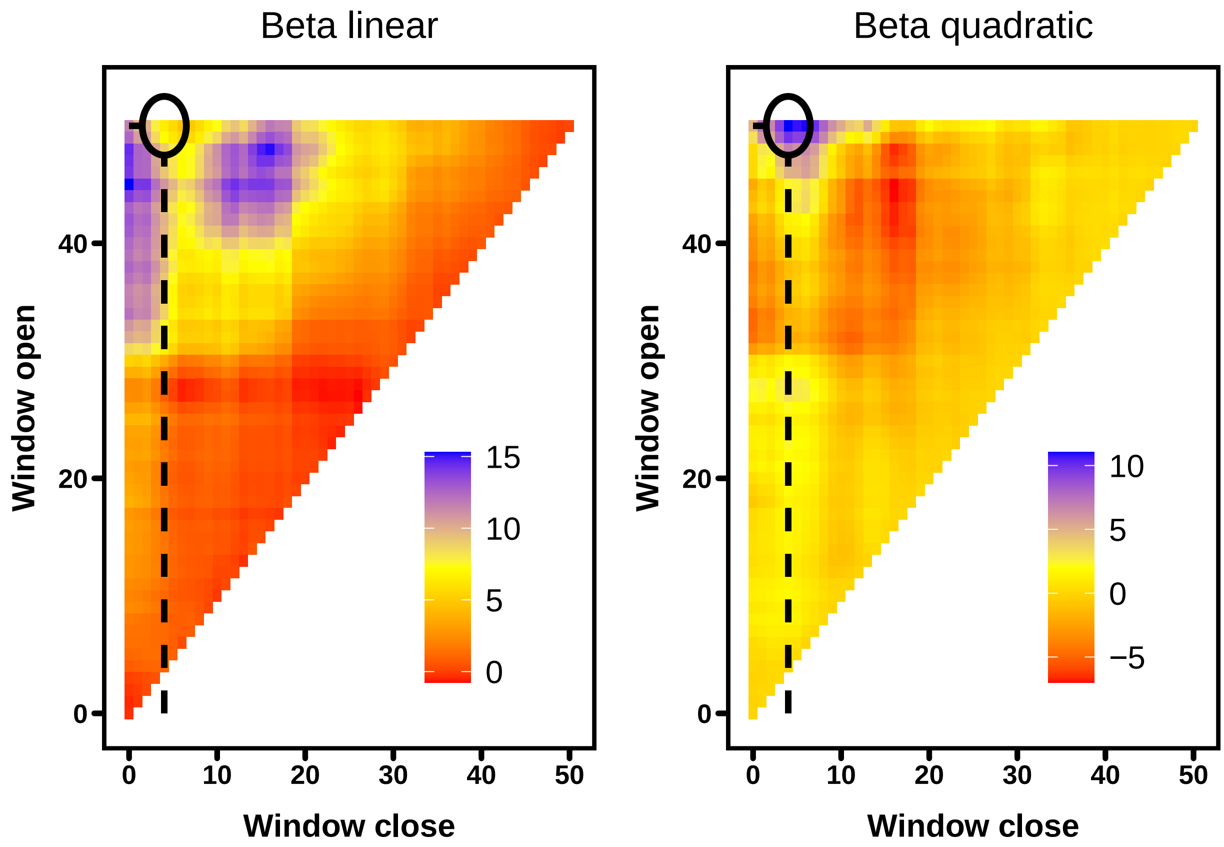

Autocollinearity Correlation between the mean temperature during the best supported time window and the mean temperature over all other time windows.

```
autocoll <- autowin(
  reference = wing_50_sw[[n_bestmod_wing_50]],
  baseline = wing_50_basemod,
  xvar = list(
    tr_pc1 = data_clim_sub$tr_comp_1
  ),
  type = "relative",
  range = c(50, 0),
  stat = "mean",
  cdate = data_clim_sub$date,
  bdate = data_am_nestlings_wing_50$date_50days,
  func = "quad",
  # cmissing = FALSE,
  cinterval = "day"
)

save(autocoll, file = "output/wing_50_autocall.rda")
```

```
load(file = "output/wing_50_autocall.rda")
plotcor(autocoll, type = "A", arrow = TRUE)
```

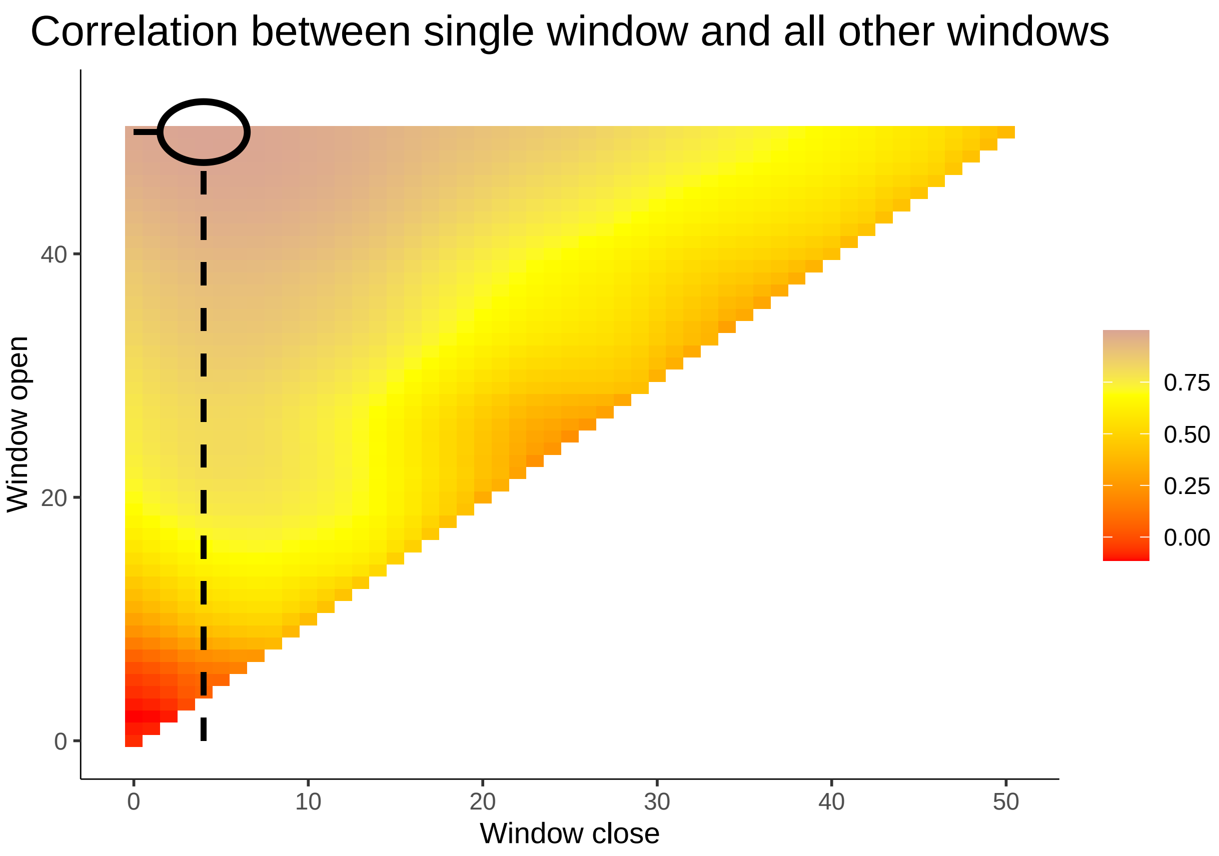

Randwin Using randwin() to randomize the identity of the nestling, we are able to check if the window that was found before is actually important, or the relationship was just random.

```
# Performing randamization to identify
# likelyhood of of dignals occuring by chance

wing_50_rand1000 <- randwin(
  repeats = 1000,
  baseline = wing_50_basemod,
  xvar = list(tr_pc1 = data_clim_sub$tr_comp_1),
  stat = c("mean"),
  func = c("quad"),
  type = "relative", # relative to the individual
  range = c(50, 0),
  cinterval = "day",
  cdate = data_clim_sub$date,
  bdate = data_am_nestlings_wing_50$date_50days,
  cmissing = FALSE
)
save(wing_50_rand1000, file = "output/wing_50_rand1000.rda")
```

```
load("output/wing_50_rand1000.rda")

climwin::pvalue(
  datasetrand = wing_50_rand1000[[1]],
  dataset = wing_50_sw[[n_bestmod_wing_50]]$Dataset,
```

```r
  metric = "AIC",
  sample.size = wing_50_sw[[n_bestmod_wing_50]]$Dataset$sample.size[1]
  # sample size = number of years
)
```

```
## [1] "<0.001"
```

This panel shows a histogram of all deltaAICc values from all models run on the randomized (in grey) and the deltaAICc of the best model fitted on the observed data (dashed line). The best climate window in the randomized data is by definition a false positive, as the randomization procedure removes any climate signal from the data. The percentage of randomizations that generates a deltaAICc value that is at least as low as the deltaAICc value of the best model fitted to the observed data (P value above) is a measure of how likely it is that one obtained the observed deltaAICc by chance and thus whether the signal is likely to be real or not. In our case we can say that the candidate signal is very unlikely to be a false positive: P <0.001.

```r
climwin::plothist(
  datasetrand = wing_50_rand1000[[1]],
  dataset = wing_50_sw[[n_bestmod_wing_50]]$Dataset
)
```

```
## Warning: The dot-dot notation (`..density..`) was deprecated in ggplot2 3.4.0.
## i Please use `after_stat(density)` instead.
## i The deprecated feature was likely used in the climwin package.
##   Please report the issue at <https://github.com/LiamDBailey/climwin/issues>.
## This warning is displayed once every 8 hours.
## Call `lifecycle::last_lifecycle_warnings()` to see where this warning was generated.
```

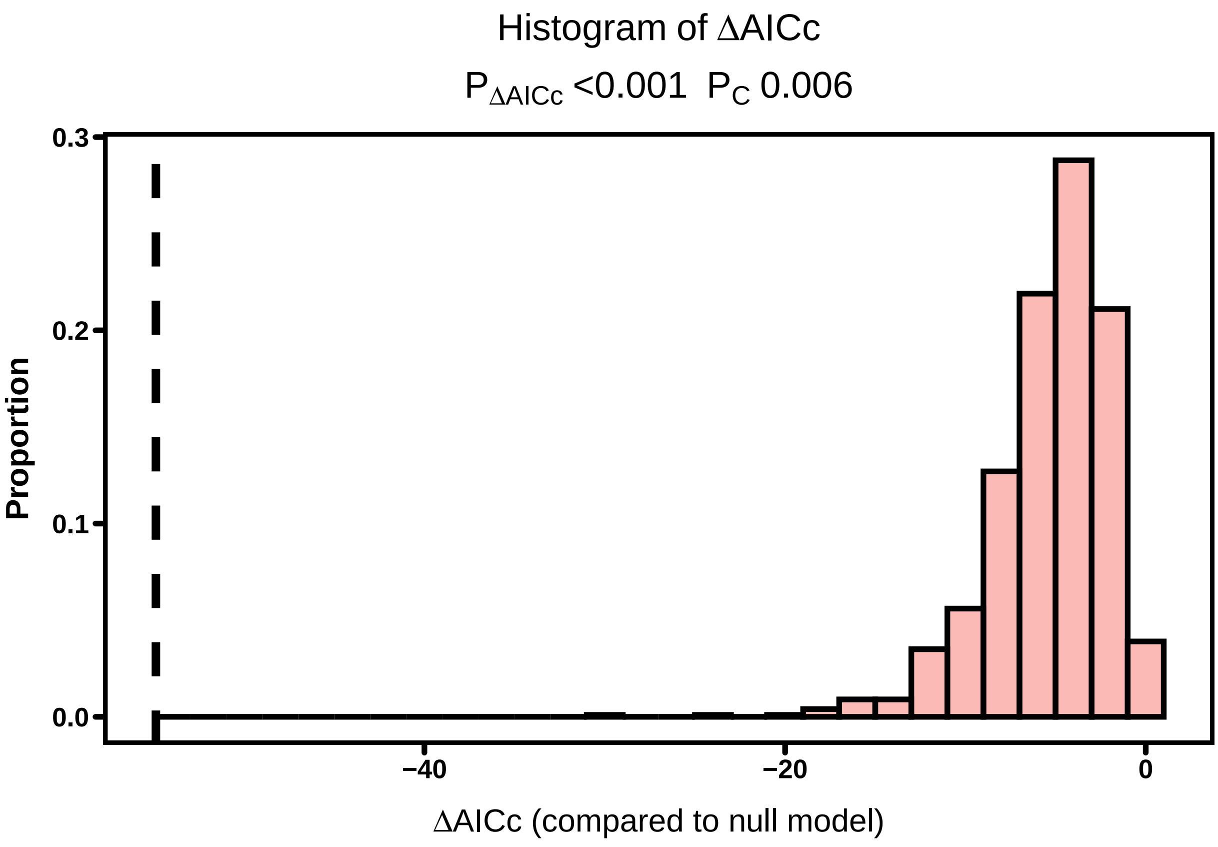

Extracting the best window The best window was obtaining averaging the opening and closing dates for all the best models (deltaAICc < 2). The best window opened 48.92 (mean; SD: 1) and closed 2.33 (mean; SD: 1.07) days before the 50 days measurement, which corresponds to the age of the nestling of 1 and 48 days old.

```
wing_50_bestsw <- singlewin(
  baseline = wing_50_basemod,
  xvar = list(
    tr_pc1 = data_clim_sub$tr_comp_1
  ),
  stat = c("mean"),
  func = c("quad"),
  type = "relative",
  range = c(
    round(mean(wing_50_bestmod$WindowOpen), 0),
    round(mean(wing_50_bestmod$WindowClose), 0)
  ),
  cinterval = "day",
  cdate = data_clim_sub$date,
  bdate = data_am_nestlings_wing_50$date_50days
)
```

```
## fixed-effect model matrix is rank deficient so dropping 2 columns / coefficients
```

```
# adding the climatic variable to the wing_50 dataset
data_am_nestlings_wing_50$signal_for_wing_50 <-
  wing_50_bestsw$BestModelData$climate
```

Sternum length at 50 days The linear+quadratic term for the PC1 better explains the variation in the data (deltaAICc has the lowest value), sorted by deltaAICc such that the best supported model is on top.

```
sternum_50_mod_combos <- (sternum_50_sw$combos)

sternum_50_mod_combos[order(sternum_50_mod_combos$DeltaAICc), ]
```

```
##     response   climate     type stat func DeltaAICc WindowOpen WindowClose
## 5 sternum_50    tr_pc1 relative mean quad     -6.92         31          16
## 1 sternum_50    tr_pc1 relative mean  lin     -5.23         50          16
## 8 sternum_50 wind_mean relative mean quad     -3.74         45          16
## 2 sternum_50 temp_mean relative mean  lin     -2.64         48          14
## 4 sternum_50 wind_mean relative mean  lin     -1.15         45          20
## 3 sternum_50 rain_mean relative mean  lin      0.79         29          16
## 7 sternum_50 rain_mean relative mean quad      3.84         43           2
## 6 sternum_50 temp_mean relative mean quad      4.93         49          14
```

```
n_bestmod_sternum_50 <- as.numeric(rownames(
  sternum_50_mod_combos[order(sternum_50_mod_combos$DeltaAICc), ]
))[1]
```

The best quadratic models The 30 best windows for the linear+quadratic models sorted by deltaAICc. To investigate any other tested hypothesis we can simply replace the number on the left or in the double square brackets with the corresponding list number.

```r
head(sternum_50_sw[[n_bestmod_sternum_50]]$Dataset, 30)
```

```
##      deltaAICc WindowOpen WindowClose ModelBeta  Std.Error ModelBetaQ Std.ErrorQ ModelBetaC
## 512  -6.921814         31          16 0.6439868 0.04890045 -0.4618389 0.06793423         NA
## 513  -6.567573         31          15 0.6923178 0.04888465 -0.4919496 0.06791674         NA
## 545  -6.054905         32          16 0.6681248 0.04894310 -0.4998184 0.06789905         NA
## 514  -5.998077         31          14 0.7277946 0.04889078 -0.5134053 0.06788579         NA
## 546  -5.799299         32          15 0.7153519 0.04891801 -0.5325067 0.06787628         NA
## 974  -5.526967         43          16 0.8627472 0.04896451 -0.7063225 0.06795367         NA
## 1019 -5.476339         44          16 0.8637459 0.04896773 -0.6758509 0.06798146         NA
## 1065 -5.469180         45          16 0.8657824 0.04898187 -0.6529619 0.06801712         NA
## 1066 -5.062738         45          15 0.8729279 0.04899598 -0.6545456 0.06799652         NA
## 930  -5.000570         42          16 0.8194125 0.04897776 -0.6897538 0.06795795         NA
## 511  -5.000428         31          17 0.5647773 0.04895028 -0.4018979 0.06797507         NA
## 1018 -4.955888         44          17 0.8356887 0.04897945 -0.6573560 0.06805801         NA
## 975  -4.940319         43          15 0.8651537 0.04897529 -0.7012805 0.06793445         NA
## 1064 -4.939830         45          17 0.8360772 0.04899397 -0.6333999 0.06809447         NA
## 1020 -4.897758         44          15 0.8637132 0.04898299 -0.6678083 0.06796395         NA
## 547  -4.855831         32          14 0.7328238 0.04892918 -0.5384602 0.06786275         NA
## 526  -4.816052         31           2 0.9740741 0.04896630 -0.8003427 0.06818197         NA
## 1067 -4.797222         45          14 0.8853837 0.04899490 -0.6620802 0.06800663         NA
## 579  -4.759085         33          16 0.6867554 0.04897166 -0.4891356 0.06787970         NA
## 1209 -4.749999         48          16 0.8035990 0.04902974 -0.5286647 0.06819112         NA
## 973  -4.696554         43          17 0.8180629 0.04898258 -0.6667891 0.06803156         NA
## 931  -4.631783         42          15 0.8351901 0.04897879 -0.7008244 0.06793034         NA
## 1063 -4.623354         45          18 0.8130383 0.04899888 -0.6083582 0.06808147         NA
## 1017 -4.572024         44          18 0.8097343 0.04898085 -0.6281466 0.06804767         NA
## 510  -4.517666         31          18 0.5225618 0.04892357 -0.3790670 0.06793934         NA
## 1021 -4.512650         44          14 0.8705341 0.04898466 -0.6666277 0.06796981         NA
## 480  -4.511638         30          16 0.5494362 0.04893859 -0.3708243 0.06810227         NA
## 944  -4.438145         42           2 1.0529507 0.04902264 -1.0107469 0.06804540         NA
## 976  -4.436852         43          14 0.8659776 0.04897874 -0.6932581 0.06793719         NA
## 1210 -4.414423         48          15 0.8045932 0.04903585 -0.5304052 0.06816179         NA
##      ModelInt Function Furthest Closest Statistics     Type K   ModWeight sample.size Randomised
## 512  40.19533     quad       50       0       mean relative 0 0.020872955          22         no
## 513  40.18964     quad       50       0       mean relative 0 0.017484832          22         no
## 545  40.19438     quad       50       0       mean relative 0 0.013531225          22         no
## 514  40.18390     quad       50       0       mean relative 0 0.013152155          22         no
## 546  40.18927     quad       50       0       mean relative 0 0.011907840          22         no
## 974  40.18512     quad       50       0       mean relative 0 0.010391942          22         no
## 1019 40.17877     quad       50       0       mean relative 0 0.010132185          22         no
## 1065 40.17287     quad       50       0       mean relative 0 0.010095982          22         no
## 1066 40.17256     quad       50       0       mean relative 0 0.008239309          22         no
## 930  40.19599     quad       50       0       mean relative 0 0.007987137          22         no
## 511  40.20409     quad       50       0       mean relative 0 0.007986572          22         no
## 1018 40.18291     quad       50       0       mean relative 0 0.007810675          22         no
## 975  40.18407     quad       50       0       mean relative 0 0.007750109          22         no
## 1064 40.17707     quad       50       0       mean relative 0 0.007748212          22         no
## 1020 40.17783     quad       50       0       mean relative 0 0.007586924          22         no
## 547  40.18476     quad       50       0       mean relative 0 0.007429531          22         no
## 526  40.13406     quad       50       0       mean relative 0 0.007283222          22         no
## 1067 40.16965     quad       50       0       mean relative 0 0.007214973          22         no
## 579  40.18509     quad       50       0       mean relative 0 0.007078698          22         no
```

```
## 1209 40.16699     quad       50       0       mean relative 0 0.007046612          22          no
## 973  40.18940     quad       50       0       mean relative 0 0.006860802          22          no
## 931  40.19451     quad       50       0       mean relative 0 0.006642171          22          no
## 1063 40.17907     quad       50       0       mean relative 0 0.006614238          22          no
## 1017 40.18465     quad       50       0       mean relative 0 0.006446643          22          no
## 510  40.21307     quad       50       0       mean relative 0 0.006273789          22          no
## 1021 40.17428     quad       50       0       mean relative 0 0.006258073          22          no
## 480  40.19762     quad       50       0       mean relative 0 0.006254908          22          no
## 944  40.16213     quad       50       0       mean relative 0 0.006029234          22          no
## 976  40.18070     quad       50       0       mean relative 0 0.006025337          22          no
## 1210 40.16909     quad       50       0       mean relative 0 0.005958144          22          no
```

The best windows (deltaAICc difference from the best model < 2) for the linear+quadratic models sorted by deltaAICc. All models with the lowest AICc present very comparable windows:

```
sternum_50_bestmod <- subset(
  sternum_50_sw[[n_bestmod_sternum_50]]$Dataset,
  deltaAICc < sternum_50_sw[[n_bestmod_sternum_50]]$Dataset$deltaAICc[1] + 2
)
sternum_50_bestmod
```

```
##      deltaAICc WindowOpen WindowClose ModelBeta  Std.Error ModelBetaQ Std.ErrorQ ModelBetaC
## 512  -6.921814         31          16 0.6439868 0.04890045 -0.4618389 0.06793423         NA
## 513  -6.567573         31          15 0.6923178 0.04888465 -0.4919496 0.06791674         NA
## 545  -6.054905         32          16 0.6681248 0.04894310 -0.4998184 0.06789905         NA
## 514  -5.998077         31          14 0.7277946 0.04889078 -0.5134053 0.06788579         NA
## 546  -5.799299         32          15 0.7153519 0.04891801 -0.5325067 0.06787628         NA
## 974  -5.526967         43          16 0.8627472 0.04896451 -0.7063225 0.06795367         NA
## 1019 -5.476339         44          16 0.8637459 0.04896773 -0.6758509 0.06798146         NA
## 1065 -5.469180         45          16 0.8657824 0.04898187 -0.6529619 0.06801712         NA
## 1066 -5.062738         45          15 0.8729279 0.04899598 -0.6545456 0.06799652         NA
## 930  -5.000570         42          16 0.8194125 0.04897776 -0.6897538 0.06795795         NA
## 511  -5.000428         31          17 0.5647773 0.04895028 -0.4018979 0.06797507         NA
## 1018 -4.955888         44          17 0.8356887 0.04897945 -0.6573560 0.06805801         NA
## 975  -4.940319         43          15 0.8651537 0.04897529 -0.7012805 0.06793445         NA
## 1064 -4.939830         45          17 0.8360772 0.04899397 -0.6333999 0.06809447         NA
##      ModelInt Function Furthest Closest Statistics     Type K   ModWeight sample.size Randomised
## 512  40.19533     quad       50       0       mean relative 0 0.020872955          22          no
## 513  40.18964     quad       50       0       mean relative 0 0.017484832          22          no
## 545  40.19438     quad       50       0       mean relative 0 0.013531225          22          no
## 514  40.18390     quad       50       0       mean relative 0 0.013152155          22          no
## 546  40.18927     quad       50       0       mean relative 0 0.011907840          22          no
## 974  40.18512     quad       50       0       mean relative 0 0.010391942          22          no
## 1019 40.17877     quad       50       0       mean relative 0 0.010132185          22          no
## 1065 40.17287     quad       50       0       mean relative 0 0.010095982          22          no
## 1066 40.17256     quad       50       0       mean relative 0 0.008239309          22          no
## 930  40.19599     quad       50       0       mean relative 0 0.007987137          22          no
## 511  40.20409     quad       50       0       mean relative 0 0.007986572          22          no
## 1018 40.18291     quad       50       0       mean relative 0 0.007810675          22          no
## 975  40.18407     quad       50       0       mean relative 0 0.007750109          22          no
## 1064 40.17707     quad       50       0       mean relative 0 0.007748212          22          no
```

Windows plot It's possible to extract the time windows of all the best supported models (i.e. multi-model inference). This panel shows the opening and closing points of the time windows that were best supported

by the data, here those models that made up 95% model confidence set.

```
plotwin(sternum_50_sw[[n_bestmod_sternum_50]]$Dataset)
```

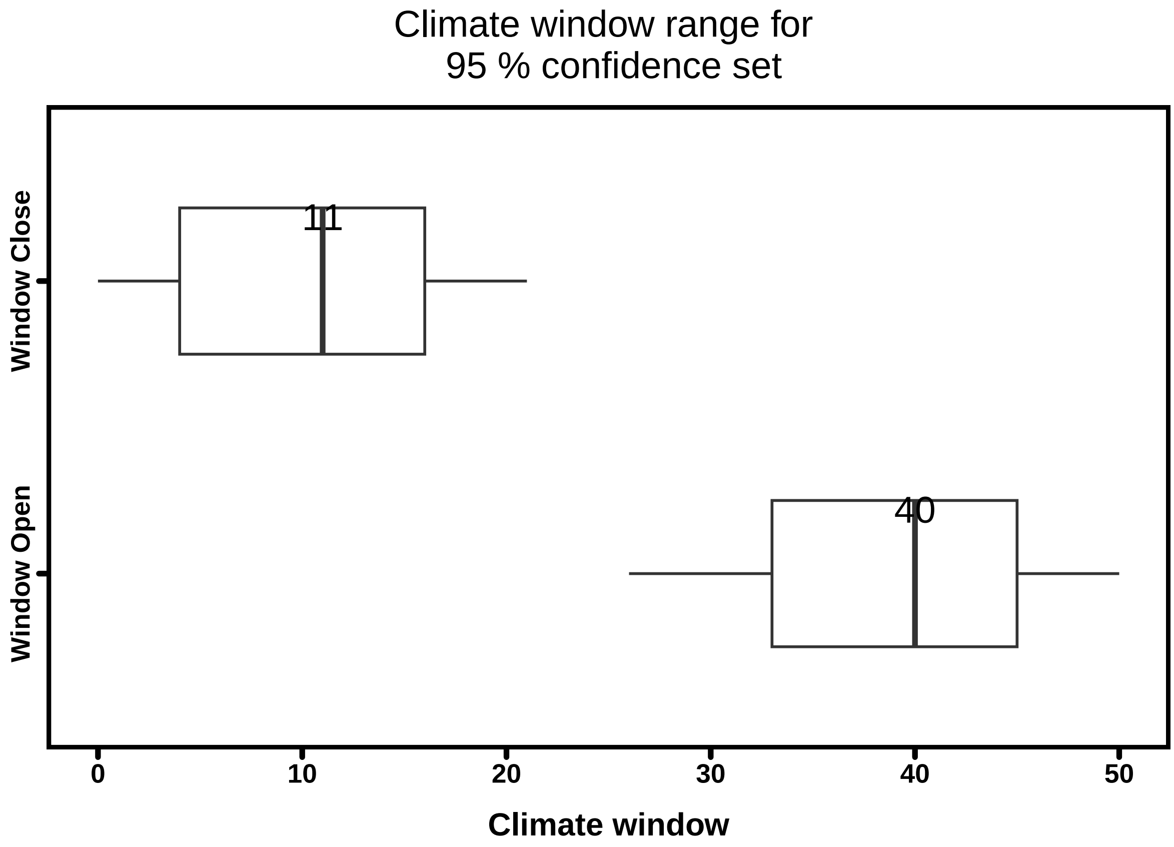

Delta plot The variation in deltaAICc between time windows can be better investigated using the following plot:

```
plotdelta(
  dataset =
    sternum_50_sw[[n_bestmod_sternum_50]]$Dataset, arrow = TRUE
)
```

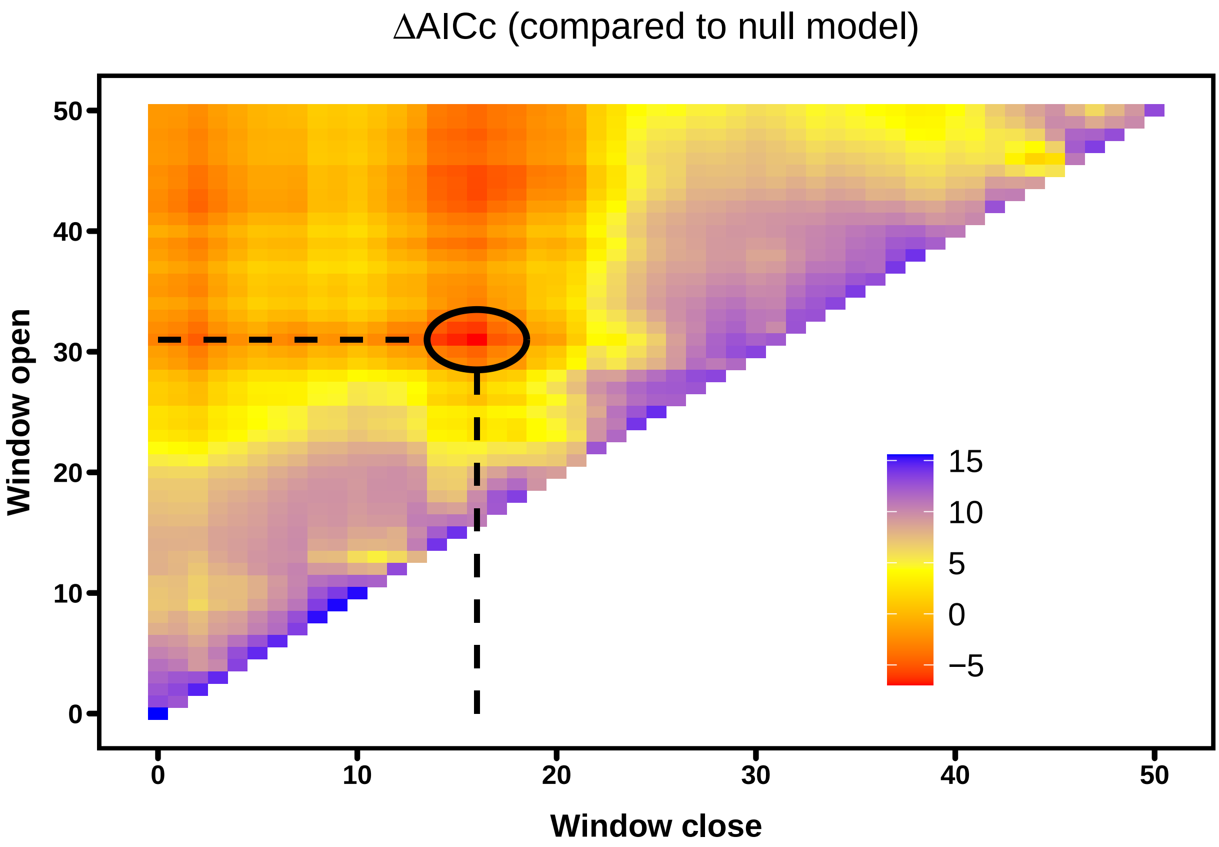

Warmer areas shows values with the lowest deltaAICc (i.e. "best models"). As explained by van de Pol et al., 2016, these deltaAICc landscapes of the different time windows shows multiple peaks (red areas) instead of a clear single peak. This can indicate the presence of multiple (e.g. possibly both long- and short-lag) weather signals within the same weather variable, but it can also occur due to collinearity or chance.

The evidence for multiple signals can be therefore investigated by adding the best supported of the weather windows to the baseline model, and re-fitting all the different time windows again: this tests whether there is still strong model support for the second best (e.g. short-lag) weather window once the other best supported (e.g. long-lag) weather window has been accounted for in the baseline model (here in the Step 2).

Beta plot This panel shows the model support (deltaAICc) for all fitted time windows tried, shown for each combination of Window open (y-axis) and Window close (x-axis). Models with the lowest deltaAICc (red) are the best supported (colours show the deltaAICc levels compared to the null model, see legend). Strongly supported windows will often be grouped together.

```
plotbetas(sternum_50_sw[[n_bestmod_sternum_50]]$Dataset, arrow = TRUE)
```

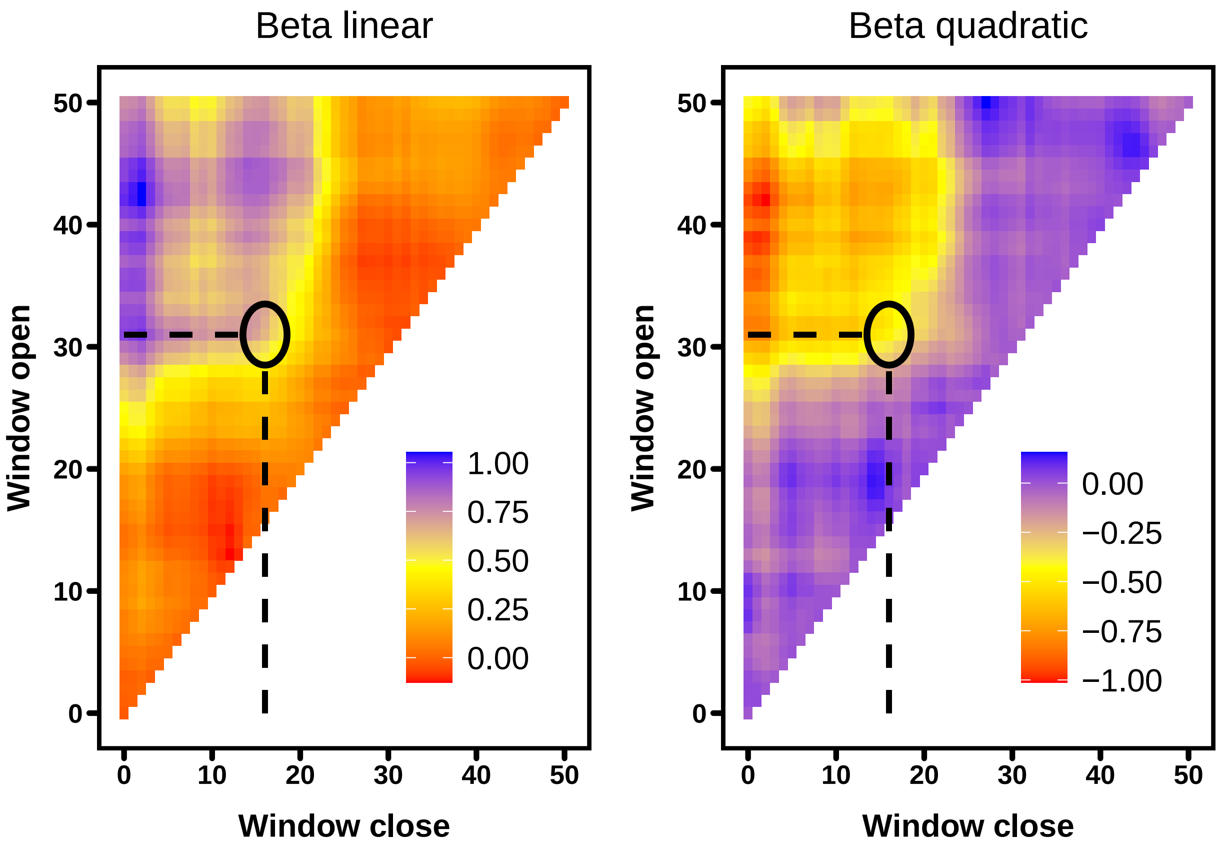

Autocollinearity Correlation between the mean temperature during the best supported time window and the mean temperature over all other time windows.

```
autocoll <- autowin(
  reference = sternum_50_sw[[n_bestmod_sternum_50]],
  baseline = sternum_50_basemod,
  xvar = list(
    tr_pc1 = data_clim_sub$tr_comp_1
  ),
  type = "relative",
  range = c(50, 0),
  stat = "mean",
  cdate = data_clim_sub$date,
  bdate = data_am_nestlings_sternum_50$date_50days,
  func = "quad",
  # cmissing = FALSE,
  cinterval = "day"
)

save(autocoll, file = "output/sternum_50_autocall.rda")
```

```
load(file = "output/sternum_50_autocall.rda")
plotcor(autocoll, type = "A", arrow = TRUE)
```

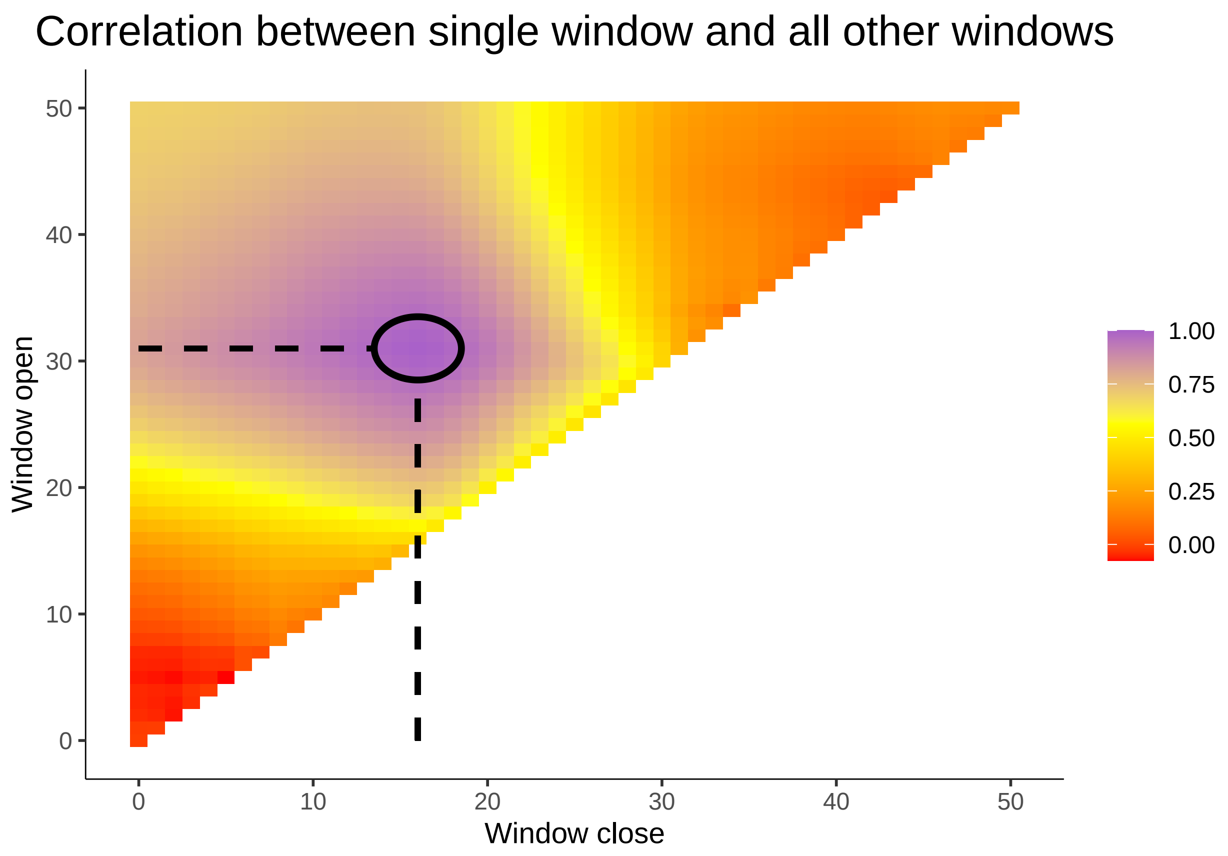

Randwin Using randwin to randomize the identity of the nestling, we are able to check if the window that was found before is actually important, or the relationship was just random.

```
# Performing randamization to identify
# likelyhood of of dignals occuring by chance

sternum_50_rand1000 <- randwin(
  repeats = 1000,
  baseline = sternum_50_basemod,
  xvar = list(tr_pc1 = data_clim_sub$tr_comp_1),
  stat = c("mean"),
  func = c("quad"),
  type = "relative", # relative to the individual
  range = c(50, 0),
  cinterval = "day",
  cdate = data_clim_sub$date,
  bdate = data_am_nestlings_sternum_50$date_50days,
  cmissing = FALSE
)
save(sternum_50_rand1000, file = "output/sternum_50_rand1000.rda")
```

```
load("output/sternum_50_rand1000.rda")

climwin::pvalue(
  datasetrand = sternum_50_rand1000[[1]],
  dataset = sternum_50_sw[[n_bestmod_sternum_50]]$Dataset,
```

```
  metric = "AIC",
  sample.size = sternum_50_sw[[n_bestmod_sternum_50]]$Dataset$sample.size[1]
)
```

```
## [1] 0.012
```

This panel shows a histogram of all deltaAICc values from all models run on the randomized (in grey) and the deltaAICc of the best model fitted on the observed data (dashed line). The best climate window in the randomized data is by definition a false positive, as the randomization procedure removes any climate signal from the data. The percentage of randomizations that generates a deltaAICc value that is at least as low as the deltaAICc value of the best model fitted to the observed data (P value above) is a measure of how likely it is that one obtained the observed deltaAICc by chance and thus whether the signal is likely to be real or not. In our case we can say that the candidate signal is very unlikely to be a false positive: $P < 0.05$.

```
climwin::plothist(
  datasetrand = sternum_50_rand1000[[1]],
  dataset = sternum_50_sw[[n_bestmod_sternum_50]]$Dataset
)
```

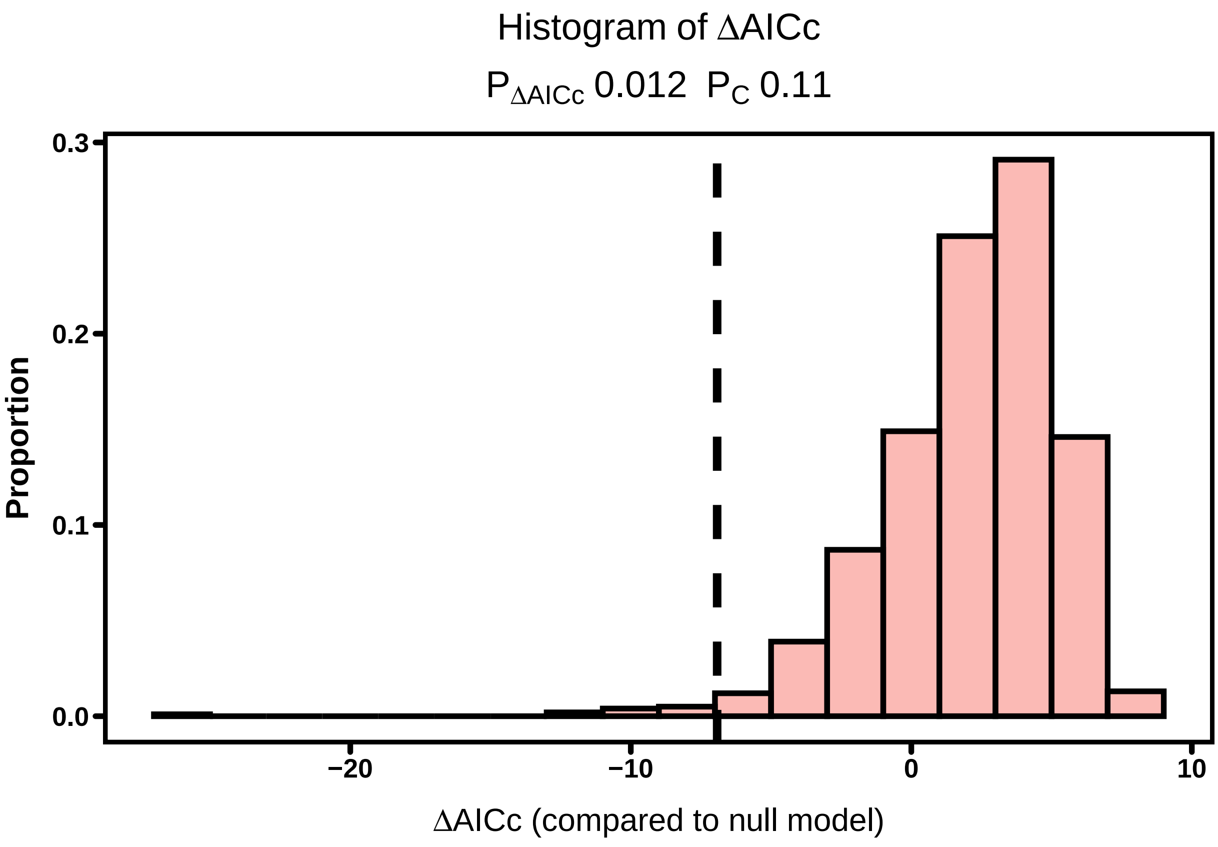

Extracting the best window The best window was obtaining averaging the opening and closing dates for all the best models (deltaAICc < 2). The best window opened 38.5 (mean; SD: 6.5) and closed 15.79 (mean; SD: 0.89) days before the 50 days measurement. Which corresponds to the age of the nestling of 12 and 34 days old.

```r
sternum_50_bestsw <- singlewin(
  baseline = sternum_50_basemod,
  xvar = list(
    tr_pc1 = data_clim_sub$tr_comp_1
  ),
  stat = c("mean"),
  func = c("quad"),
  type = "relative",
  range = c(
    round(mean(sternum_50_bestmod$WindowOpen), 0),
    round(mean(sternum_50_bestmod$WindowClose), 0)
  ),
  cinterval = "day",
  cdate = data_clim_sub$date,
  bdate = data_am_nestlings_sternum_50$date_50days
)
```

```
## fixed-effect model matrix is rank deficient so dropping 2 columns / coefficients
```

```r
# adding the climatic variable to the sternum_50 dataset
data_am_nestlings_sternum_50$signal_for_sternum_50 <-
  sternum_50_bestsw$BestModelData$climate
```

Body mass at 50 days The linear+quadratic term for the mean temperature better explains the variation in the data (deltaAICc has the lowest value), sorted by deltaAICc such that the best supported model is on top.

```r
mass_50_mod_combos <- (mass_50_sw$combos)

mass_50_mod_combos[order(mass_50_mod_combos$DeltaAICc), ]
```

```
##   response   climate     type stat func DeltaAICc WindowOpen WindowClose
## 6  mass_50 temp_mean relative mean quad    -64.27          2           0
## 5  mass_50    tr_pc1 relative mean quad    -56.32          4           0
## 3  mass_50 rain_mean relative mean  lin    -51.49          9           0
## 7  mass_50 rain_mean relative mean quad    -46.89          9           0
## 8  mass_50 wind_mean relative mean quad    -40.15         45           8
## 1  mass_50    tr_pc1 relative mean  lin    -39.94          4           0
## 2  mass_50 temp_mean relative mean  lin    -35.18          1           0
## 4  mass_50 wind_mean relative mean  lin    -18.87         45          40
```

```r
n_bestmod_mass_50 <- as.numeric(rownames(
  mass_50_mod_combos[order(mass_50_mod_combos$DeltaAICc), ]
))[1]
```

The best quadratic models The 30 best windows for the linear+quadratic models sorted by deltaAICc. To investigate any other tested hypothesis we can simply replace the number on the left or in the double square brackets with the corresponding list number.

```r
head(mass_50_sw[[n_bestmod_mass_50]]$Dataset, 50)
```

```
##       deltaAICc WindowOpen WindowClose  ModelBeta Std.Error   ModelBetaQ Std.ErrorQ ModelBetaC
## 6    -64.265745          2           0  8.0014807 0.3184047 -0.192959345  0.4563156         NA
## 3    -57.417441          1           0  5.9146946 0.3188554 -0.139728688  0.4574132         NA
## 2    -48.801874          1           1  5.2562216 0.3202113 -0.124370013  0.4592101         NA
## 5    -45.503437          2           1  6.5996671 0.3208174 -0.159444724  0.4595184         NA
## 1    -44.871127          0           0  4.5530382 0.3198698 -0.105871770  0.4591271         NA
## 10   -44.739372          3           0  8.0230803 0.3208091 -0.193644153  0.4599293         NA
## 121  -35.271271         15          15 -5.7236610 0.3219224  0.145600952  0.4689043         NA
## 122  -28.813650         15          14 -5.9096382 0.3225436  0.150690114  0.4691278         NA
## 15   -24.884130          4           0  6.7699591 0.3233282 -0.161733539  0.4642305         NA
## 139  -24.053360         16          14 -5.9596517 0.3232328  0.150508352  0.4713939         NA
## 9    -23.215307          3           1  5.9924116 0.3234409 -0.144702742  0.4639332         NA
## 138  -18.398435         16          15 -4.6501193 0.3239311  0.116241153  0.4718085         NA
## 4    -16.345357          2           2  4.2267672 0.3241902 -0.101840063  0.4655616         NA
## 157  -15.395147         17          14 -5.4872152 0.3243518  0.138185591  0.4732694         NA
## 140  -15.375912         16          13 -5.6176560 0.3240217  0.140428808  0.4723567         NA
## 1279 -12.905738         50          47  1.3678827 0.3240612 -0.049829258  0.4681603         NA
## 123  -12.371086         15          13 -4.9925901 0.3242315  0.125637616  0.4710788         NA
## 1278 -12.213769         50          48  1.4118977 0.3240720 -0.050341022  0.4678141         NA
## 106  -12.203046         14          14 -4.0960462 0.3242509  0.104723174  0.4697100         NA
## 1226 -12.191126         49          49  1.4532133 0.3240324 -0.050132671  0.4676623         NA
## 1280 -12.124291         50          46  1.2680288 0.3243788 -0.047418607  0.4687205         NA
## 21   -12.027305          5           0  5.7528748 0.3249174 -0.137258129  0.4679125         NA
## 1277 -11.966925         50          49  0.9682711 0.3241553 -0.037986143  0.4677597         NA
## 158  -11.823227         17          13 -5.5029344 0.3246075  0.137192885  0.4738899         NA
## 1281 -11.015150         50          45  0.8550540 0.3247216 -0.036783970  0.4691790         NA
## 176   -9.953076         18          14 -5.3554141 0.3250558  0.135516343  0.4741460         NA
## 177   -9.322745         18          13 -5.6945928 0.3250141  0.142770026  0.4746506         NA
## 1227  -9.007762         49          48  1.5543038 0.3244950 -0.052383371  0.4684170         NA
## 1276  -8.385601         50          50  0.4477120 0.3247501 -0.022686123  0.4684220         NA
## 156   -8.291613         17          15 -3.9568110 0.3251558  0.098331201  0.4731107         NA
## 1228  -8.140613         49          47  1.2620144 0.3246275 -0.044991248  0.4690207         NA
## 1282  -7.075916         50          44  0.2410714 0.3254830 -0.019691003  0.4700414         NA
## 1229  -7.037589         49          46  1.0705981 0.3249336 -0.040124275  0.4696200         NA
## 14    -6.862556          4           1  4.3699728 0.3255317 -0.103658302  0.4682194         NA
## 1230  -6.046203         49          45  0.5746021 0.3252348 -0.027306799  0.4700261         NA
## 1284  -5.702757         50          42 -0.9928712 0.3262797  0.013024044  0.4704723         NA
## 1283  -5.603272         50          43 -0.4015480 0.3259940 -0.002348656  0.4703643         NA
## 28    -5.570170          6           0  5.1919576 0.3257501 -0.124269637  0.4703250         NA
## 1285  -5.550433         50          41 -1.0187081 0.3264131  0.013189168  0.4706785         NA
## 1288  -5.370280         50          38 -4.3638939 0.3267350  0.102250124  0.4707039         NA
## 141   -5.349017         16          12 -4.5146059 0.3252159  0.111040645  0.4732118         NA
## 178   -5.335599         18          12 -5.2555336 0.3254513  0.130259952  0.4748469         NA
## 159   -5.333387         17          12 -4.6999299 0.3253386  0.115365050  0.4742245         NA
## 1287  -4.739701         50          39 -3.3248530 0.3266830  0.074979745  0.4707444         NA
## 197   -4.672331         19          13 -5.5028451 0.3256010  0.138793708  0.4753384         NA
## 1291  -4.380901         50          35 -6.3747139 0.3268649  0.155041525  0.4717930         NA
## 1286  -4.371365         50          40 -2.0213878 0.3266735  0.040255241  0.4707860         NA
## 1138  -4.186436         47          38 -5.9523265 0.3268705  0.149630331  0.4704765         NA
## 1290  -4.043183         50          36 -5.2365932 0.3269496  0.125004082  0.4714795         NA
## 965   -3.974394         43          25 13.3421156 0.3259410 -0.339959105  0.4743405         NA
```

```
##       ModelInt Function Furthest Closest Statistics     Type K    ModWeight sample.size
## 6     18.90363     quad       50       0       mean relative 0 9.678487e-01          25
## 3     39.06261     quad       50       0       mean relative 0 3.152951e-02          25
## 2     45.93467     quad       50       0       mean relative 0 4.244931e-04          25
## 5     33.21668     quad       50       0       mean relative 0 8.158757e-05          25
## 1     52.53170     quad       50       0       mean relative 0 5.947297e-05          25
## 10    18.60605     quad       50       0       mean relative 0 5.568130e-05          25
## 121  154.78937     quad       50       0       mean relative 0 4.894829e-07          25
## 122  156.45590     quad       50       0       mean relative 0 1.938576e-08          25
## 15    30.62282     quad       50       0       mean relative 0 2.717680e-09          25
## 139  157.66145     quad       50       0       mean relative 0 1.793903e-09          25
## 9     39.20231     quad       50       0       mean relative 0 1.179827e-09          25
## 138  145.37933     quad       50       0       mean relative 0 1.061323e-10          25
## 4     57.08835     quad       50       0       mean relative 0 3.802134e-11          25
## 157  153.37041     quad       50       0       mean relative 0 2.364243e-11          25
## 140  154.98822     quad       50       0       mean relative 0 2.341613e-11          25
## 1279  91.67474     quad       50       0       mean relative 0 6.809631e-12          25
## 123  148.43010     quad       50       0       mean relative 0 5.212252e-12          25
## 1278  91.05801     quad       50       0       mean relative 0 4.817976e-12          25
## 106  138.79694     quad       50       0       mean relative 0 4.792212e-12          25
## 1226  90.34165     quad       50       0       mean relative 0 4.763736e-12          25
## 1280  92.66743     quad       50       0       mean relative 0 4.607173e-12          25
## 21    40.87098     quad       50       0       mean relative 0 4.389089e-12          25
## 1277  94.90347     quad       50       0       mean relative 0 4.258562e-12          25
## 158  154.11470     quad       50       0       mean relative 0 3.963323e-12          25
## 1281  96.53364     quad       50       0       mean relative 0 2.645987e-12          25
## 176  151.94715     quad       50       0       mean relative 0 1.555827e-12          25
## 177  155.80137     quad       50       0       mean relative 0 1.135237e-12          25
## 1227  89.25976     quad       50       0       mean relative 0 9.698152e-13          25
## 1276  99.11526     quad       50       0       mean relative 0 7.105399e-13          25
## 156  138.91248     quad       50       0       mean relative 0 6.779210e-13          25
## 1228  92.04857     quad       50       0       mean relative 0 6.286225e-13          25
## 1282 101.88165     quad       50       0       mean relative 0 3.691425e-13          25
## 1229  93.86256     quad       50       0       mean relative 0 3.621357e-13          25
## 14    54.90272     quad       50       0       mean relative 0 3.317902e-13          25
## 1230  98.48596     quad       50       0       mean relative 0 2.205944e-13          25
## 1284 113.25103     quad       50       0       mean relative 0 1.857874e-13          25
## 1283 107.68886     quad       50       0       mean relative 0 1.767719e-13          25
## 28    46.68107     quad       50       0       mean relative 0 1.738703e-13          25
## 1285 113.69355     quad       50       0       mean relative 0 1.721628e-13          25
## 1288 144.70749     quad       50       0       mean relative 0 1.573329e-13          25
## 141  144.93189     quad       50       0       mean relative 0 1.556691e-13          25
## 178  152.11223     quad       50       0       mean relative 0 1.546283e-13          25
## 159  146.94491     quad       50       0       mean relative 0 1.544573e-13          25
## 1287 134.91394     quad       50       0       mean relative 0 1.147866e-13          25
## 197  153.69299     quad       50       0       mean relative 0 1.109844e-13          25
## 1291 163.79464     quad       50       0       mean relative 0 9.593540e-14          25
## 1286 122.83998     quad       50       0       mean relative 0 9.547908e-14          25
## 1138 157.76346     quad       50       0       mean relative 0 8.704648e-14          25
## 1290 153.07979     quad       50       0       mean relative 0 8.102974e-14          25
## 965  -29.87635     quad       50       0       mean relative 0 7.829012e-14          25
##      Randomised
## 6            no
## 3            no
```

```
## 2              no
## 5              no
## 1              no
## 10             no
## 121            no
## 122            no
## 15             no
## 139            no
## 9              no
## 138            no
## 4              no
## 157            no
## 140            no
## 1279           no
## 123            no
## 1278           no
## 106            no
## 1226           no
## 1280           no
## 21             no
## 1277           no
## 158            no
## 1281           no
## 176            no
## 177            no
## 1227           no
## 1276           no
## 156            no
## 1228           no
## 1282           no
## 1229           no
## 14             no
## 1230           no
## 1284           no
## 1283           no
## 28             no
## 1285           no
## 1288           no
## 141            no
## 178            no
## 159            no
## 1287           no
## 197            no
## 1291           no
## 1286           no
## 1138           no
## 1290           no
## 965            no
```

The best windows (deltaAICc difference from the best model < 2) for the linear+quadratic models sorted by deltaAICc. There is only one best window:

```r
mass_50_bestmod <- subset(
  mass_50_sw[[n_bestmod_mass_50]]$Dataset,
  deltaAICc < mass_50_sw[[n_bestmod_mass_50]]$Dataset$deltaAICc[1] + 2
)

mass_50_bestmod
```

```
##   deltaAICc WindowOpen WindowClose ModelBeta Std.Error ModelBetaQ Std.ErrorQ ModelBetaC ModelInt
## 6 -64.26574          2           0  8.001481 0.3184047 -0.1929593  0.4563156         NA 18.90363
##   Function Furthest Closest Statistics     Type K ModWeight sample.size Randomised
## 6     quad       50       0       mean relative 0 0.9678487          25         no
```

As it can be seen here, all the other models have a deltaAICc difference from the best model much larger than 2.

```r
head(mass_50_sw[[n_bestmod_mass_50]]$Dataset)
```

```
##    deltaAICc WindowOpen WindowClose ModelBeta Std.Error ModelBetaQ Std.ErrorQ ModelBetaC
## 6  -64.26574          2           0  8.001481 0.3184047 -0.1929593  0.4563156         NA
## 3  -57.41744          1           0  5.914695 0.3188554 -0.1397287  0.4574132         NA
## 2  -48.80187          1           1  5.256222 0.3202113 -0.1243700  0.4592101         NA
## 5  -45.50344          2           1  6.599667 0.3208174 -0.1594447  0.4595184         NA
## 1  -44.87113          0           0  4.553038 0.3198698 -0.1058718  0.4591271         NA
## 10 -44.73937          3           0  8.023080 0.3208091 -0.1936442  0.4599293         NA
##    ModelInt Function Furthest Closest Statistics     Type K    ModWeight sample.size Randomised
## 6  18.90363     quad       50       0       mean relative 0 9.678487e-01          25         no
## 3  39.06261     quad       50       0       mean relative 0 3.152951e-02          25         no
## 2  45.93467     quad       50       0       mean relative 0 4.244931e-04          25         no
## 5  33.21668     quad       50       0       mean relative 0 8.158757e-05          25         no
## 1  52.53170     quad       50       0       mean relative 0 5.947297e-05          25         no
## 10 18.60605     quad       50       0       mean relative 0 5.568130e-05          25         no
```

Windows plot It's possible to extract the time windows of all the best supported models (i.e. multi-model inference). This panel shows the opening and closing points of the time windows that were best supported by the data, here those models that made up 95% model confidence set.

```r
plotwin(mass_50_sw[[n_bestmod_mass_50]]$Dataset)
```

```
## Warning in plotwin(mass_50_sw[[n_bestmod_mass_50]]$Dataset): Top window has a weight greater
## than 0.95. Plotting single best window only.
```

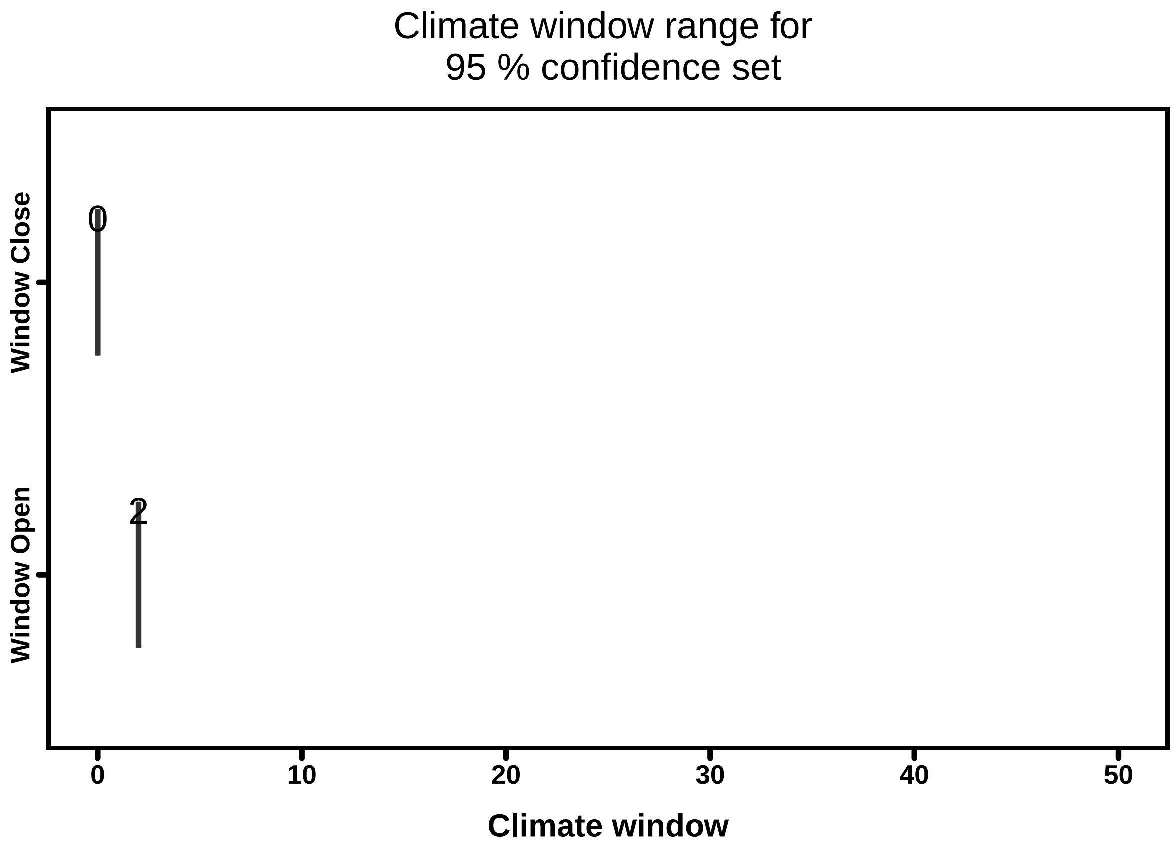

Delta plot The variation in deltaAICc between time windows can be better investigated using the following plot:

```
plotdelta(
  dataset =
    mass_50_sw[[n_bestmod_mass_50]]$Dataset, arrow = TRUE
)
```

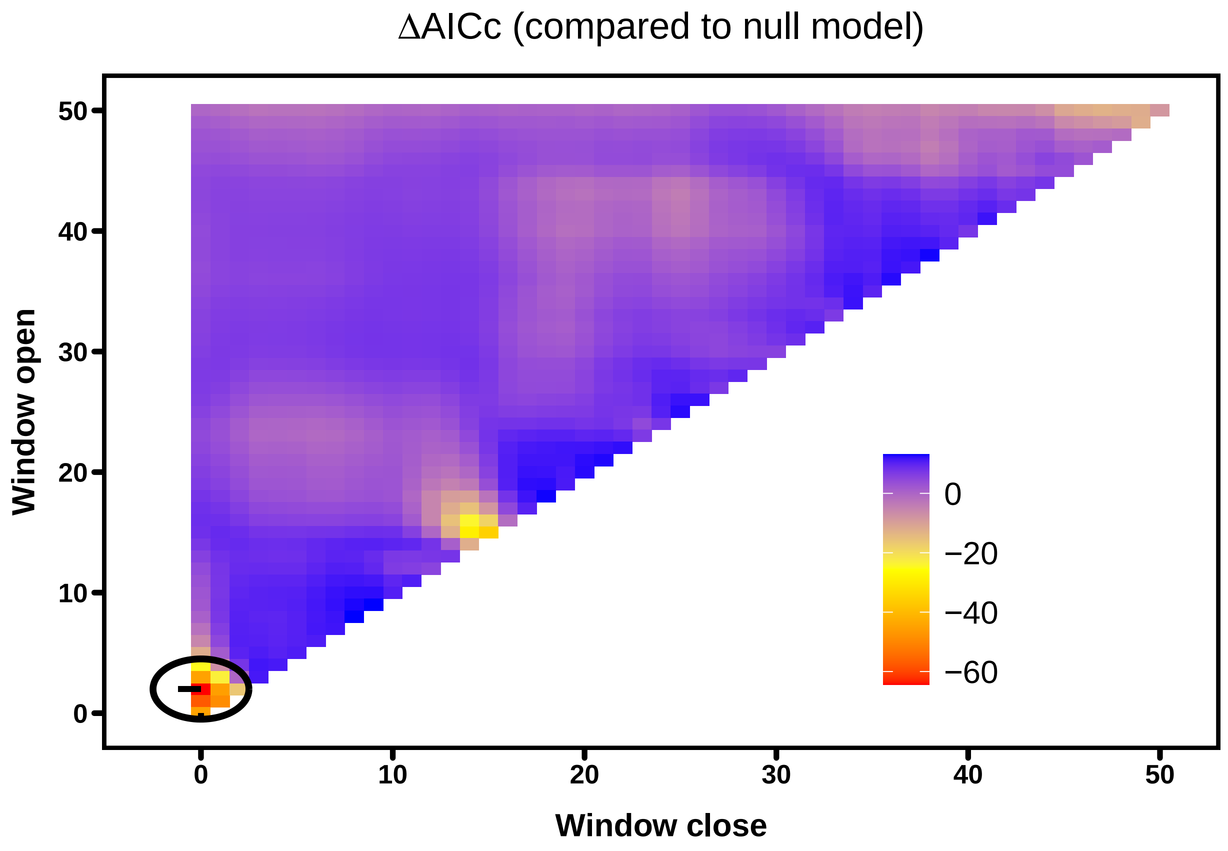

Warmer areas shows values with the lowest deltaAICc (i.e. "best models"). As explained by van de Pol et al., 2016, these deltaAICc landscapes of the different time windows shows multiple peaks (red areas) instead of a clear single peak. This can indicate the presence of multiple (e.g. possibly both long- and short-lag) weather signals within the same weather variable, but it can also occur due to collinearity or chance.

The evidence for multiple signals can be therefore investigated by adding the best supported of the weather windows to the baseline model, and re-fitting all the different time windows again: this tests whether there is still strong model support for the second best (e.g. short-lag) weather window once the other best supported (e.g. long-lag) weather window has been accounted for in the baseline model (here in the Step 2).

Beta plot This panel shows the model support (deltaAICc) for all fitted time windows tried, shown for each combination of Window open (y-axis) and Window close (x-axis). Models with the lowest deltaAICc (red) are the best supported (colours show the deltaAICc levels compared to the null model, see legend). Strongly supported windows will often be grouped together.

```
plotbetas(mass_50_sw[[n_bestmod_mass_50]]$Dataset, arrow = TRUE)
```

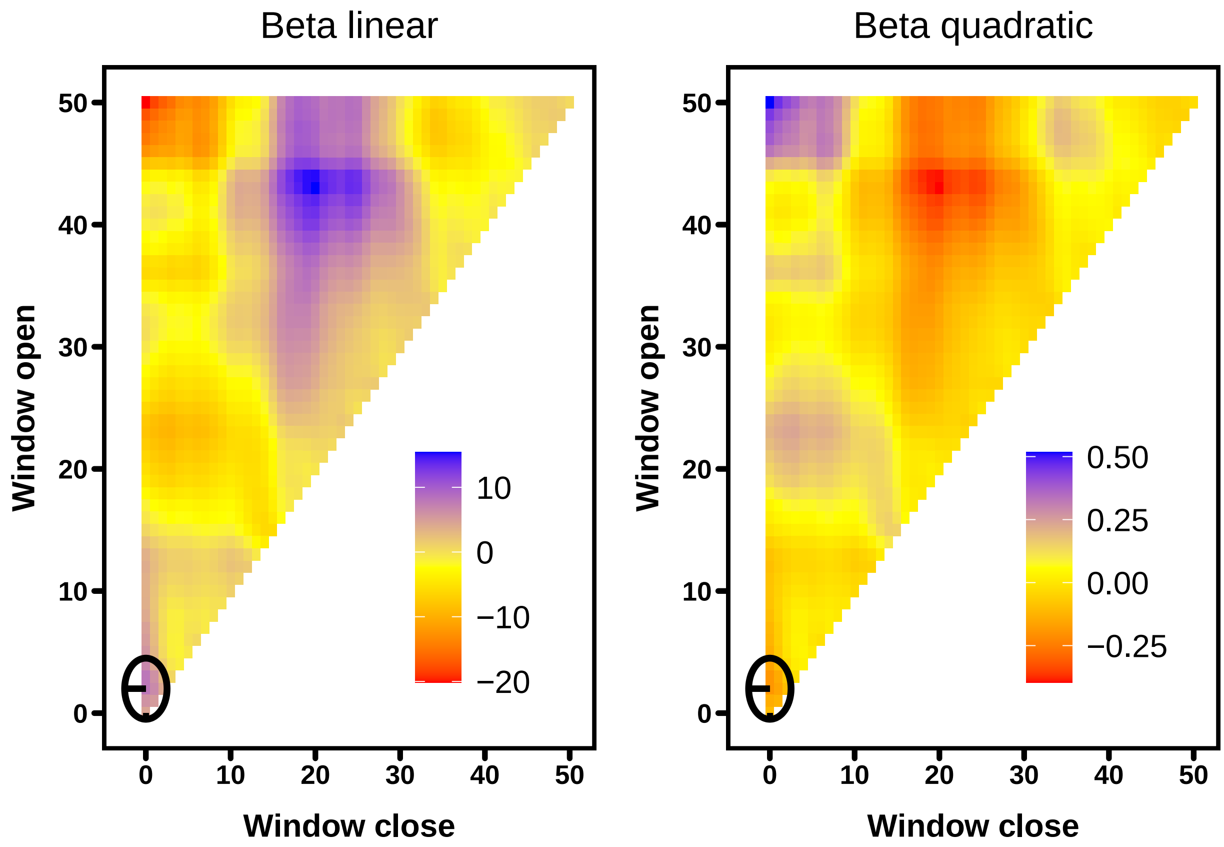

Autocollinearity Correlation between the mean temperature during the best supported time window and the mean temperature over all other time windows.

```
autocoll <- autowin(
  reference = mass_50_sw[[n_bestmod_mass_50]],
  baseline = mass_50_basemod,
  xvar = list(
    tr_pc1 = data_clim_sub$tr_comp_1
  ),
  type = "relative",
  range = c(50, 0),
  stat = "mean",
  cdate = data_clim_sub$date,
  bdate = data_am_nestlings_mass_50$date_50days,
  func = "quad",
  # cmissing = FALSE,
  cinterval = "day"
)

save(autocoll, file = "output/mass_50_autocall.rda")
```

```
load(file = "output/mass_50_autocall.rda")
plotcor(autocoll, type = "A", arrow = TRUE)
```

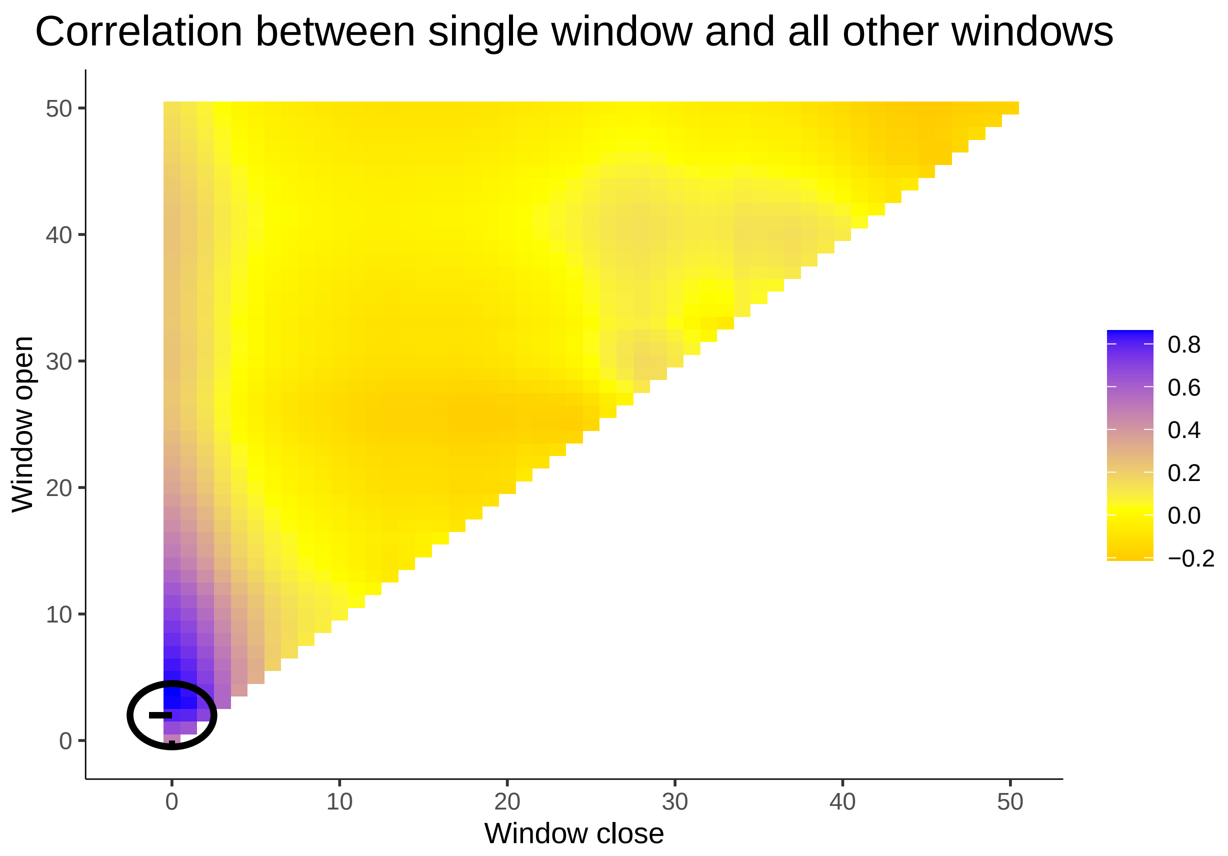

Randwin Using randwin to randomize the identity of the nestling, we are able to check if the window that was found before is actually important, or the relationship was just random.

```
# Performing randamization to identify
# likelyhood of of dignals occuring by chance

mass_50_rand1000 <- randwin(
  repeats = 1000,
  baseline = mass_50_basemod,
  xvar = list(temp_mean = data_clim_sub$T.daily.mean),
  stat = c("mean"),
  func = c("quad"),
  type = "relative", # relative to the individual
  range = c(50, 0),
  cinterval = "day",
  cdate = data_clim_sub$date,
  bdate = data_am_nestlings_mass_50$date_50days,
  cmissing = FALSE
)
save(mass_50_rand1000, file = "output/mass_50_rand1000.rda")
```

```
load("output/mass_50_rand1000.rda")

climwin::pvalue(
  datasetrand = mass_50_rand1000[[1]],
  dataset = mass_50_sw[[n_bestmod_mass_50]]$Dataset,
```

```
  metric = "AIC",
  sample.size = mass_50_sw[[n_bestmod_mass_50]]$Dataset$sample.size[1]
)
```

```
## [1] "<0.001"
```

This panel shows a histogram of all deltaAICc values from all models run on the randomized (in grey) and the deltaAICc of the best model fitted on the observed data (dashed line). The best climate window in the randomized data is by definition a false positive, as the randomization procedure removes any climate signal from the data. The percentage of randomizations that generates a deltaAICc value that is at least as low as the deltaAICc value of the best model fitted to the observed data (P value above) is a measure of how likely it is that one obtained the observed deltaAICc by chance and thus whether the signal is likely to be real or not. In our case we can say that the candidate signal is very unlikely to be a false positive: P <0.001.

```
climwin::plothist(
  datasetrand = mass_50_rand1000[[1]],
  dataset = mass_50_sw[[n_bestmod_mass_50]]$Dataset
)
```

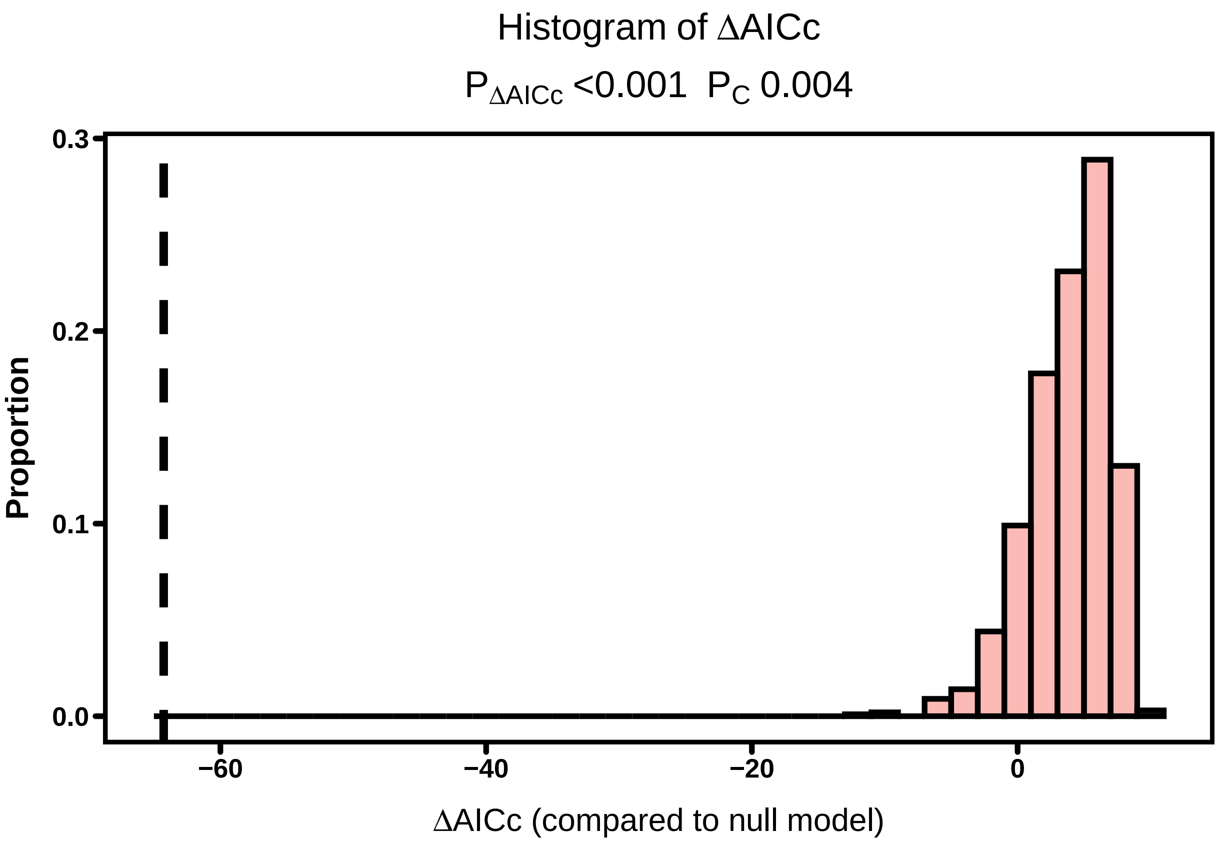

Extracting the best window The best window was obtaining averaging the opening and closing dates for all the best models (deltaAICc < 2). In the case of the body mass there was only one top model and the window opened 2 and closed 0 days before the 50 days measurement, which corresponds to the age of the nestling of 46 and 50 days old.

```
mass_50_bestsw <- singlewin(
  baseline = mass_50_basemod,
  xvar = list(
    # tr_pc1 = data_clim_sub$tr_comp_1
    temp_mean = data_clim_sub$T.daily.mean
  ),
  stat = c("mean"),
  func = c("quad"),
  type = "relative",
  range = c(
    round(mean(mass_50_bestmod$WindowOpen), 0),
    round(mean(mass_50_bestmod$WindowClose), 0)
  ),
  cinterval = "day",
  cdate = data_clim_sub$date,
  bdate = data_am_nestlings_mass_50$date_50days
)
```

```
## fixed-effect model matrix is rank deficient so dropping 2 columns / coefficients
```

```
# adding the climatic variable to the mass_50 dataset
data_am_nestlings_mass_50$signal_for_mass_50 <-
  mass_50_bestsw$BestModelData$climate
```

Adding the variables to the dataset

Extracting values for the best variable within the best window for each trait for each individual so I can use them in a glmm.

```
am_nestlings_clim <- Reduce(function(x, y) merge(x, y, all = TRUE), list(
  data_am_nestlings_wing_50,
  data_am_nestlings_sternum_50,
  data_am_nestlings_mass_50
))
```

Climatic models, quadratic effects are kept even if not significant, if present in the best model suggested by climwin.

```
wing_50_climmod <- lmer(
  wing_50 ~
    brood_size + colony +
    hatch_doy_sc + age_days_sc50 +
    signal_for_wing_50 +
    I(signal_for_wing_50^2) +
    (1 | year_f) + (1 | nestcode_rearing),
  control = lmerControl(optimizer = "bobyqa", optCtrl = list(maxfun = 1e+06)),
  data = data_am_nestlings_wing_50
)

sternum_50_climmod <- lmer(
  sternum_50 ~
```

```r
    brood_size + colony +
    hatch_doy_sc + age_days_sc50 +
    signal_for_sternum_50 +
    I(signal_for_sternum_50^2) +
    (1 | year_f) + (1 | nestcode_rearing),
  control = lmerControl(optimizer = "bobyqa", optCtrl = list(maxfun = 1e+06)),
  data = data_am_nestlings_sternum_50
)

mass_50_climmod <- lmer(
  mass_50 ~
    brood_size + colony +
    hatch_doy_sc + age_days_sc50 +
    signal_for_mass_50 +
    I(signal_for_mass_50^2) +
    (1 | year_f) + (1 | nestcode_rearing),
  control = lmerControl(optimizer = "bobyqa", optCtrl = list(maxfun = 1e+06)),
  data = data_am_nestlings_mass_50
)
```

```r
labels <- c(
  `(Intercept)` = "Intercept",
  hatch_doy_sc = "Hatching day",
  brood_size = "Hatching brood size",
  age_days_sc50 = "Age (days)",
  colonySolothurn = "Colony [Solothurn]",
  signal_for_wing_50 = "PC1 (age: 1-48)",
  `I(signal_for_wing_50^2)` = "PC1 (age: 1-48) 2",
  signal_for_sternum_50 = "PC1 (age: 12-34)",
  `I(signal_for_sternum_50^2)` = "PC1 (age: 12-34) 2",
  signal_for_mass_50 = "Temp (age: 48-50)",
  `I(signal_for_mass_50^2)` = "Temp (age: 48-50) 2"
)

sjPlot::tab_model(
  wing_50_climmod,
  sternum_50_climmod,
  mass_50_climmod,
  file = "output/Tab_wicm_models_50.doc",
  string.est = "Estimate",
  string.se = "SE",
  show.ci = FALSE,
  show.se = TRUE,
  show.stat = FALSE,
  show.df = FALSE,
  pred.labels = labels
)
```

Figure

```r
cowplot::plot_grid(
  fun_climres(wing_50_climmod, data_am_nestlings_wing_50),
```

```r
  fun_climres(sternum_50_climmod, data_am_nestlings_sternum_50),
  fun_climres(mass_50_climmod, data_am_nestlings_mass_50),
  ncol = 1, align = c("v", "h")
)
```

```r
# Save the plot to the figures directory
ggsave("figures/Fig. 2 - traits-weather.png", width = 4, height = 8, dpi = 300)
```

Testing for segments

```r
sternum_50_climlme <- lme(
  sternum_50 ~
    signal_for_sternum_50 +
    # I(signal_for_sternum_50^2) +
    brood_size + colony +
    hatch_doy_sc + age_days_sc50,
  random = list(~ 1 | year_f, ~ 1 | nestcode_rearing),
  control = lmerControl(optimizer = "bobyqa", optCtrl = list(maxfun = 1e+06)),
  data = data_am_nestlings_sternum_50
)

sternum_50_climmod_segmented <- segmented(sternum_50_climlme,
  seg.Z = ~signal_for_sternum_50, psi = 0.4,
  random = list(
    year_f = pdDiag(~ 1 + signal_for_sternum_50 + U),
    nestcode_rearing = pdDiag(~ 1 + signal_for_sternum_50 + U)
  )
)
save(sternum_50_climmod_segmented,
  file = "output/sternum_50_climmod_segmented_04.rda"
)

sternum_50_climmod_segmented <- segmented(sternum_50_climlme,
  seg.Z = ~signal_for_sternum_50, psi = 0,
  random = list(
    year_f = pdDiag(~ 1 + signal_for_sternum_50 + U),
    nestcode_rearing = pdDiag(~ 1 + signal_for_sternum_50 + U)
  )
)
save(sternum_50_climmod_segmented,
  file = "output/sternum_50_climmod_segmented_0.rda"
)

sternum_50_climmod_segmented <- segmented(sternum_50_climlme,
  seg.Z = ~signal_for_sternum_50, psi = 0.8,
  random = list(
    year_f = pdDiag(~ 1 + signal_for_sternum_50 + U),
    nestcode_rearing = pdDiag(~ 1 + signal_for_sternum_50 + U)
  )
)
```

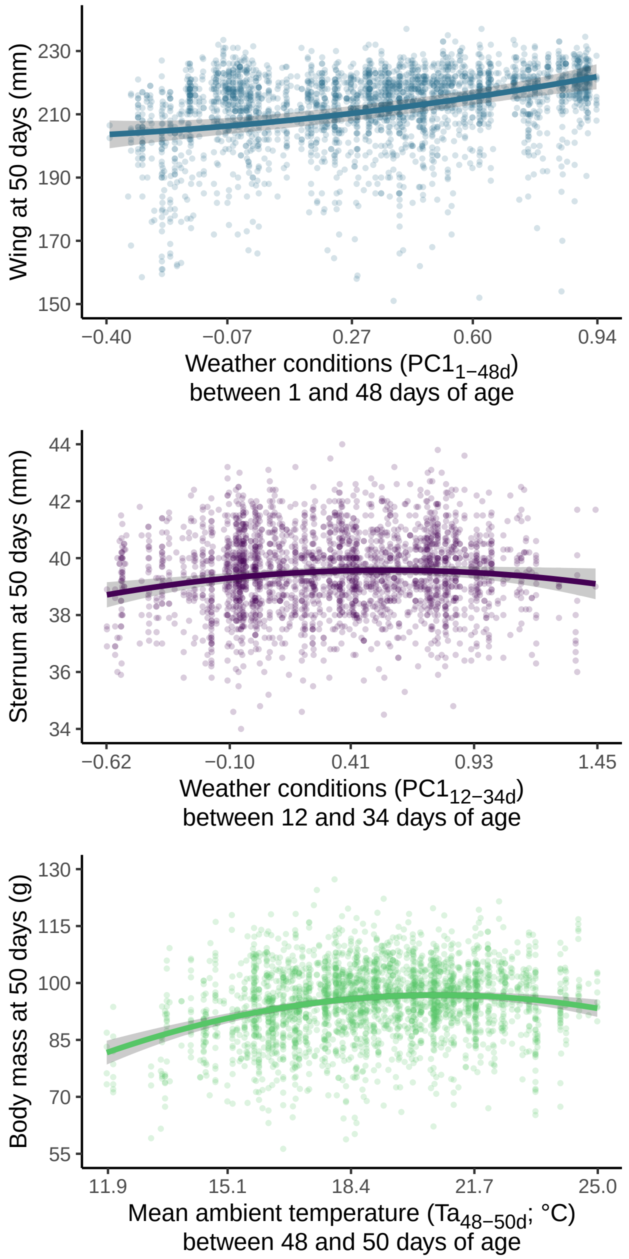

Figure 3: Fig. 2 - Variation in wing length, sternum length and body mass of 50-day-old nestling Alpine swifts in relation to weather conditions encountered earlier in their development. Weather conditions resulted from a principal component analysis (PC1) between daily average temperature and daily rain, and traits were affected by weather conditions over different time windows. Low PC1 values indicate cold and rainy days, and high values indicate sunny and warm days. Solid lines (and 95% confidence intervals) are predictions from the models presented in Table 2. Climatic windows are reported using the nestling's age as a reference.

```r
save(sternum_50_climmod_segmented,
  file = "output/sternum_50_climmod_segmented_08.rda"
)
```

Sternum length Summary of the segmented analysis

psi = 0

```r
load("output/sternum_50_climmod_segmented_0.rda")
summary(sternum_50_climmod_segmented)
```

```
## Segmented mixed-effects model fit by REML
##        AIC      BIC   logLik
##   8498.841 8585.837 -4234.42
##  Bootstrap restarting on 10 samples;  3 different solution(s)
##
## Random effects:
##  Formula: ~1 + signal_for_sternum_50 + U | year_f
##  Structure: Diagonal
##         (Intercept) signal_for_sternum_50            U
## StdDev:   0.2020344             0.3586896 0.0001815599
##
##  Formula: ~1 + signal_for_sternum_50 + U | nestcode_rearing %in% year_f
##  Structure: Diagonal
##         (Intercept) signal_for_sternum_50          U Residual
## StdDev:   0.5877018             0.1656474 0.03823937  1.23168
##
## Fixed effects:
##                             Value Std.Error   DF t-value p-value
## (Intercept)              41.38751   0.84875 1252  48.763   0.000
## bsh_ax                   -0.34937   0.04905 1166  -7.122   0.000
## colonySolothurn           0.17642   0.06835 1166   2.581   0.010
## hatch_doy_sc             -0.22559   0.04076 1252  -5.535   0.000
## age_days_sc50             0.08825   0.02050 1252   4.305   0.000
## -- leftS:
## signal_for_sternum_50  3.42086   1.69671 1252   2.016   0.044
## -- diffS:
## U                        -3.25225   1.70482 1252  -1.908
## -- break:
## G0                       -0.37101   0.08845 1166
##  psi.link = identity
##
## Standardized Within-Group Residuals:
##         Min          Q1         Med          Q3         Max
## -6.12336881 -0.57426371  0.01170877  0.59116152  2.82134160
##
## Number of Observations: 2447
## Number of Groups:
##                         year_f nestcode_rearing %in% year_f
##                             22                         1191
```

```r
mean(sternum_50_climmod_segmented$psi)
```

```
## [1] -0.3552971
```

psi = 0.4

```r
load("output/sternum_50_climmod_segmented_04.rda")
summary(sternum_50_climmod_segmented)
```

```
## Segmented mixed-effects model fit by REML
##        AIC      BIC    logLik
##   8499.856 8586.852 -4234.928
##  Bootstrap restarting on 10 samples;  3 different solution(s)
##
## Random effects:
##  Formula: ~1 + signal_for_sternum_50 + U | year_f
##  Structure: Diagonal
##         (Intercept) signal_for_sternum_50        U
## StdDev:   0.2453159            0.01219222 1.547717
##
##  Formula: ~1 + signal_for_sternum_50 + U | nestcode_rearing %in% year_f
##  Structure: Diagonal
##         (Intercept) signal_for_sternum_50            U Residual
## StdDev:   0.5949472          1.927089e-05 7.278493e-09 1.231978
##
## Fixed effects:
##                           Value Std.Error   DF t-value p-value
## (Intercept)           40.16049   0.15477 1253 259.485  0.0000
## bsh_ax                -0.35298   0.04909 1165  -7.190  0.0000
## colonySolothurn        0.15740   0.06825 1165   2.306  0.0213
## hatch_doy_sc          -0.20187   0.04117 1253  -4.903  0.0000
## age_days_sc50          0.09706   0.02042 1253   4.752  0.0000
## -- leftS:
## signal_for_sternum_50  0.45642   0.15092 1253   3.024  0.0025
## -- diffS:
## U                     -1.68317   1.25816 1165  -1.338
## -- break:
## G0                     0.81258   0.11778 1165
##  psi.link = identity
##
## Standardized Within-Group Residuals:
##          Min           Q1          Med           Q3          Max
## -6.113264015 -0.575664046  0.009521131  0.582781653  2.815703002
##
## Number of Observations: 2447
## Number of Groups:
##                       year_f nestcode_rearing %in% year_f
##                           22                         1191
```

```r
mean(sternum_50_climmod_segmented$psi)
```

```
## [1] 0.8377074
```

psi = 0.8

```
load("output/sternum_50_climmod_segmented_08.rda")
summary(sternum_50_climmod_segmented)
```

```
## Segmented mixed-effects model fit by REML
##        AIC     BIC    logLik
##   8499.863 8586.86 -4234.932
##  Bootstrap restarting on 10 samples;  3 different solution(s)
##
## Random effects:
##  Formula: ~1 + signal_for_sternum_50 + U | year_f
##  Structure: Diagonal
##         (Intercept) signal_for_sternum_50        U
## StdDev:   0.2473956            0.01030922 1.330668
##
##  Formula: ~1 + signal_for_sternum_50 + U | nestcode_rearing %in% year_f
##  Structure: Diagonal
##         (Intercept) signal_for_sternum_50            U Residual
## StdDev:    0.594737          1.882626e-05 9.555731e-09 1.232142
##
## Fixed effects:
##                           Value Std.Error   DF t-value p-value
## (Intercept)           40.15957   0.15500 1253 259.101  0.0000
## bsh_ax                -0.35267   0.04910 1165  -7.182  0.0000
## colonySolothurn        0.15726   0.06834 1165   2.301  0.0216
## hatch_doy_sc          -0.20197   0.04120 1253  -4.902  0.0000
## age_days_sc50          0.09701   0.02043 1253   4.749  0.0000
## -- leftS:
## signal_for_sternum_50  0.46091   0.15169 1253   3.038  0.0024
## -- diffS:
## U                     -1.60754   1.14038 1165  -1.410
## -- break:
## G0                     0.87940   0.11584 1165
##  psi.link = identity
##
## Standardized Within-Group Residuals:
##          Min           Q1          Med           Q3          Max
## -6.113739944 -0.575721368  0.009786115  0.582781417  2.815736437
##
## Number of Observations: 2447
## Number of Groups:
##                       year_f nestcode_rearing %in% year_f
##                           22                         1191
```

```
mean(sternum_50_climmod_segmented$psi)
```

```
## [1] 0.874424
```

The threshold found can be quite different, it seems it's not possible to identify a consistent break point, thus we decided not to use the threshold approach for the sternum length.

```r
mass_50_climlme <- lme(
  mass_50 ~
    signal_for_mass_50 +
    # I(signal_for_mass_50^2) +
    brood_size + colony +
    hatch_doy_sc + age_days_sc50,
  random = list(~ 1 | year_f, ~ 1 | nestcode_rearing),
  control = lmerControl(optimizer = "bobyqa", optCtrl = list(maxfun = 1e+06)),
  data = data_am_nestlings_mass_50
)

mass_50_climmod_segmented <- segmented(mass_50_climlme,
  seg.Z = ~signal_for_mass_50, psi = 20,
  random = list(
    year_f = pdDiag(~ 1 + signal_for_mass_50 + U),
    nestcode_rearing = pdDiag(~ 1 + signal_for_mass_50 + U)
  )
)

save(mass_50_climmod_segmented, file = "output/mass_50_climmod_segmented_20.rda")

mass_50_climmod_segmented <- segmented(mass_50_climlme,
  seg.Z = ~signal_for_mass_50, psi = 19,
  random = list(
    year_f = pdDiag(~ 1 + signal_for_mass_50 + U),
    nestcode_rearing = pdDiag(~ 1 + signal_for_mass_50 + U)
  )
)
save(mass_50_climmod_segmented, file = "output/mass_50_climmod_segmented_19.rda")

mass_50_climmod_segmented <- segmented(mass_50_climlme,
  seg.Z = ~signal_for_mass_50, psi = 21,
  random = list(
    year_f = pdDiag(~ 1 + signal_for_mass_50 + U),
    nestcode_rearing = pdDiag(~ 1 + signal_for_mass_50 + U)
  )
)
save(mass_50_climmod_segmented, file = "output/mass_50_climmod_segmented_21.rda")

mass_50_climmod_segmented <- segmented(mass_50_climlme,
  seg.Z = ~signal_for_mass_50, psi = 22,
  random = list(
    year_f = pdDiag(~ 1 + signal_for_mass_50 + U),
    nestcode_rearing = pdDiag(~ 1 + signal_for_mass_50 + U)
  )
)
save(mass_50_climmod_segmented, file = "output/mass_50_climmod_segmented_22.rda")

mass_50_climmod_segmented <- segmented(mass_50_climlme,
  seg.Z = ~signal_for_mass_50, psi = 23,
  random = list(
    year_f = pdDiag(~ 1 + signal_for_mass_50 + U),
```

```r
    nestcode_rearing = pdDiag(~ 1 + signal_for_mass_50 + U)
  )
)
save(mass_50_climmod_segmented, file = "output/mass_50_climmod_segmented_23.rda")

mass_50_climmod_segmented <- segmented(mass_50_climlme,
  seg.Z = ~signal_for_mass_50, psi = 16,
  random = list(
    year_f = pdDiag(~ 1 + signal_for_mass_50 + U),
    nestcode_rearing = pdDiag(~ 1 + signal_for_mass_50 + U)
  )
)
save(mass_50_climmod_segmented, file = "output/mass_50_climmod_segmented_16.rda")

mass_50_climmod_segmented2 <- segmented(mass_50_climlme,
  seg.Z = ~signal_for_mass_50, psi = c(17, 22),
  random = list(
    year_f = pdDiag(~ 1 + signal_for_mass_50 + U),
    nestcode_rearing = pdDiag(~ 1 + signal_for_mass_50 + U)
  )
)
save(mass_50_climmod_segmented2,
  file = "output/mass_50_climmod_segmented_2-17-22.rda"
)
```

Body mass The analysis was repeated using different starting values (psi) to check if that was affecting the threshold found. Summary of the segmented analysis:

psi = 16

```r
load("output/mass_50_climmod_segmented_16.rda")
summary(mass_50_climmod_segmented)
```

```
## Segmented mixed-effects model fit by REML
##        AIC      BIC    logLik
##   19090.38 19178.82 -9530.191
##  Bootstrap restarting on 10 samples;  6 different solution(s)
##
## Random effects:
##  Formula: ~1 + signal_for_mass_50 + U | year_f
##  Structure: Diagonal
##         (Intercept) signal_for_mass_50        U
## StdDev:    2.513369        0.001395197 1.266925
##
##  Formula: ~1 + signal_for_mass_50 + U | nestcode_rearing %in% year_f
##  Structure: Diagonal
##         (Intercept) signal_for_mass_50          U Residual
## StdDev:    5.292576         0.01473296 0.05008139 6.791907
##
## Fixed effects:
##                         Value Std.Error   DF t-value p-value
## (Intercept)        69.3383    3.7229 1381  18.625  0.0000
## bsh_ax             -2.4003    0.3154 1280  -7.611  0.0000
```

```
## colonySolothurn     2.5634    0.4577 1280   5.601  0.0000
## hatch_doy_sc       -1.5504    0.2994 1381  -5.179  0.0000
## age_days_sc50       0.2206    0.1344 1381   1.642  0.1009
## -- leftS:
## signal_for_mass_50  1.7136    0.2055 1381   8.338  0.0000
## -- diffS:
## U                  -2.3270    0.4935 1381  -4.715
## -- break:
## G0                 19.1590    0.2900 1381
##  psi.link = identity
##
## Standardized Within-Group Residuals:
##         Min          Q1         Med          Q3         Max
## -3.85009835 -0.48382193  0.03584051  0.53958220  4.12865580
##
## Number of Observations: 2693
## Number of Groups:
##                        year_f nestcode_rearing %in% year_f
##                            25                          1307
```

psi = 20

```r
load("output/mass_50_climmod_segmented_20.rda")
summary(mass_50_climmod_segmented)
```

```
## Segmented mixed-effects model fit by REML
##        AIC      BIC    logLik
##   19090.37 19178.81 -9530.185
##  Bootstrap restarting on 10 samples;  3 different solution(s)
##
## Random effects:
##  Formula: ~1 + signal_for_mass_50 + U | year_f
##  Structure: Diagonal
##         (Intercept) signal_for_mass_50        U
## StdDev:    2.515159        0.001408265 1.241755
##
##  Formula: ~1 + signal_for_mass_50 + U | nestcode_rearing %in% year_f
##  Structure: Diagonal
##         (Intercept) signal_for_mass_50          U Residual
## StdDev:    5.292365         0.01483897 0.04884255 6.792138
##
## Fixed effects:
##                        Value Std.Error   DF t-value p-value
## (Intercept)        69.0893    3.7867 1381  18.245  0.0000
## bsh_ax             -2.3988    0.3154 1280  -7.606  0.0000
## colonySolothurn     2.5624    0.4577 1280   5.598  0.0000
## hatch_doy_sc       -1.5507    0.2994 1381  -5.179  0.0000
## age_days_sc50       0.2198    0.1344 1381   1.636  0.1022
## -- leftS:
## signal_for_mass_50  1.7290    0.2097 1381   8.244  0.0000
## -- diffS:
## U                  -2.3166    0.4864 1381  -4.763
## -- break:
```

```
## G0                  19.1349    0.2908 1381
##  psi.link = identity
##
## Standardized Within-Group Residuals:
##         Min          Q1         Med          Q3         Max
## -3.85069006 -0.48266406  0.03708332  0.54051849  4.12827680
##
## Number of Observations: 2693
## Number of Groups:
##                         year_f nestcode_rearing %in% year_f
##                             25                         1307
```

psi = 21

```r
load("output/mass_50_climmod_segmented_21.rda")
summary(mass_50_climmod_segmented)
```

```
## Segmented mixed-effects model fit by REML
##       AIC      BIC    logLik
##   19090.4 19178.83 -9530.198
##  Bootstrap restarting on 10 samples;  2 different solution(s)
##
## Random effects:
##  Formula: ~1 + signal_for_mass_50 + U | year_f
##  Structure: Diagonal
##         (Intercept) signal_for_mass_50        U
## StdDev:     2.52527        0.001413049 1.223453
##
##  Formula: ~1 + signal_for_mass_50 + U | nestcode_rearing %in% year_f
##  Structure: Diagonal
##         (Intercept) signal_for_mass_50          U Residual
## StdDev:    5.292029         0.01488652 0.04727261 6.792072
##
## Fixed effects:
##                         Value Std.Error   DF t-value p-value
## (Intercept)           68.8372    3.8157 1381  18.041  0.0000
## bsh_ax                -2.3970    0.3154 1280  -7.600  0.0000
## colonySolothurn        2.5624    0.4578 1280   5.598  0.0000
## hatch_doy_sc          -1.5508    0.2997 1381  -5.175  0.0000
## age_days_sc50          0.2184    0.1345 1381   1.624  0.1046
## -- leftS:
## signal_for_mass_50     1.7446    0.2117 1381   8.243  0.0000
## -- diffS:
## U                     -2.3077    0.4820 1381  -4.788
## -- break:
## G0                    19.1047    0.2927 1381
##  psi.link = identity
##
## Standardized Within-Group Residuals:
##         Min          Q1         Med          Q3         Max
## -3.85137691 -0.48288586  0.03695215  0.53929450  4.12791184
##
## Number of Observations: 2693
```

```
## Number of Groups:
##                        year_f nestcode_rearing %in% year_f
##                            25                          1307
```

psi = 22

```r
load("output/mass_50_climmod_segmented_22.rda")
summary(mass_50_climmod_segmented)
```

```
## Segmented mixed-effects model fit by REML
##       AIC      BIC    logLik
##   19090.4 19178.83 -9530.198
##  Bootstrap restarting on 10 samples;  2 different solution(s)
##
## Random effects:
##  Formula: ~1 + signal_for_mass_50 + U | year_f
##  Structure: Diagonal
##         (Intercept) signal_for_mass_50        U
## StdDev:     2.52527        0.001413049 1.223453
##
##  Formula: ~1 + signal_for_mass_50 + U | nestcode_rearing %in% year_f
##  Structure: Diagonal
##         (Intercept) signal_for_mass_50          U Residual
## StdDev:    5.292029         0.01488652 0.04727261 6.792072
##
## Fixed effects:
##                        Value Std.Error   DF t-value p-value
## (Intercept)          68.8372    3.8157 1381  18.041  0.0000
## bsh_ax               -2.3970    0.3154 1280  -7.600  0.0000
## colonySolothurn       2.5624    0.4578 1280   5.598  0.0000
## hatch_doy_sc         -1.5508    0.2997 1381  -5.175  0.0000
## age_days_sc50         0.2184    0.1345 1381   1.624  0.1046
## -- leftS:
## signal_for_mass_50  1.7446    0.2117 1381   8.243  0.0000
## -- diffS:
## U                    -2.3077    0.4820 1381  -4.788
## -- break:
## G0                   19.1047    0.2927 1381
##  psi.link = identity
##
## Standardized Within-Group Residuals:
##         Min          Q1         Med          Q3         Max
## -3.85137692 -0.48288586  0.03695216  0.53929450  4.12791184
##
## Number of Observations: 2693
## Number of Groups:
##                        year_f nestcode_rearing %in% year_f
##                            25                          1307
```

psi = 23

```r
load("output/mass_50_climmod_segmented_23.rda")
summary(mass_50_climmod_segmented)
```

```
## Segmented mixed-effects model fit by REML
##        AIC      BIC    logLik
##   19090.37 19178.81 -9530.186
##  Bootstrap restarting on 10 samples;  1 different solution(s)
##
## Random effects:
##  Formula: ~1 + signal_for_mass_50 + U | year_f
##  Structure: Diagonal
##         (Intercept) signal_for_mass_50        U
## StdDev:    2.512856        0.001400537 1.262121
##
##  Formula: ~1 + signal_for_mass_50 + U | nestcode_rearing %in% year_f
##  Structure: Diagonal
##         (Intercept) signal_for_mass_50          U Residual
## StdDev:    5.292569         0.01476722 0.04996561  6.79192
##
## Fixed effects:
##                      Value Std.Error   DF t-value p-value
## (Intercept)        69.2932    3.7285 1381  18.585  0.0000
## bsh_ax             -2.4001    0.3154 1280  -7.610  0.0000
## colonySolothurn     2.5631    0.4577 1280   5.600  0.0000
## hatch_doy_sc       -1.5504    0.2994 1381  -5.179  0.0000
## age_days_sc50       0.2205    0.1344 1381   1.641  0.1011
## -- leftS:
## signal_for_mass_50  1.7164    0.2059 1381   8.336  0.0000
## -- diffS:
## U                  -2.3252    0.4927 1381  -4.719
## -- break:
## G0                 19.1538    0.2872 1381
##  psi.link = identity
##
## Standardized Within-Group Residuals:
##         Min          Q1         Med          Q3         Max
## -3.85021117 -0.48336447  0.03608181  0.53985955  4.12859034
##
## Number of Observations: 2693
## Number of Groups:
##                       year_f nestcode_rearing %in% year_f
##                           25                         1307
```

psi = 19

```r
load("output/mass_50_climmod_segmented_19.rda")
summary(mass_50_climmod_segmented)
```

```
## Segmented mixed-effects model fit by REML
##       AIC      BIC    logLik
##   19090.4 19178.83 -9530.198
##  Bootstrap restarting on 10 samples;  2 different solution(s)
```

```
##
## Random effects:
##  Formula: ~1 + signal_for_mass_50 + U | year_f
##  Structure: Diagonal
##         (Intercept) signal_for_mass_50        U
## StdDev:    2.525282        0.001413091 1.223397
##
##  Formula: ~1 + signal_for_mass_50 + U | nestcode_rearing %in% year_f
##  Structure: Diagonal
##         (Intercept) signal_for_mass_50          U Residual
## StdDev:    5.292029         0.01488681 0.04726972 6.792072
##
## Fixed effects:
##                       Value Std.Error   DF t-value p-value
## (Intercept)        68.8366    3.8157 1381  18.040  0.0000
## bsh_ax             -2.3970    0.3154 1280  -7.600  0.0000
## colonySolothurn     2.5624    0.4578 1280   5.598  0.0000
## hatch_doy_sc       -1.5508    0.2997 1381  -5.175  0.0000
## age_days_sc50       0.2184    0.1345 1381   1.624  0.1046
## -- leftS:
## signal_for_mass_50  1.7446    0.2117 1381   8.243  0.0000
## -- diffS:
## U                  -2.3077    0.4819 1381  -4.788
## -- break:
## G0                 19.1046    0.2927 1381
##  psi.link = identity
##
## Standardized Within-Group Residuals:
##         Min          Q1         Med          Q3         Max
## -3.85137870 -0.48288665  0.03695542  0.53929255  4.12791094
##
## Number of Observations: 2693
## Number of Groups:
##                       year_f nestcode_rearing %in% year_f
##                           25                         1307
```

All the threshold found are very similar, this we decided to use the threshold identified by this last model.

The threshold is at 19.1531402 according to the segmented analysis. The two slopes (before and after the point) are:

```r
segmented_threshold <- mean(mass_50_climmod_segmented$psi)

data_am_nestlings_mass_501 <- subset(
  data_am_nestlings_mass_50,
  signal_for_mass_50 < segmented_threshold
)
data_am_nestlings_mass_502 <- subset(
  data_am_nestlings_mass_50,
  signal_for_mass_50 > segmented_threshold
)
```

Before the point

```r
mass_50_climmod1 <- lmer(
  mass_50 ~
    signal_for_mass_50 +
    brood_size + colony +
    hatch_doy_sc + age_days_sc50 +
    (1 | year_f) + (1 | nestcode_rearing),
  control = lmerControl(optimizer = "bobyqa", optCtrl = list(maxfun = 1e+06)),
  data = data_am_nestlings_mass_501
)
summary(mass_50_climmod1)
```

```
## Linear mixed model fit by REML. t-tests use Satterthwaite's method ['lmerModLmerTest']
## Formula: mass_50 ~ signal_for_mass_50 + brood_size + colony + hatch_doy_sc +
##     age_days_sc50 + (1 | year_f) + (1 | nestcode_rearing)
##    Data: data_am_nestlings_mass_501
## Control: lmerControl(optimizer = "bobyqa", optCtrl = list(maxfun = 1e+06))
##
## REML criterion at convergence: 10315.7
##
## Scaled residuals:
##     Min      1Q  Median      3Q     Max
## -3.7567 -0.4819  0.0292  0.5206  4.0466
##
## Random effects:
##  Groups           Name        Variance Std.Dev.
##  nestcode_rearing (Intercept) 32.330   5.686
##  year_f           (Intercept)  7.895   2.810
##  Residual                     48.426   6.959
## Number of obs: 1443, groups:  nestcode_rearing, 715; year_f, 25
##
## Fixed effects:
##                    Estimate Std. Error       df t value Pr(>|t|)
## (Intercept)         69.4941     4.5005 387.7888  15.442  < 2e-16 ***
## signal_for_mass_50   1.7663     0.2474 470.9707   7.139 3.58e-12 ***
## brood_size          -2.9802     0.4531 805.4378  -6.577 8.64e-11 ***
## colonySolothurn      3.2735     0.6450 655.8994   5.076 5.04e-07 ***
## hatch_doy_sc        -1.2887     0.4053 146.5579  -3.180   0.0018 **
## age_days_sc50        0.3164     0.1788 713.6018   1.770   0.0772 .
## ---
## Signif. codes:  0 '***' 0.001 '**' 0.01 '*' 0.05 '.' 0.1 ' ' 1
##
## Correlation of Fixed Effects:
##             (Intr) s___50 brd_sz clnySl htch__
## sgnl_fr__50 -0.950
## brood_size  -0.239 -0.014
## colnySlthrn -0.079 -0.007 -0.054
## hatch_dy_sc -0.197  0.134  0.138  0.094
## ag_dys_sc50 -0.009  0.049 -0.123 -0.108 -0.033
```

```r
car::Anova(mass_50_climmod1)
```

```
## Analysis of Deviance Table (Type II Wald chisquare tests)
```

```
##
## Response: mass_50
##                    Chisq Df Pr(>Chisq)
## signal_for_mass_50 50.9685  1  9.386e-13 ***
## brood_size         43.2537  1  4.808e-11 ***
## colony             25.7608  1  3.865e-07 ***
## hatch_doy_sc       10.1116  1   0.001473 **
## age_days_sc50       3.1329  1   0.076728 .
## ---
## Signif. codes:  0 '***' 0.001 '**' 0.01 '*' 0.05 '.' 0.1 ' ' 1
```

After the point

```
mass_50_climmod2 <- lmer(
  mass_50 ~
    signal_for_mass_50 +
    # I(signal_for_mass_50^2) +
    brood_size + colony +
    hatch_doy_sc + age_days_sc50 +
    (1 | year_f) + (1 | nestcode_rearing),
  control = lmerControl(optimizer = "bobyqa", optCtrl = list(maxfun = 1e+06)),
  data = data_am_nestlings_mass_502
)
summary(mass_50_climmod2)
```

```
## Linear mixed model fit by REML. t-tests use Satterthwaite's method ['lmerModLmerTest']
## Formula: mass_50 ~ signal_for_mass_50 + brood_size + colony + hatch_doy_sc +
##     age_days_sc50 + (1 | year_f) + (1 | nestcode_rearing)
##    Data: data_am_nestlings_mass_502
## Control: lmerControl(optimizer = "bobyqa", optCtrl = list(maxfun = 1e+06))
##
## REML criterion at convergence: 8717.9
##
## Scaled residuals:
##     Min      1Q  Median      3Q     Max
## -3.6675 -0.4777  0.0479  0.5484  2.6921
##
## Random effects:
##  Groups           Name        Variance Std.Dev.
##  nestcode_rearing (Intercept) 25.133   5.013
##  year_f           (Intercept)  1.622   1.274
##  Residual                     42.829   6.544
## Number of obs: 1250, groups:  nestcode_rearing, 597; year_f, 24
##
## Fixed effects:
##                     Estimate Std. Error        df t value Pr(>|t|)
## (Intercept)        110.12453    5.05255 204.70678  21.796  < 2e-16 ***
## signal_for_mass_50  -0.47951    0.23259 205.55329  -2.062 0.040504 *
## brood_size          -1.88957    0.43565 745.90016  -4.337 1.64e-05 ***
## colonySolothurn      2.25863    0.62198 442.01146   3.631 0.000315 ***
## hatch_doy_sc        -1.91266    0.36992  71.14396  -5.170 2.06e-06 ***
## age_days_sc50       -0.07131    0.20929 557.30963  -0.341 0.733429
## ---
```

```
## Signif. codes:  0 '***' 0.001 '**' 0.01 '*' 0.05 '.' 0.1 ' ' 1
##
## Correlation of Fixed Effects:
##             (Intr) s___50 brd_sz clnySl htch__
## sgnl_fr__50 -0.968
## brood_size  -0.204 -0.021
## colnySlthrn -0.041 -0.029 -0.049
## hatch_dy_sc -0.044  0.034  0.066 -0.028
## ag_dys_sc50 -0.012  0.042 -0.024 -0.223  0.058
```

```
car::Anova(mass_50_climmod2)
```

```
## Analysis of Deviance Table (Type II Wald chisquare tests)
##
## Response: mass_50
##                      Chisq Df Pr(>Chisq)
## signal_for_mass_50  4.2502  1  0.0392456 *
## brood_size         18.8125  1  1.442e-05 ***
## colony             13.1869  1  0.0002819 ***
## hatch_doy_sc       26.7339  1  2.335e-07 ***
## age_days_sc50       0.1161  1  0.7333002
## ---
## Signif. codes:  0 '***' 0.001 '**' 0.01 '*' 0.05 '.' 0.1 ' ' 1
```

```
clim_predict <-
  data.frame(effects::effect("signal_for_mass_50",
    mass_50_climmod,
    partial.residuals = TRUE
  ))
clim_predict_01 <-
  data.frame(effects::effect("signal_for_mass_50",
    mass_50_climmod1,
    partial.residuals = TRUE
  ))
clim_predict_02 <-
  data.frame(effects::effect("signal_for_mass_50",
    mass_50_climmod2,
    partial.residuals = TRUE
  ))



par_xmin <- round(min(data_am_nestlings_mass_50$signal_for_mass_50) - 0.005, 2)
par_xmax <- round(max(data_am_nestlings_mass_50$signal_for_mass_50) + 0.005, 2)


plot_clim_mass_50 <- ggplot(
  clim_predict,
  aes(signal_for_mass_50, fit)
) +
  geom_point(
    data = data_am_nestlings_mass_50,
    aes(
```

```
      x = signal_for_mass_50,
      y = mass_50
    ),
    col = par_col_mass_50,
    size = 1, shape = 16, alpha = 0.2
  ) +
  geom_vline(
    xintercept = segmented_threshold,
    linetype = "dashed",
    color = "dodgerblue"
  ) +
  geom_ribbon(
    data = clim_predict_01,
    aes(ymin = lower, ymax = upper),
    alpha = .25
  ) +
  geom_line(
    data = clim_predict_01, aes(signal_for_mass_50, fit),
    col = "darkgreen",
    linewidth = 1.2
  ) +
  geom_ribbon(
    data = clim_predict_02,
    aes(ymin = lower, ymax = upper),
    alpha = .25
  ) +
  geom_line(
    data = clim_predict_02, aes(signal_for_mass_50, fit),
    col = "#20b2aa",
    linewidth = 1.2
  ) +
  theme(
    legend.position = "none",
    panel.grid.major = element_blank(),
    panel.grid.minor = element_blank(),
    panel.background = element_blank(),
    axis.line = element_line(colour = "black"),
    axis.title.x = element_markdown()
  ) +
  xlab(
    "Mean ambient temperature (Ta<sub>48-50d</sub>; °C)<br>between 48 and 50 days of age"
  ) +
  ylab("Body mass at 50 days (g)") +
  scale_y_continuous(
    limits = c(55, 130),
    breaks = c(seq(55, 150, 15))
  ) +
  scale_x_continuous(
    limits = c(par_xmin, par_xmax),
    breaks = c(round(seq(
      par_xmin, par_xmax,
      (par_xmax - par_xmin) / 4
    ), 1))
```

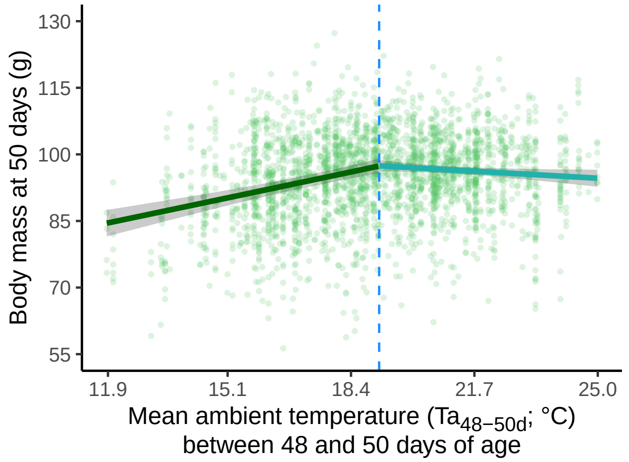

Figure 4: Fig. 3 - Variation in body mass of 50-day-old nestling Alpine swifts in relation to daily average temperature in the 2 days before the measurement. The analysis using the segmented package identified a threshold at 19.1°C. Solid lines (and 95% confidence intervals) are predictions from the models presented in Table 2. Climatic windows are reported using the nestling's age as a reference.

```
  )
```

Figure

```
plot_clim_mass_50
```

```
# Save the plot to the figures directory
ggsave("figures/Fig. 3 - mass-weather_segmented.png", width = 4, height = 3, dpi = 300)
```

Supporting Information 02: Growth rates

To estimate growth rates, I extracted the slope of the linear regression between the measurements of the trait within the windows identified by the climwin analyses.

```
# Growth rates  ----------------------------------------------------------------

am_growth_wing <- data_am_nestlings_all %>%
  subset(!is.na(wing)) %>%
  subset(age_days <= 48) %>%
  subset(age_days >= 1) %>%
  group_by(ring) %>%
  mutate(
    wing_n_measures = n()
  ) %>%
  ungroup()

am_growth_wing <- am_growth_wing %>%
  subset(!is.na(wing_n_measures) & wing_n_measures > 2) %>%
  dplyr::select(ring, wing, age_days, wing_n_measures) %>%
```

```r
  distinct() %>%
  group_by(ring) %>%
  mutate(
    wing_growth = coefficients(lm(wing ~ age_days))[2],
    wing_r2 = summary(lm(wing ~ age_days))$r.squared
  ) %>%
  ungroup() %>%
  dplyr::select(
    ring, wing_growth, wing_n_measures, wing_r2
  ) %>%
  distinct()
```

```
## Warning: There were 6 warnings in `mutate()`.
## The first warning was:
## i In argument: `wing_r2 = summary(lm(wing ~ age_days))$r.squared`.
## i In group 61: `ring = ring_000074`.
## Caused by warning in `summary.lm()`:
## ! essentially perfect fit: summary may be unreliable
## i Run `dplyr::last_dplyr_warnings()` to see the 5 remaining warnings.
```

```r
am_growth_sternum <- data_am_nestlings_all %>%
  subset(!is.na(sternum)) %>%
  subset(age_days <= 34) %>%
  subset(age_days >= 12) %>%
  group_by(ring) %>%
  mutate(
    sternum_n_measures = n()
  ) %>%
  ungroup()

am_growth_sternum <- am_growth_sternum %>%
  subset(!is.na(sternum_n_measures) & sternum_n_measures >= 2) %>%
  dplyr::select(ring, sternum, age_days, sternum_n_measures) %>%
  distinct() %>%
  group_by(ring) %>%
  mutate(
    sternum_growth = coefficients(lm(sternum ~ age_days))[2],
    sternum_r2 = summary(lm(sternum ~ age_days))$r.squared
  ) %>%
  ungroup() %>%
  dplyr::select(
    ring, sternum_growth, sternum_n_measures, sternum_r2
  ) %>%
  distinct()
```

```
## Warning: There were 3 warnings in `mutate()`.
## The first warning was:
## i In argument: `sternum_r2 = summary(lm(sternum ~ age_days))$r.squared`.
## i In group 620: `ring = ring_000987`.
## Caused by warning in `summary.lm()`:
## ! essentially perfect fit: summary may be unreliable
## i Run `dplyr::last_dplyr_warnings()` to see the 2 remaining warnings.
```

```r
# adding the growth rates to the dataset
am_nestlings_clim <- merge(am_nestlings_clim,
  merge(am_growth_wing, am_growth_sternum, all = TRUE),
  all.x = TRUE
)

rm(am_growth_wing, am_growth_sternum)

# creating the subsets for the analyses without NAs
data_am_nestlings_wing_growth <- subset(
  am_nestlings_clim, !is.na(wing_growth)
)
data_am_nestlings_sternum_growth <- subset(
  am_nestlings_clim, !is.na(sternum_growth)
)
```

The warnings in this case just indicate that in some cases we have perfect fit, as it's quite likely with fitting a linear model using 2-3 points.

Checking the resulting growth rates

Wing growth rate

The histograms of the R squared values are plotted here.

```r
hist(data_am_nestlings_wing_growth$wing_r2, breaks = 50)
```

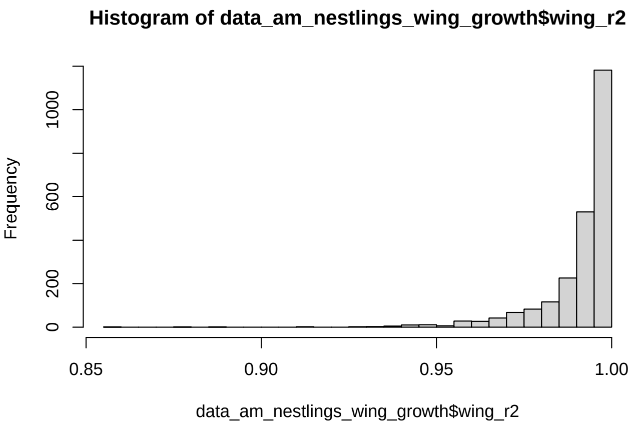

The r2 value for the wing growth rates are 0.99 + 0.01 (mean + SD). The histogram and the average values show that the linear fit is a good approximation of the growth of the nestlings within the time window considered.

Sternum growth rate

The histograms of the R squared values are plotted here.

```r
hist(data_am_nestlings_sternum_growth$sternum_r2, breaks = 50)
```

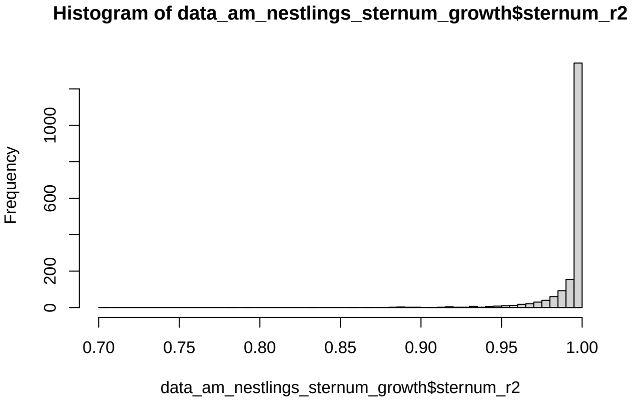

As for the sternum we constructed the slopes for nestlings with at least 2 measurements within the window, here we show also the R squared for nestlings with at least 3 measurements within the window to check that the R squared obtained are still good.

```
hist(subset(
  data_am_nestlings_sternum_growth,
  sternum_n_measures > 2
)$sternum_r2, breaks = 50)
```

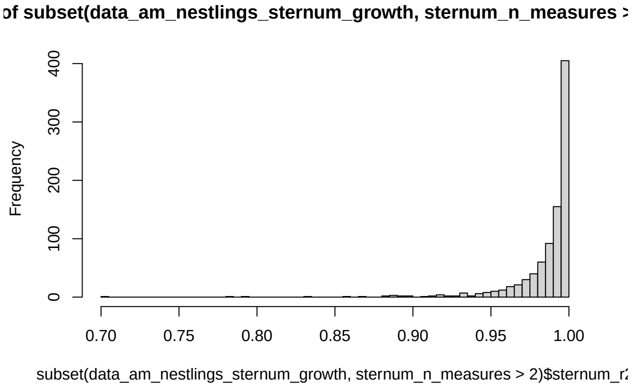

This shows that using a linear slope for the sternum is a good approximation even fore nestlings with only 2 measurements.

The r2 value for the sternum growth rates are 0.99 + 0.02 (mean + SD). The histograms and the average values show that the linear fit is a good approximation of the growth of the nestlings within the time window considered.

Growth rate models

Wing length

```
wing_growth_climmod_quad <- lmer(
  wing_growth ~
    signal_for_wing_50 +
    I(signal_for_wing_50^2) +
    brood_size + colony +
    hatch_doy_sc +
    (1 | year_f) + (1 | nestcode_rearing),
```

```r
  control = lmerControl(optimizer = "bobyqa", optCtrl = list(maxfun = 1e+06)),
  data = data_am_nestlings_wing_growth
)
wing_growth_climmod_lin <- lmer(
  wing_growth ~
    signal_for_wing_50 +
    brood_size + colony +
    hatch_doy_sc +
    (1 | year_f) + (1 | nestcode_rearing),
  control = lmerControl(optimizer = "bobyqa", optCtrl = list(maxfun = 1e+06)),
  data = data_am_nestlings_wing_growth
)
MuMIn::AICc(wing_growth_climmod_quad, wing_growth_climmod_lin)
```

```
##                          df     AICc
## wing_growth_climmod_quad  9 1534.603
## wing_growth_climmod_lin   8 1533.470
```

The AIC value is very similar but linear is lower and more parsimonious.

Sternum length

```r
sternum_growth_climmod_quad <- lmer(
  sternum_growth ~
    signal_for_sternum_50 + I(signal_for_sternum_50^2) +
    brood_size + colony +
    hatch_doy_sc +
    (1 | year_f) + (1 | nestcode_rearing),
  control = lmerControl(optimizer = "bobyqa", optCtrl = list(maxfun = 1e+06)),
  data = data_am_nestlings_sternum_growth
)
sternum_growth_climmod_lin <- lmer(
  sternum_growth ~
    signal_for_sternum_50 +
    brood_size + colony +
    hatch_doy_sc +
    (1 | year_f) + (1 | nestcode_rearing),
  control = lmerControl(optimizer = "bobyqa", optCtrl = list(maxfun = 1e+06)),
  data = data_am_nestlings_sternum_growth
)

MuMIn::AICc(sternum_growth_climmod_quad, sternum_growth_climmod_lin)
```

```
##                             df      AICc
## sternum_growth_climmod_quad  9 -2191.962
## sternum_growth_climmod_lin   8 -2197.919
```

```r
# quadratic seems better
```

The linear model has a lower AIC value.

```r
wing_growth_climmod <- lmer(
  wing_growth ~
    brood_size + colony +
    hatch_doy_sc +
    signal_for_wing_50 +
    (1 | year_f) + (1 | nestcode_rearing),
  control = lmerControl(optimizer = "bobyqa", optCtrl = list(maxfun = 1e+06)),
  data = data_am_nestlings_wing_growth
)
summary(wing_growth_climmod)
```

```
## Linear mixed model fit by REML. t-tests use Satterthwaite's method ['lmerModLmerTest']
## Formula: wing_growth ~ brood_size + colony + hatch_doy_sc + signal_for_wing_50 +
##     (1 | year_f) + (1 | nestcode_rearing)
##    Data: data_am_nestlings_wing_growth
## Control: lmerControl(optimizer = "bobyqa", optCtrl = list(maxfun = 1e+06))
##
## REML criterion at convergence: 1517.4
##
## Scaled residuals:
##     Min      1Q  Median      3Q     Max
## -7.5306 -0.3636  0.0996  0.4841  4.0765
##
## Random effects:
##  Groups           Name        Variance Std.Dev.
##  nestcode_rearing (Intercept) 0.05580  0.2362
##  year_f           (Intercept) 0.09736  0.3120
##  Residual                     0.06619  0.2573
## Number of obs: 2344, groups:  nestcode_rearing, 1141; year_f, 25
##
## Fixed effects:
##                      Estimate Std. Error         df t value Pr(>|t|)
## (Intercept)           4.97208    0.07832   46.68061  63.484  < 2e-16 ***
## brood_size           -0.13328    0.01391 1276.31114  -9.584  < 2e-16 ***
## colonySolothurn       0.07707    0.02183 1074.50792   3.530 0.000434 ***
## hatch_doy_sc         -0.14156    0.01417 1104.04962  -9.992  < 2e-16 ***
## signal_for_wing_50    0.12872    0.08637  382.29280   1.490 0.136971
## ---
## Signif. codes:  0 '***' 0.001 '**' 0.01 '*' 0.05 '.' 0.1 ' ' 1
##
## Correlation of Fixed Effects:
##             (Intr) brd_sz clnySl htch__
## brood_size  -0.437
## colnySlthrn -0.154 -0.077
## hatch_dy_sc -0.126  0.131  0.083
## sgnl_fr__50 -0.328 -0.017 -0.062  0.110
```

```r
sternum_growth_climmod <- lmer(
  sternum_growth ~
    brood_size + colony +
    hatch_doy_sc +
    signal_for_sternum_50 +
    (1 | year_f) + (1 | nestcode_rearing),
```

```r
  control = lmerControl(optimizer = "bobyqa", optCtrl = list(maxfun = 1e+06)),
  data = data_am_nestlings_sternum_growth
)
summary(sternum_growth_climmod)
```

```
## Linear mixed model fit by REML. t-tests use Satterthwaite's method ['lmerModLmerTest']
## Formula: sternum_growth ~ brood_size + colony + hatch_doy_sc + signal_for_sternum_50 +
##     (1 | year_f) + (1 | nestcode_rearing)
##    Data: data_am_nestlings_sternum_growth
## Control: lmerControl(optimizer = "bobyqa", optCtrl = list(maxfun = 1e+06))
##
## REML criterion at convergence: -2214
##
## Scaled residuals:
##     Min      1Q  Median      3Q     Max
## -5.2793 -0.4484  0.0261  0.4572 10.2427
##
## Random effects:
##  Groups           Name        Variance Std.Dev.
##  nestcode_rearing (Intercept) 0.007301 0.08545
##  year_f           (Intercept) 0.011989 0.10949
##  Residual                     0.010893 0.10437
## Number of obs: 1824, groups:  nestcode_rearing, 893; year_f, 21
##
## Fixed effects:
##                         Estimate Std. Error         df t value Pr(>|t|)
## (Intercept)            9.130e-01  3.132e-02  4.855e+01  29.149  < 2e-16 ***
## brood_size            -2.782e-02  5.829e-03  1.038e+03  -4.773 2.08e-06 ***
## colonySolothurn       -1.585e-03  9.607e-03  8.725e+02  -0.165  0.86897
## hatch_doy_sc          -1.810e-02  6.120e-03  8.544e+02  -2.958  0.00318 **
## signal_for_sternum_50  8.008e-02  2.714e-02  3.429e+02   2.951  0.00339 **
## ---
## Signif. codes:  0 '***' 0.001 '**' 0.01 '*' 0.05 '.' 0.1 ' ' 1
##
## Correlation of Fixed Effects:
##             (Intr) brd_sz clnySl htch__
## brood_size  -0.488
## colnySlthrn -0.224 -0.061
## hatch_dy_sc -0.010  0.092  0.078
## sgnl_fr__50 -0.356  0.070  0.046 -0.206
```

```r
labels <- c(
  `(Intercept)` = "Intercept",
  hatch_doy_sc = "Hatching day",
  brood_size = "Hatching brood size",
  colonySolothurn = "Colony [Solothurn]",
  signal_for_wing_50 = "PC1 (age: 1-48)",
  `I(signal_for_wing_50^2)` = "PC1 (age: 1-48) 2",
  signal_for_sternum_50 = "PC1 (age: 12-34)",
  `I(signal_for_sternum_50^2)` = "PC1 (age: 12-34) 2"
)

sjPlot::tab_model(
```

```
  wing_growth_climmod,
  sternum_growth_climmod,
  file = "output/Tab_wicm_models_growth.doc",
  string.est = "Estimate",
  string.se = "SE",
  show.ci = FALSE,
  show.se = TRUE,
  show.stat = FALSE,
  show.df = FALSE,
  pred.labels = labels
)
```

Figure

```
cowplot::plot_grid(
  fun_climres(wing_growth_climmod, data_am_nestlings_wing_growth),
  fun_climres(sternum_growth_climmod, data_am_nestlings_sternum_growth),
  nrow = 2, align = c("v", "h")
)
```

```
# Save the plot to the figures directory
ggsave("figures/Fig. 4 - growths-weather.png", width = 4, height = 5, dpi = 300)
```

Supporting Information 03: Correlation between the size and growth

Figure

Here we show the correlation between the size at 50 days (wing, sternum and mass) and the growth rates (wing and sternum).

```
PerformanceAnalytics::chart.Correlation(am_nestlings_clim[
  ,
  c("wing_50", "wing_growth", "sternum_50", "sternum_growth", "mass_50")
], histogram = TRUE, pch = 19, method = "pearson")
```

```
## Warning in par(usr): argument 1 does not name a graphical parameter
## Warning in par(usr): argument 1 does not name a graphical parameter
## Warning in par(usr): argument 1 does not name a graphical parameter
## Warning in par(usr): argument 1 does not name a graphical parameter
## Warning in par(usr): argument 1 does not name a graphical parameter
## Warning in par(usr): argument 1 does not name a graphical parameter
## Warning in par(usr): argument 1 does not name a graphical parameter
## Warning in par(usr): argument 1 does not name a graphical parameter
## Warning in par(usr): argument 1 does not name a graphical parameter
## Warning in par(usr): argument 1 does not name a graphical parameter
```

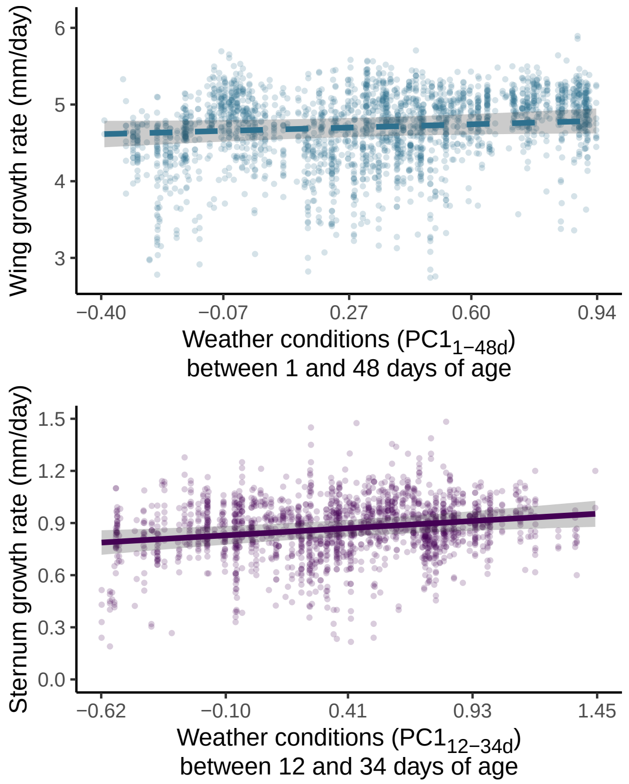

Figure 5: Fig. 4 - Variation in wing and sternum growth rates of nestling Alpine swifts in relation to weather conditions encountered during development. Weather conditions resulted from a principal component analysis (PC1) between daily average temperature and daily rain, and traits were affected by weather conditions over different time windows. PC1 has low values with cold and rainy days and high values with sunny and warm days. Solid lines (and 95% confidence intervals) are predictions from the models presented in Table 3. Climatic windows are reported using the nestling's age as a reference.

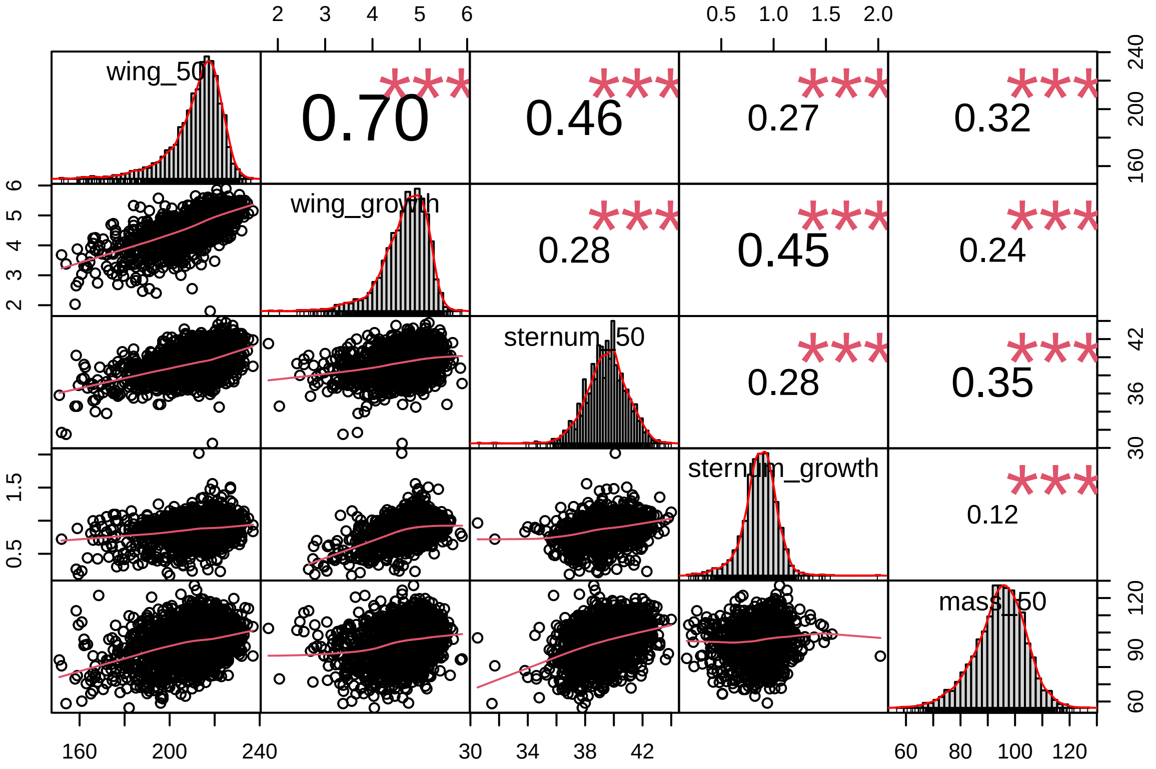

In the above plot:

- The distribution of each variable is shown on the diagonal.
- On the bottom of the diagonal: the bivariate scatter plots with a fitted line are displayed
- On the top of the diagonal: the value of the correlation (Pearson r) plus the significance level as stars
- Each significance level (p-values: 0, 0.001, 0.01, 0.05, 0.1, 1) is associated to a symbol.

Pearson's correlation tests

Wing length

```
cor.test(am_nestlings_clim$wing_50, am_nestlings_clim$wing_growth)
```

```
##
##  Pearson's product-moment correlation
##
## data:  am_nestlings_clim$wing_50 and am_nestlings_clim$wing_growth
## t = 47.217, df = 2342, p-value < 2.2e-16
## alternative hypothesis: true correlation is not equal to 0
## 95 percent confidence interval:
##  0.6770023 0.7185188
## sample estimates:
##       cor
## 0.6983475
```

Sternum length

```r
cor.test(am_nestlings_clim$sternum_50, am_nestlings_clim$sternum_growth)
```

```
##
##  Pearson's product-moment correlation
##
## data:  am_nestlings_clim$sternum_50 and am_nestlings_clim$sternum_growth
## t = 12.544, df = 1822, p-value < 2.2e-16
## alternative hypothesis: true correlation is not equal to 0
## 95 percent confidence interval:
##  0.2391484 0.3236600
## sample estimates:
##      cor
## 0.281951
```

Supporting Information 04: Short-term consequences

Probability of fledging

To investigate the number of nestlings that survived I use cbind() with the number of nestlings that fledged and the number of nestlings that did not fledge (number of hatchlings - number of fledglings) using the data from nests with at least 1 nestling fledging.

```r
data_am_nests$hatch_date <- as.Date(data_am_nests$hatch_date)
data_am_nests$date_50days <- data_am_nests$hatch_date + 50
data_am_nests$hatch_doy_sc <- fun_scale(yday(data_am_nests$hatch_date))
data_am_nests$year_f <- data_am_nests$year


data_am_nests <- droplevels(data_am_nests)
data_am_nests$prop <- data_am_nests$fledged /
  (data_am_nests$fledged + data_am_nests$not_fledg)

data_signals <- am_nestlings_clim[c(
  "date_50days", "signal_for_wing_50",
  "signal_for_sternum_50", "signal_for_mass_50"
)] %>%
  distinct(date_50days, .keep_all = TRUE)

data_am_nests <- merge(
  data_am_nests,
  data_signals,
  all.x = TRUE, all.y = FALSE
)
data_am_nests$obs <- seq_len(nrow(data_am_nests))
```

I'm interested in comparing the weather variables that affect each of the trait, to see which is the window that best affects the probability of fledging. To be able to compare the 3 models I first create a subset that has complete cases for all 3 weather conditions.

```r
am_nest_clim_reduced <- subset(subset(
  subset(data_am_nests, !is.na(signal_for_mass_50)),
  !is.na(signal_for_wing_50)
), !is.na(signal_for_sternum_50))

am_nest_clim_wing <- subset(data_am_nests, !is.na(signal_for_wing_50))

fledg_surv_climmod_wing <- glmer(
  cbind(fledged, not_fledg) ~
    brood_size +
    colony +
    hatch_doy_sc +
    signal_for_wing_50 +
    (1 | year_f) +
    (1 | obs),
  data = am_nest_clim_reduced,
  family = binomial,
  control = glmerControl(optimizer = "bobyqa", optCtrl = list(maxfun = 1e+06))
)

fledg_surv_climmod_sternum <- glmer(
  cbind(fledged, not_fledg) ~
    brood_size +
    colony +
    hatch_doy_sc +
    signal_for_sternum_50 +
    (1 | year_f) +
    (1 | obs),
  data = am_nest_clim_reduced,
  family = binomial,
  control = glmerControl(optimizer = "bobyqa", optCtrl = list(maxfun = 1e+06))
)

fledg_surv_climmod_mass <- glmer(
  cbind(fledged, not_fledg) ~
    brood_size +
    colony +
    hatch_doy_sc +
    signal_for_mass_50 +
    (1 | year_f) +
    (1 | obs),
  data = am_nest_clim_reduced,
  family = binomial,
  control = glmerControl(optimizer = "bobyqa", optCtrl = list(maxfun = 1e+06))
)

MuMIn::AICc(
  fledg_surv_climmod_wing,
  fledg_surv_climmod_sternum,
  fledg_surv_climmod_mass
)
```

```
##                              df     AICc
```

```
## fledg_surv_climmod_wing     7 2696.659
## fledg_surv_climmod_sternum  7 2699.054
## fledg_surv_climmod_mass     7 2698.325
```

The climatic window for the sternum length is better even if the models have very similar AIC (deltaAICc < 4). To keep a larger sample size and to be consistent with the following analysis we decided to use the one for the wing length. The window for sternum length is anyway fully included in the window for wing length.

Testing for quadratic effects

```
fledg_surv_climmod_quad <- glmer(
  cbind(fledged, not_fledg) ~
    brood_size +
    colony +
    hatch_doy_sc +
    signal_for_wing_50 +
    I(signal_for_wing_50^2) +
    (1 | year_f) +
    (1 | obs),
  data = am_nest_clim_wing,
  family = binomial,
  control = glmerControl(optimizer = "bobyqa", optCtrl = list(maxfun = 1e+06))
)
fledg_surv_climmod_lin <- glmer(
  cbind(fledged, not_fledg) ~
    brood_size +
    colony +
    hatch_doy_sc +
    signal_for_wing_50 +
    (1 | year_f) +
    (1 | obs),
  data = am_nest_clim_wing,
  family = binomial,
  control = glmerControl(optimizer = "bobyqa", optCtrl = list(maxfun = 1e+06))
)
MuMIn::AICc(fledg_surv_climmod_lin, fledg_surv_climmod_quad)
```

```
##                          df     AICc
## fledg_surv_climmod_lin   7 3020.567
## fledg_surv_climmod_quad  8 3022.307
```

Not keeping the quadratic effect

Checking for overdispersion The models above showed overdispersion when I did not included an observation-level random effect. See an example of the results on overdispersion here:

```
fledg_surv_climmod_lin_overdispersed <- glmer(
  cbind(fledged, not_fledg) ~
    brood_size +
    colony +
    hatch_doy_sc +
    signal_for_wing_50 +
    (1 | year_f),
```

```r
  data = am_nest_clim_wing,
  family = binomial,
  control = glmerControl(optimizer = "bobyqa", optCtrl = list(maxfun = 1e+06))
)
performance::check_overdispersion(fledg_surv_climmod_lin_overdispersed)
```

```
## # Overdispersion test
##
##  dispersion ratio = 1.178
##           p-value = 0.008

## Overdispersion detected.
```

Therefore in all the models on fledgling survival presented above I have included an observation level random effect that deals with the overdispersion of the data. See new results here:

```r
performance::check_overdispersion(fledg_surv_climmod_lin)
```

```
## # Overdispersion test
##
##  dispersion ratio = 1.055
##           p-value = 0.336

## No overdispersion detected.
```

Now there seem to be no overdispersion detected.

Age at fledging

For some years we have data on the age at fledging, so we can check the correlation between age at fledging, size and growth of nestlings

Correlation plot, plus I've tried some analyses, but they are VERY preliminary and need to be checked.

```r
am_age_fledging <- am_nestlings_clim %>%
  subset(!duplicated(ring)) %>%
  subset(!is.na(age_fledging)) %>%
  # subset(!is.na(sternum_50)) %>%
  # subset(year < 2012) %>%
  dplyr::select(
    year, ring, age_fledging,
    brood_size, colony, hatch_doy_sc,
    date_50days,
    wing_50, wing_growth,
    sternum_50, sternum_growth,
    mass_50,
    year_f, nestcode_rearing,
    signal_for_mass_50, signal_for_wing_50,
    signal_for_sternum_50
  )
```

```
am_age_fledging <- am_age_fledging %>%
  filter(year != 2012) %>%
  droplevels()

am_age_fledging_reduced <- am_age_fledging %>%
  filter(!is.na(signal_for_mass_50)) %>%
  filter(!is.na(signal_for_wing_50)) %>%
  filter(!is.na(signal_for_sternum_50))

am_age_fledging_wing <- am_age_fledging %>%
  filter(!is.na(signal_for_wing_50))


age_fledg_climmod_wing <- lmer(
  age_fledging ~
    signal_for_wing_50 +
    brood_size +
    colony +
    hatch_doy_sc +
    (1 | year_f) + (1 | nestcode_rearing),
  data = am_age_fledging_reduced,
  control = lmerControl(
    optimizer = "bobyqa",
    optCtrl = list(maxfun = 1e+07)
  )
)

age_fledg_climmod_sternum <- lmer(
  age_fledging ~
    signal_for_sternum_50 +
    brood_size +
    colony +
    hatch_doy_sc +
    (1 | year_f) + (1 | nestcode_rearing),
  data = am_age_fledging_reduced,
  control = lmerControl(
    optimizer = "bobyqa",
    optCtrl = list(maxfun = 1e+07)
  )
)

age_fledg_climmod_mass <- lmer(
  age_fledging ~
    signal_for_mass_50 +
    brood_size +
    colony +
    hatch_doy_sc +
    (1 | year_f) + (1 | nestcode_rearing),
  data = am_age_fledging_reduced,
  control = lmerControl(
    optimizer = "bobyqa",
    optCtrl = list(maxfun = 1e+07)
  )
```

```
)

MuMIn::AICc(age_fledg_climmod_wing, age_fledg_climmod_sternum, age_fledg_climmod_mass)
```

```
##                           df     AICc
## age_fledg_climmod_wing     8 2936.399
## age_fledg_climmod_sternum  8 2948.733
## age_fledg_climmod_mass     8 2940.965
```

The climatic window for the wings is the best one.

Testing for quadratic effects

```
age_fledg_climmod_quad <- lmer(
  age_fledging ~
    signal_for_wing_50 +
    I(signal_for_wing_50^2) +
    brood_size +
    colony +
    hatch_doy_sc +
    (1 | year_f) + (1 | nestcode_rearing),
  data = am_age_fledging_wing,
  control = lmerControl(
    optimizer = "bobyqa",
    optCtrl = list(maxfun = 1e+07)
  )
)

age_fledg_climmod_lin <- lmer(
  age_fledging ~
    signal_for_wing_50 +
    brood_size +
    colony +
    hatch_doy_sc +
    (1 | year_f) + (1 | nestcode_rearing),
  data = am_age_fledging_wing,
  control = lmerControl(
    optimizer = "bobyqa",
    optCtrl = list(maxfun = 1e+07)
  )
)
MuMIn::AICc(age_fledg_climmod_quad, age_fledg_climmod_lin)
```

```
##                        df     AICc
## age_fledg_climmod_quad  9 3918.432
## age_fledg_climmod_lin   8 3920.814
```

Models practically the same, not keeping the quadratic effect

```
labels <- c(
  `(Intercept)` = "Intercept",
  hatch_doy_sc = "Hatching day",
```

```
  brood_size = "Hatching brood size",
  signal_for_wing_50 = "PC1 (age: 1-48)",
  `I(signal_for_wing_50^2)` = "PC1 (age: 1-48) 2",
  colonySolothurn = "Colony [Solothurn]"
)

sjPlot::tab_model(
  fledg_surv_climmod_lin,
  age_fledg_climmod_lin,
  file = "output/Tab_short_conseq.doc",
  string.est = "Estimate",
  string.se = "SE",
  string.pred = "Predictors",
  show.ci = FALSE,
  show.se = TRUE,
  show.stat = FALSE,
  show.df = FALSE,
  pred.labels = labels
)
```

Figure

```
eff_data <- data.frame(effects::effect("signal_for_wing_50",
  fledg_surv_climmod_lin,
  partial.residuals = TRUE
))
plot_fledg <- ggplot(eff_data, aes(signal_for_wing_50, fit)) +
  geom_ribbon(aes(ymin = lower, ymax = upper), alpha = .25) +
  geom_line(linewidth = 1) +
  theme(
    legend.position = "none",
    panel.grid.major = element_blank(),
    panel.grid.minor = element_blank(),
    panel.background = element_blank(),
    axis.line = element_line(colour = "black")
  ) +
  xlab("") +
  geom_jitter(
    data = am_nest_clim_wing,
    aes(
      x = signal_for_wing_50,
      y = prop
    ),
    height = 0.03,
    size = 1,
    shape = 19,
    alpha = 0.2
  ) +
  ylab("Nestling survival up to fledging") +
  scale_x_continuous(limits = c(-0.4, 1), breaks = seq(-0.4, 1, 0.2)) +
  scale_y_continuous(limits = c(0, 1.03), breaks = seq(0, 1, 0.2)) +
  annotate("text", x = -0.4, y = 1, label = "(a)")
```

```r
eff_data <- data.frame(effects::effect("signal_for_wing_50",
  age_fledg_climmod_quad,
  partial.residuals = TRUE
))
plot_age_fl <- ggplot(eff_data, aes(signal_for_wing_50, fit)) +
  geom_ribbon(aes(ymin = lower, ymax = upper), alpha = .25) +
  geom_line(linewidth = 1) +
  theme(
    legend.position = "none",
    panel.grid.major = element_blank(),
    panel.grid.minor = element_blank(),
    panel.background = element_blank(),
    axis.line = element_line(colour = "black"),
    axis.title.x = element_markdown()
  ) +
  xlab("Weather conditions (PC1<sub>1-48d</sub>)<br>between 1 and 48 days of age") +
  geom_point(
    data = am_age_fledging_wing,
    aes(
      x = signal_for_wing_50,
      y = age_fledging
    ),
    size = 1,
    shape = 19,
    alpha = 0.2
  ) +
  ylab("Age at fledging (days)") +
  scale_y_continuous(limits = c(50, 76), breaks = seq(45, 90, 5)) +
  scale_x_continuous(limits = c(-0.4, 1), breaks = seq(-0.4, 1, 0.2)) +
  annotate("text", x = -0.4, y = 75, label = "(b)")


cowplot::plot_grid(plot_fledg, plot_age_fl,
  nrow = 2, align = "v"
)
```

```r
# Save the plot to the figures directory
ggsave("figures/Fig. 5.png", width = 4, height = 5, dpi = 300)
```

Session information

On top of using renv, the R session information is printed here for reproducibility.

```r
sessionInfo()
```

```
## R version 4.3.3 (2024-02-29)
## Platform: aarch64-apple-darwin20 (64-bit)
## Running under: macOS Sonoma 14.4.1
##
## Matrix products: default
## BLAS:   /System/Library/Frameworks/Accelerate.framework/Versions/A/Frameworks/vecLib.framework/Versi
```

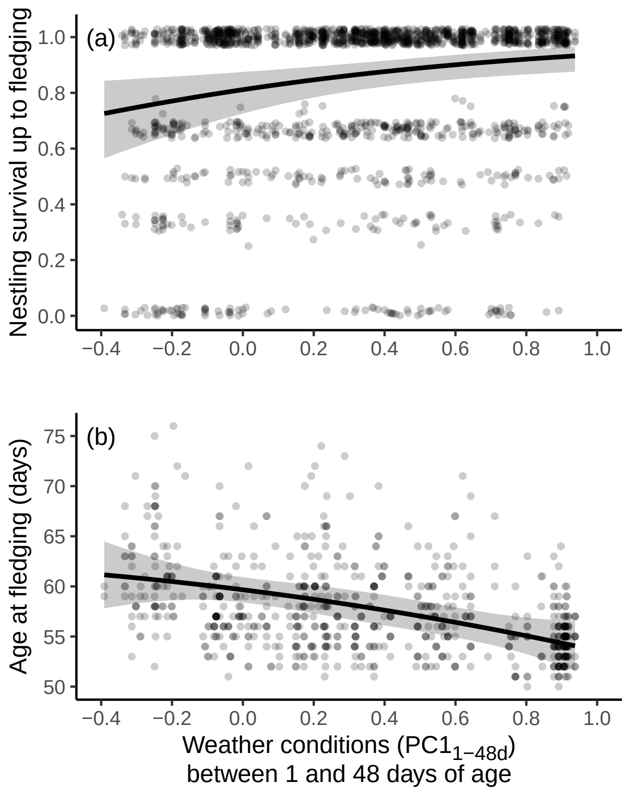

Figure 6: Fig. 5 - Variation in (a) the nestling survival (calculated as the weighted proportion of hatchlings from a nest that successfully fledge) and (b) age at fledging (in days) in relation to weather conditions encountered during development in nestling Alpine swifts. Weather conditions are the resultant of a principal component analysis between daily average temperature and daily rain in the window that best explains the variation in wing length at 50 days. Predicted lines are obtained using average values of the other continuous variables. Climatic windows are reported using the nestling's age as a reference.

```
## LAPACK: /Library/Frameworks/R.framework/Versions/4.3-arm64/Resources/lib/libRlapack.dylib;  LAPACK v
##
## locale:
## [1] en_US.UTF-8/en_US.UTF-8/en_US.UTF-8/C/en_US.UTF-8/en_US.UTF-8
##
## time zone: Europe/Zurich
## tzcode source: internal
##
## attached base packages:
## [1] stats     graphics  grDevices datasets  utils     methods   base
##
## other attached packages:
##  [1] viridis_0.6.5      viridisLite_0.4.2  ggeffects_1.5.2    visreg_2.7.0
##  [5] emmeans_1.10.1     tidyr_1.3.1        performance_0.11.0 sjPlot_2.8.15
##  [9] scales_1.3.0       ggtext_0.1.2       MuMIn_1.47.5       segmented_2.0-3
## [13] nlme_3.1-164       MASS_7.3-60.0.1    climwin_1.2.3      gridExtra_2.3
## [17] ggplot2_3.5.0      lmerTest_3.1-3     lme4_1.1-35.2      Matrix_1.6-5
## [21] lubridate_1.9.3    dplyr_1.1.4
##
## loaded via a namespace (and not attached):
##  [1] DBI_1.2.2                  rlang_1.1.3                magrittr_2.0.3
##  [4] compiler_4.3.3             mgcv_1.9-1                 vctrs_0.6.5
##  [7] quadprog_1.5-8             stringr_1.5.1              pkgconfig_2.0.3
## [10] crayon_1.5.2               fastmap_1.1.1              backports_1.4.1
## [13] labeling_0.4.3             utf8_1.2.4                 rmarkdown_2.26
## [16] markdown_1.12              nloptr_2.0.3               tinytex_0.50
## [19] purrr_1.0.2                xfun_0.43                  jsonlite_1.8.8
## [22] highr_0.10                 reshape_0.8.9              sjmisc_2.8.9
## [25] broom_1.0.5                R6_2.5.1                   stringi_1.8.3
## [28] car_3.1-2                  boot_1.3-30                numDeriv_2016.8-1.1
## [31] estimability_1.5           Rcpp_1.0.12                RcppRoll_0.3.0
## [34] knitr_1.46                 modelr_0.1.11              DHARMa_0.4.6
## [37] zoo_1.8-12                 splines_4.3.3              nnet_7.3-19
## [40] timechange_0.3.0           tidyselect_1.2.1           effects_4.2-2
## [43] abind_1.4-5                yaml_2.3.8                 codetools_0.2-20
## [46] sjlabelled_1.2.0           lattice_0.22-6             tibble_3.2.1
## [49] plyr_1.8.9                 withr_3.0.0                bayestestR_0.13.2
## [52] evaluate_0.23              PerformanceAnalytics_2.0.4 survival_3.5-8
## [55] survey_4.4-2               xts_0.13.2                 xml2_1.3.6
## [58] pillar_1.9.0               carData_3.0-5              renv_1.0.7
## [61] stats4_4.3.3               insight_0.19.10            generics_0.1.3
## [64] munsell_0.5.1              commonmark_1.9.1           minqa_1.2.6
## [67] xtable_1.8-4               glue_1.7.0                 tools_4.3.3
## [70] mvtnorm_1.2-4              cowplot_1.1.3              grid_4.3.3
## [73] mitools_2.4                colorspace_2.1-0           cli_3.6.2
## [76] evd_2.3-6.1                fansi_1.0.6                sjstats_0.18.2
## [79] gtable_0.3.4               digest_0.6.35              farver_2.1.1
## [82] htmltools_0.5.8.1          lifecycle_1.0.4            gridtext_0.1.5
```

References for the uploaded packages

```
packages <- c()
for (i in 1:14) {
  packages[i] <- sessionInfo()$otherPkgs[[i]]$Package
```

```r
  print(citation(packages[i]))
}
```

```
## To cite viridis/viridisLite in publications use:
##
##   Simon Garnier, Noam Ross, Robert Rudis, Antônio P. Camargo, Marco Sciaini, and
##   Cédric Scherer (2024). viridis(Lite) - Colorblind-Friendly Color Maps for R. viridis
##   package version 0.6.5.
##
## A BibTeX entry for LaTeX users is
##
##   @Manual{,
##     title = {{viridis(Lite)} - Colorblind-Friendly Color Maps for R},
##     author = {{Garnier} and {Simon} and {Ross} and {Noam} and {Rudis} and {Robert} and {Camargo} and
##     year = {2024},
##     note = {viridis package version 0.6.5},
##     url = {https://sjmgarnier.github.io/viridis/},
##     doi = {10.5281/zenodo.4679423},
##   }
## To cite viridis/viridisLite in publications use:
##
##   Simon Garnier, Noam Ross, Robert Rudis, Antônio P. Camargo, Marco Sciaini, and
##   Cédric Scherer (2023). viridis(Lite) - Colorblind-Friendly Color Maps for R.
##   viridisLite package version 0.4.2.
##
## A BibTeX entry for LaTeX users is
##
##   @Manual{,
##     title = {{viridis(Lite)} - Colorblind-Friendly Color Maps for R},
##     author = {{Garnier} and {Simon} and {Ross} and {Noam} and {Rudis} and {Robert} and {Camargo} and
##     year = {2023},
##     note = {viridisLite package version 0.4.2},
##     url = {https://sjmgarnier.github.io/viridis/},
##     doi = {10.5281/zenodo.4678327},
##   }
## To cite package 'ggeffects' in publications use:
##
##   Lüdecke D (2018). "ggeffects: Tidy Data Frames of Marginal Effects from Regression
##   Models." _Journal of Open Source Software_, *3*(26), 772. doi:10.21105/joss.00772
##   <https://doi.org/10.21105/joss.00772>.
##
## A BibTeX entry for LaTeX users is
##
##   @Article{,
##     title = {ggeffects: Tidy Data Frames of Marginal Effects from Regression Models.},
##     volume = {3},
##     doi = {10.21105/joss.00772},
##     number = {26},
##     journal = {Journal of Open Source Software},
##     author = {Daniel Lüdecke},
##     year = {2018},
##     pages = {772},
##   }
```

```
## To cite visreg in publications use:
##
##   Breheny P and Burchett W (2017). Visualization of Regression Models Using visreg.
##   The R Journal, 9: 56-71.
##
## A BibTeX entry for LaTeX users is
##
##   @Article{,
##     author = {Patrick Breheny and Woodrow Burchett},
##     title = {Visualization of Regression Models Using visreg},
##     journal = {The R Journal},
##     year = {2017},
##     volume = {9},
##     pages = {56--71},
##     number = {2},
##   }
## To cite package 'emmeans' in publications use:
##
##   Lenth R (2024). _emmeans: Estimated Marginal Means, aka Least-Squares Means_. R
##   package version 1.10.1, <https://CRAN.R-project.org/package=emmeans>.
##
## A BibTeX entry for LaTeX users is
##
##   @Manual{,
##     title = {emmeans: Estimated Marginal Means, aka Least-Squares Means},
##     author = {Russell V. Lenth},
##     year = {2024},
##     note = {R package version 1.10.1},
##     url = {https://CRAN.R-project.org/package=emmeans},
##   }
## To cite package 'tidyr' in publications use:
##
##   Wickham H, Vaughan D, Girlich M (2024). _tidyr: Tidy Messy Data_. R package version
##   1.3.1, <https://CRAN.R-project.org/package=tidyr>.
##
## A BibTeX entry for LaTeX users is
##
##   @Manual{,
##     title = {tidyr: Tidy Messy Data},
##     author = {Hadley Wickham and Davis Vaughan and Maximilian Girlich},
##     year = {2024},
##     note = {R package version 1.3.1},
##     url = {https://CRAN.R-project.org/package=tidyr},
##   }
## To cite package 'performance' in publications use:
##
##   Lüdecke et al., (2021). performance: An R Package for Assessment, Comparison and
##   Testing of Statistical Models. Journal of Open Source Software, 6(60), 3139.
##   https://doi.org/10.21105/joss.03139
##
## A BibTeX entry for LaTeX users is
##
##   @Article{,
##     title = {{performance}: An {R} Package for Assessment, Comparison and Testing of Statistical Mode
```

```
##     author = {Daniel Lüdecke and Mattan S. Ben-Shachar and Indrajeet Patil and Philip Waggoner and D
##     year = {2021},
##     journal = {Journal of Open Source Software},
##     volume = {6},
##     number = {60},
##     pages = {3139},
##     doi = {10.21105/joss.03139},
##   }
## To cite package 'sjPlot' in publications use:
##
##   Lüdecke D (2023). _sjPlot: Data Visualization for Statistics in Social Science_. R
##   package version 2.8.15, <https://CRAN.R-project.org/package=sjPlot>.
##
## A BibTeX entry for LaTeX users is
##
##   @Manual{,
##     title = {sjPlot: Data Visualization for Statistics in Social Science},
##     author = {Daniel Lüdecke},
##     year = {2023},
##     note = {R package version 2.8.15},
##     url = {https://CRAN.R-project.org/package=sjPlot},
##   }
## To cite package 'scales' in publications use:
##
##   Wickham H, Pedersen T, Seidel D (2023). _scales: Scale Functions for Visualization_.
##   R package version 1.3.0, <https://CRAN.R-project.org/package=scales>.
##
## A BibTeX entry for LaTeX users is
##
##   @Manual{,
##     title = {scales: Scale Functions for Visualization},
##     author = {Hadley Wickham and Thomas Lin Pedersen and Dana Seidel},
##     year = {2023},
##     note = {R package version 1.3.0},
##     url = {https://CRAN.R-project.org/package=scales},
##   }
## To cite package 'ggtext' in publications use:
##
##   Wilke C, Wiernik B (2022). _ggtext: Improved Text Rendering Support for 'ggplot2'_.
##   R package version 0.1.2, <https://CRAN.R-project.org/package=ggtext>.
##
## A BibTeX entry for LaTeX users is
##
##   @Manual{,
##     title = {ggtext: Improved Text Rendering Support for 'ggplot2'},
##     author = {Claus O. Wilke and Brenton M. Wiernik},
##     year = {2022},
##     note = {R package version 0.1.2},
##     url = {https://CRAN.R-project.org/package=ggtext},
##   }
## To cite package 'MuMIn' in publications use:
##
##   Bartoń K (2023). _MuMIn: Multi-Model Inference_. R package version 1.47.5,
##   <https://CRAN.R-project.org/package=MuMIn>.
```

```
##
## A BibTeX entry for LaTeX users is
##
##   @Manual{,
##     title = {MuMIn: Multi-Model Inference},
##     author = {Kamil Bartoń},
##     year = {2023},
##     note = {R package version 1.47.5},
##     url = {https://CRAN.R-project.org/package=MuMIn},
##   }
##
## ATTENTION: This citation information has been auto-generated from the package
## DESCRIPTION file and may need manual editing, see 'help("citation")'.
## To cite segmented in publications use one or more of the following papers:
##
##   Fasola S, Muggeo VMR, Kuchenhoff K. (2018). A heuristic, iterative algorithm for
##   change-point detection in abrupt change models. Computational Statistics, 33,
##   997-1015.
##
##   Muggeo VMR (2003). Estimating regression models with unknown break-points.
##   Statistics in Medicine, 22, 3055-3071.
##
##   Muggeo VMR (2008). segmented: an R Package to Fit Regression Models with Broken-Line
##   Relationships. R News, 8/1, 20-25. URL https://cran.r-project.org/doc/Rnews/.
##
##   Muggeo VMR (2016). Testing with a nuisance parameter present only under the
##   alternative: a score-based approach with application to segmented modelling. J of
##   Statistical Computation and Simulation, 86, 3059-3067.
##
##   Muggeo VMR (2017). Interval estimation for the breakpoint in segmented regression: a
##   smoothed score-based approach. Australian \& New Zealand Journal of Statistics, 59,
##   311-322.
##
##   Muggeo VMR, Atkinhs DC, Gallopp RJ, Dimidjian S (2014). Segmented mixed models with
##   random changepoints: a maximum likelihood approach with application to treatment for
##   depression study Statistical Modelling, 14, 293-313.
##
## To see these entries in BibTeX format, use 'print(<citation>, bibtex=TRUE)',
## 'toBibtex(.)', or set 'options(citation.bibtex.max=999)'.
## To cite package 'nlme' in publications use:
##
##   Pinheiro J, Bates D, R Core Team (2023). _nlme: Linear and Nonlinear Mixed Effects
##   Models_. R package version 3.1-164, <https://CRAN.R-project.org/package=nlme>.
##
##   Pinheiro JC, Bates DM (2000). _Mixed-Effects Models in S and S-PLUS_. Springer, New
##   York. doi:10.1007/b98882 <https://doi.org/10.1007/b98882>.
##
## To see these entries in BibTeX format, use 'print(<citation>, bibtex=TRUE)',
## 'toBibtex(.)', or set 'options(citation.bibtex.max=999)'.
## To cite the MASS package in publications use:
##
##   Venables, W. N. & Ripley, B. D. (2002) Modern Applied Statistics with S. Fourth
##   Edition. Springer, New York. ISBN 0-387-95457-0
##
```

```
## A BibTeX entry for LaTeX users is
##
##   @Book{,
##     title = {Modern Applied Statistics with S},
##     author = {W. N. Venables and B. D. Ripley},
##     publisher = {Springer},
##     edition = {Fourth},
##     address = {New York},
##     year = {2002},
##     note = {ISBN 0-387-95457-0},
##     url = {https://www.stats.ox.ac.uk/pub/MASS4/},
##   }
```
